# IGFBP6 orchestrates antiinfective immune collapse in murine sepsis via prohibitin-2–mediated immunosuppression

**DOI:** 10.1172/JCI184721

**Published:** 2025-09-02

**Authors:** Kai Chen, Ying Hu, Xiaoyan Yu, Hong Tang, Yanting Ruan, Yue Li, Xun Gao, Qing Zhao, Hong Wang, Xuemei Zhang, David Paul Molloy, Yibing Yin, Dapeng Chen, Zhixin Song

**Affiliations:** 1 Department of Clinical Laboratory, Children’s Hospital of Chongqing Medical University, National Clinical Research Center for Child Health and Disorders, Ministry of Education Key Laboratory of Child Development and Disorders, China International Science and Technology Cooperation Base of Child Development and Critical Disorders, Chongqing Key Laboratory of Pediatric Metabolism and Inflammatory Diseases, Chongqing, China.; 2 Department of Laboratory Medicine, Key Laboratory of Diagnostic Medicine, Chongqing Medical University, Chongqing, China.; 3 Department of Critical Care Medicine, Department of Surgical Intensive Care Unit, the First Affiliated Hospital of Chongqing Medical University, Chongqing, China.; 4 Molecular Medicine and Cancer Research Center, College of Basic Medical Sciences, Chongqing Medical University, Chongqing, China.; 5 Center of Clinical Laboratory Medicine, Zhongda Hospital, Southeast University, Nanjing, Jiangsu, China.; 6 Department of Laboratory Medicine, the Second Affiliated Hospital of Chongqing Medical, University, Chongqing, China.; 7 Department of Biochemistry and Molecular Biology, College of Basic Medical Sciences, Chongqing Medical University, Chongqing, China.

**Keywords:** Infectious disease, Inflammation, Immunotherapy, Macrophages

## Abstract

The persistent challenge of sepsis-related mortality underscores the necessity for deeper insights. Our multicenter, cross-age cohort study identified insulin-like growth factor binding protein 6 (IGFBP6) as a critical regulator in sepsis diagnosis, prognosis, and mortality risk evaluation. Mechanistically, IGFBP6 engages in IGF-independent binding to prohibitin2 (PHB2) on epithelial cells, driving PHB2 tyrosine phosphorylation during sepsis. This process disrupts STAT1 phosphorylation, nuclear translocation, and its recruitment to the CCL2 promoter, ultimately impairing CCL2 transcription and macrophage chemotaxis. Crucially, PHB2 silencing via siPHB2 and STAT1 activation using 2-NP restored CCL2 expression in vitro and in vivo, improving bacterial clearance and survival in septic mice. Concurrently, IGFBP6 compromised macrophage bactericidal activity by inhibiting Akt phosphorylation, reducing ROS/IL-1β production and phagocytic capacity — defects reversible by Akt agonist SC79. Collectively, IGFBP6 emerges as an endogenous driver of sepsis pathogenesis, positioning it as a dual diagnostic biomarker and therapeutic target. Intervention strategies targeting IGFBP6-mediated signaling may offer transformative approaches for sepsis management.

## Abstract 2

IGFBP6 orchestrates sepsis pathogenesis via PHB2-dependent immunosuppression and macrophage dysfunction, establishing it as a diagnostic-therapeutic nexus.

## Graphical abstract

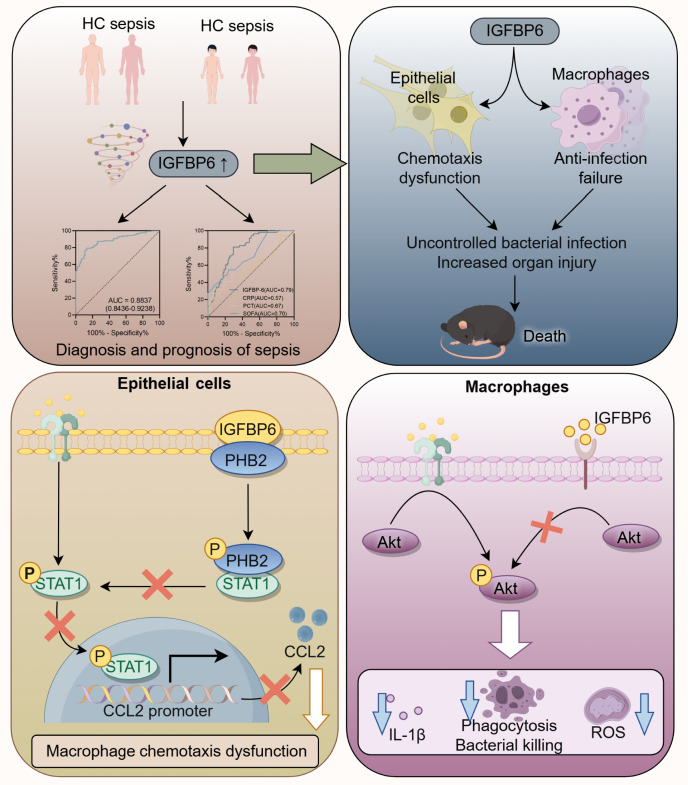

## Introduction

Sepsis is the leading life-threatening condition and cause of death in hospital intensive care units (ICUs), with a landmark multicenter study documenting 48.9 million annual cases and 11 million sepsis-attributed deaths — accounting for 20% of global mortality and claiming lives at a rate of 1 every 2.8 seconds, underscoring its persistent status as an urgent public health crisis (1, 2). Although organizations including the Global Sepsis Alliance and WHO have advanced public awareness, research initiatives, and clinical protocols in recent decades (3–6), the persistently high fatality rates emphasize the unmet need for emerging therapeutic strategies (2).

Sepsis pathogenesis is fundamentally driven by intrinsic mechanisms involving multilevel immune cell dysfunction and breakdown of inflammatory homeostasis. Our recent investigation identified a previously unrecognized pathological contributor to septic progression — insulin-like growth factor binding protein 6 (IGFBP6). As an O-linked glycoprotein within the conserved IGFBP family (IGFBP1–7), IGFBP6 shares structural homology with its paralogs while demonstrating unique functional characteristics (7). Although most IGFBPs classically modulate cellular proliferation, differentiation, and metabolism by binding to IGF-I/II, IGFBP6 exhibits preferential binding to IGF-II, effectively antagonizing IGF-II–induced cell proliferation (8), differentiation (9), migration (10, 11), survival (12), and angiogenesis (11).

Emerging evidence now positions IGFBP6 as a pleiotropic immunomodulator operating through both IGF-II–dependent and –independent pathways. Beyond its canonical growth factor regulation, this multifunctional protein demonstrates direct involvement in immune cell activation dynamics (13), cell migration (10, 13, 14), apoptotic regulation (15), and oxidative stress (16) in an IGF-II–independent manner. Notably, its identification as a stress-responsive acute-phase protein (17) suggests rapid deployment during systemic inflammation to coordinate cross-tissue immune communication. Despite these immunoregulatory connections, the functional implications of IGFBP6 in sepsis pathophysiology remained unexplored, to our knowledge, until our study uncovered its critical role in driving septic progression through distinct immune modulation mechanisms.

## Results

### Characteristics of study participants.

**Figure 1:**
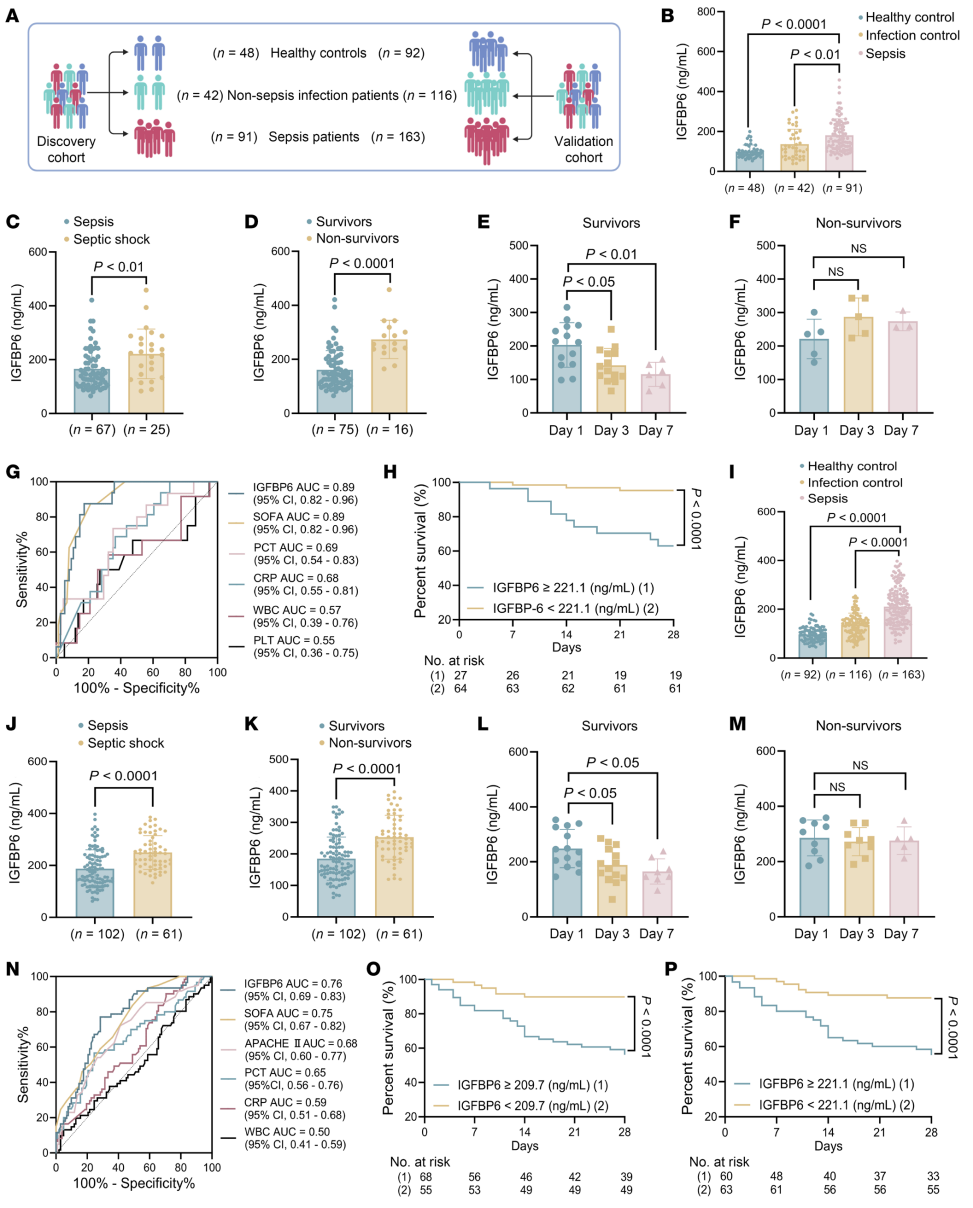
IGFBP6 as a diagnostic and prognostic biomarker in adult sepsis. (**A**) Discovery cohort (sepsis: *n* = 91; non-septic infection controls: *n* = 42; healthy controls: *n* = 48). Validation cohort (sepsis: *n* = 163; non-septic infection controls: *n* = 116; healthy controls: *n* = 92). (**B**) Serum IGFBP6 levels in discovery cohort (ELISA). (**C**) Comparison of serum IGFBP6 levels in patients with sepsis (*n* = 65) and patients with septic shock (*n* = 26) in discovery cohort. (**D**) Serum IGFBP6 levels in survivors with sepsis (*n* = 75) versus non-survivors with sepsis (*n* = 16) in discovery cohort. (**E** and **F**) Dynamics of serum IGFBP6 levels in randomly selected survivors (**E**) and non-survivors (**F**) at days 1, 3, or 7. (**G**) ROC analysis for mortality prediction comparing IGFBP6, SOFA score, PCT/CRP concentrations, and WBC and platelet counts (discovery cohort). (**H**) Kaplan-Meier survival curves stratified by IGFBP6 cutoff value (221.1 ng/mL) at ICU admission (discovery cohort). (**I**) Serum IGFBP6 levels in validation cohort (ELISA). (**J**) IGFBP6 levels in patients with sepsis (*n* = 102) versus septic shock (*n* = 61) (validation cohort). (**K**) Serum IGFBP6 levels in survivors (*n* = 102) versus non-survivors (*n* = 61) in validation cohort. (**L** and **M**) Dynamics of IGFBP6 levels during 1, 3, and 7 days of ICU admission in validation cohort survivors (**L**) and non-survivors (**M**). (**N**) ROC curves comparing IGFBP6, SOFA, APACHE II, PCT, CRP, and WBC for mortality prediction (validation cohort). (**O** and **P**) Kaplan-Meier survival analysis using IGFBP6 cutoffs of 209.7 ng/mL (**O**) and 221.1 ng/mL (**P**) in validation cohort. Numbers and percentages indicate individuals; horizontal bars in **B**–**F** and **I**–**M** represent mean; Student’s *t* test in **C**, **D**, **J**, and **K**; 1-way ANOVA in **B**, **E**, **F**, **I**, **L**, **M**; log-rank test in **H**, **O**, and **P**. CRP, C-reactive protein; ICU, intensive care unit; PCT, procalcitonin; SOFA, sequential organ failure assessment.

To characterize the relationship between IGFBP6 and the progression of sepsis, we constructed cross-age, multicenter sepsis cohorts. The adult discovery cohort comprised 91 patients with sepsis, 42 patients with non-sepsis infections, and 48 healthy volunteers (Figure 1A and Supplemental Table 1; supplemental material available online with this article; https://doi.org/10.1172/JCI184721DS1). No significant differences were observed between survivors and non-survivors in gender, age, WBC counts, C-reactive protein (CRP) or procalcitonin (PCT) levels, platelet counts, or ICU hospitalization duration. However, a significant difference of sequential (sepsis-related) organ failure assessment (SOFA) scores and septic shock incidence was observed between these patients. The adult validation cohort comprised 163 patients with sepsis, 116 patients with non-sepsis infections, and 92 healthy volunteers (Figure 1A and Supplemental Table 2). Similar to the discovery cohort, survivors and non-survivors showed no differences in gender, age, WBC numbers, CRP/PCT levels, or ICU stay duration but displayed significant disparities in SOFA scores, APACHE II scores, and septic shock status at ICU admission.

**Figure 2:**
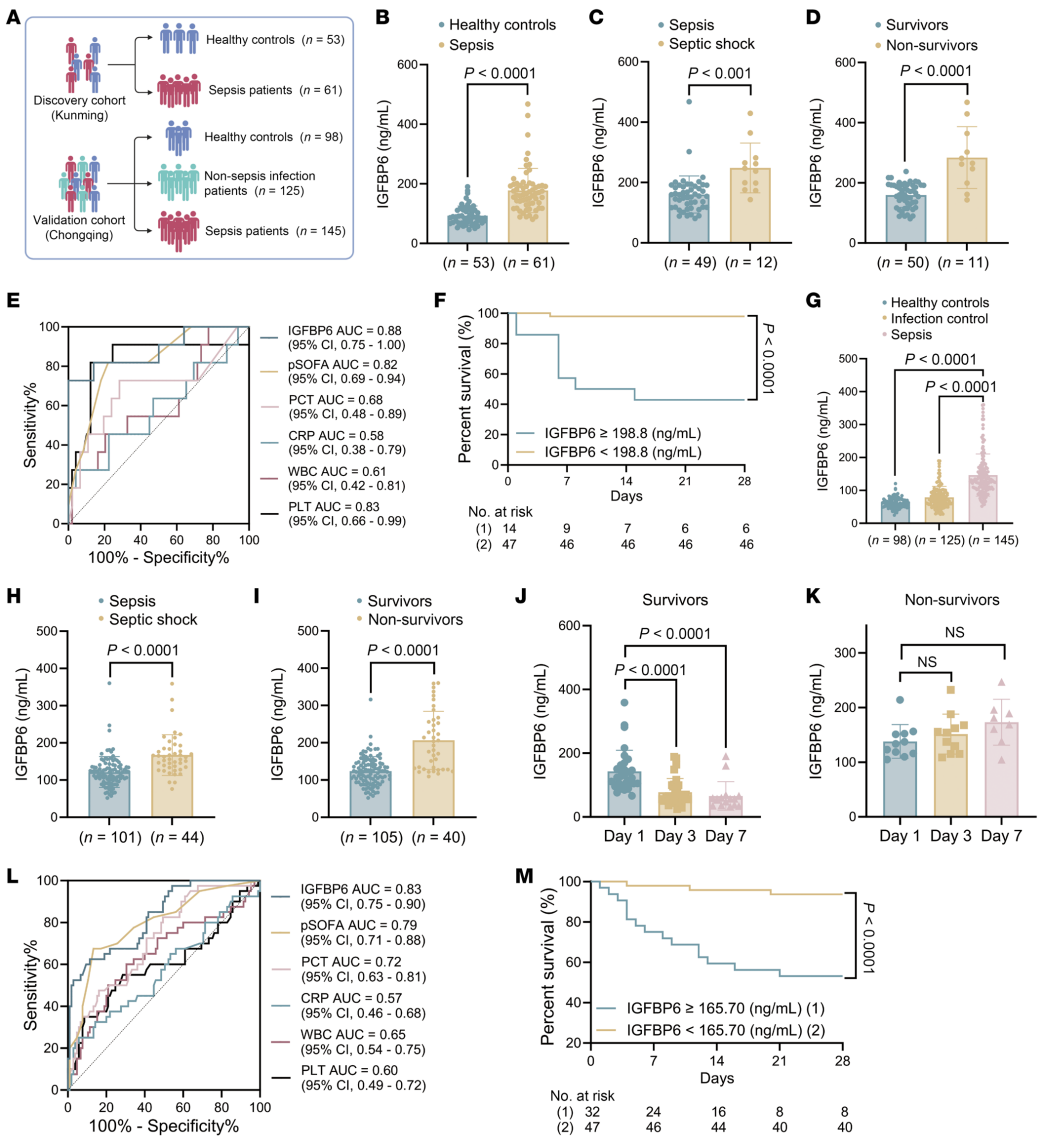
IGFBP6 as a diagnostic and prognostic biomarker in pediatric sepsis. (**A**) Cohort design: pediatric discovery cohort (sepsis: *n* = 61; healthy controls: *n* = 53) and validation cohort (sepsis: *n* = 145; non-septic infection controls: *n* = 125; healthy controls: *n* = 98). (**B**) Serum IGFBP6 levels (ELISA) in discovery cohort. (**C**) Comparison of serum IGFBP6 levels in patients with sepsis (*n* = 49) versus septic shock (*n* = 12) in discovery cohort. (**D**) Serum IGFBP6 levels in pediatric septic survivors (*n* = 50) versus non-survivors (*n* = 11) in discovery cohort. (**E**) ROC analysis for mortality prediction comparing IGFBP6, PCT/CRP levels, and WBC and platelet counts in discovery cohort. (**F**) Kaplan-Meier survival curves stratified by the IGFBP6 cutoff value (198.9 ng/mL) at ICU admission (discovery cohort). (**G**) Serum IGFBP6 levels (ELISA) in validation cohort. (**H**) Serum IGFBP6 levels in patients with sepsis (*n* = 101) versus septic shock (*n* = 44) (validation cohort). (**I**) Serum IGFBP6 levels in survivors (*n* = 105) versus non-survivors (validation cohort). (**J** and **K**) The dynamics of IGFBP6 levels in validation cohort survivors (**J**) and non-survivors (**K**) at days 1, 3, and 7 of ICU admission. (**L**) ROC curves comparing IGFBP6, pSOFA scores, PCT/CRP levels, and WBC and platelet counts for mortality prediction (validation cohort). (**M**) Kaplan-Meier survival analysis using IGFBP6 cutoff value (165.7 ng/mL; validation cohort). Numbers and percentages indicate individuals, and horizontal bars in **B**–**K** except **E** and **F** represent mean values; Student’s *t* test in **B**–**D** and **H**–**K**; 1-way ANOVA in **G**, **J**, **K**; log-rank test in **E** and **M**. CRP, C-reactive protein; ICU, intensive care unit; PCT, procalcitonin; pSOFA, pediatric sequential organ failure assessment.

In the pediatric cohort, the discovery cohort comprised 61 patients with sepsis and 53 healthy volunteers (Figure 2A and Supplemental Table 3); the validation cohort included 145 patients with sepsis, 125 patients with non-sepsis infections, and 98 healthy volunteers (Figure 2A and Supplemental Table 4). Across both pediatric cohorts, survivors and non-survivors demonstrated comparable gender distribution, age, WBC counts, CRP levels, and ICU duration. Notably, significant differences were observed in pediatric SOFA (pSOFA) scores, PCT concentrations, and septic shock status at ICU admission between survivors and non-survivors.

### IGFBP6 is a diagnostic and prognostic biomarker for sepsis across age groups.

In adult cohorts, serum IGFBP6 levels at admission were significantly elevated in patients with sepsis versus patients with non-sepsis infections and healthy volunteers across the discovery (Figure 1B) and validation cohorts (Figure 1I). Stratified analysis revealed significantly higher concentrations of IGFBP6 in septic shock than in sepsis (Figure 1, C and J) and in nonsurvivors than in survivors (Figure 1, D and K). Longitudinal monitoring demonstrated dynamic patterns: survivors exhibited progressive IGFBP6 reduction over days 1, 3, and 7 after admission (Figure 1, E and L), contrasting with sustained elevation in non-survivors (Figure 1, F and M). Serum IGFBP6 levels at admission positively correlated with SOFA score, PCT, and CRP levels in the discovery cohort (Supplemental Figure 1, A–E) and additionally with APACHE II scores and platelet counts in validation cohorts (Supplemental Figure 1, F–K). ROC curve analysis confirmed robust diagnostic capacity for sepsis: the AUC was 0.85 (95% CI, 0.79 to 0.92) in the discovery cohort (Supplemental Figure 2A) and 0.93 (95% CI, 0.90 to 0.96) in the validation cohort (Supplemental Figure 2C), demonstrating marginally higher diagnostic performance compared with the literature-reported AUC of 0.86 for PCT in sepsis diagnosis (18, 19). The AUC of IGFBP6 for differential diagnosis of sepsis and non-septic infection was 0.67 (95% CI, 0.57 to 0.78) (Supplemental Figure 2B) and 0.78 (95% CI, 0.73 to 0.84) (Supplemental Figure 2D), respectively.

Univariate and multivariate Cox regression identified both serum IGFBP6 level and SOFA scores as independent predictors for 28-day mortality (Supplemental Tables 5 and 6). In the discovery cohort, IGFBP6 and SOFA scores showed identical AUC values of 0.89 (95% CI, 0.82 to 0.96) for predicting mortality, outperforming the CRP, PCT, WBC, and platelet measures (Figure 1G and Supplemental Table 7). In the validation cohort, IGFBP6 maintained predictive utility with an AUC of 0.76 (95% CI, 0.69 to 0.83), surpassing SOFA, APACHE II, CRP, PCT, and WBC (Figure 1N and Supplemental Table 8). Thereafter, the Kaplan-Meier survival curve analysis using cohort-specific cutoff values (221.1 ng/mL discovery cohort, 209.7 ng/mL validation cohort) showed that patients with lower serum IGFBP6 levels had significantly better survival outcomes than those with elevated levels in both the discovery cohort (Figure 1, G and O) and validation cohort (Figure 1P).

Parallel findings emerged in pediatric cohorts. Serum IGFBP6 levels were markedly elevated in pediatric patients with sepsis versus healthy controls (Figure 2, B and G), with hierarchical increases in septic shock (Figure 2, C and H) and in the number of non-survivors (Figure 2, D and I). In the validation cohort, survivors demonstrated progressive IGFBP6 decline (Figure 2J), contrasting with persistent elevation in non-survivors (Figure 2K). In the discovery cohort, serum IGFBP6 levels at admission correlated with pSOFA scores, PCT/CRP levels, and platelet counts (Supplemental Figure 3, A–J). ROC curve analysis confirmed the superior diagnostic accuracy of IGFBP6 for pediatric sepsis, with the discovery and validation cohorts showing AUCs of 0.90 (95% CI, 0.85-0.96) and 0.96 (95% CI, 0.94–0.98), respectively (Supplemental Figure 2, E and F). The biomarker also showed discriminative power between sepsis and non-sepsis infections (AUC 0.87, 0.82–0.91; Supplemental Figure 2G).

Univariate and multivariate models confirmed IGFBP6 as an independent mortality predictor (Supplemental Tables 9 and 10), with higher predictive AUC (0.88 [95% CI, 0.75 to 1.00], Figure 2E and 0.83 [95% CI, 0.75 to 0.90], Figure 2L) versus pSOFA and conventional biomarkers (Supplemental Tables 11 and 12). Survival stratification based on admission IGFBP6 thresholds significantly differentiated mortality outcomes across both the discovery (Figure 2F) and validation (Figure 2M) cohorts, mirroring observations in adult populations.

### Depletion of endogenous IGFBP6 improves outcomes in murine polymicrobial sepsis.

**Figure 3:**
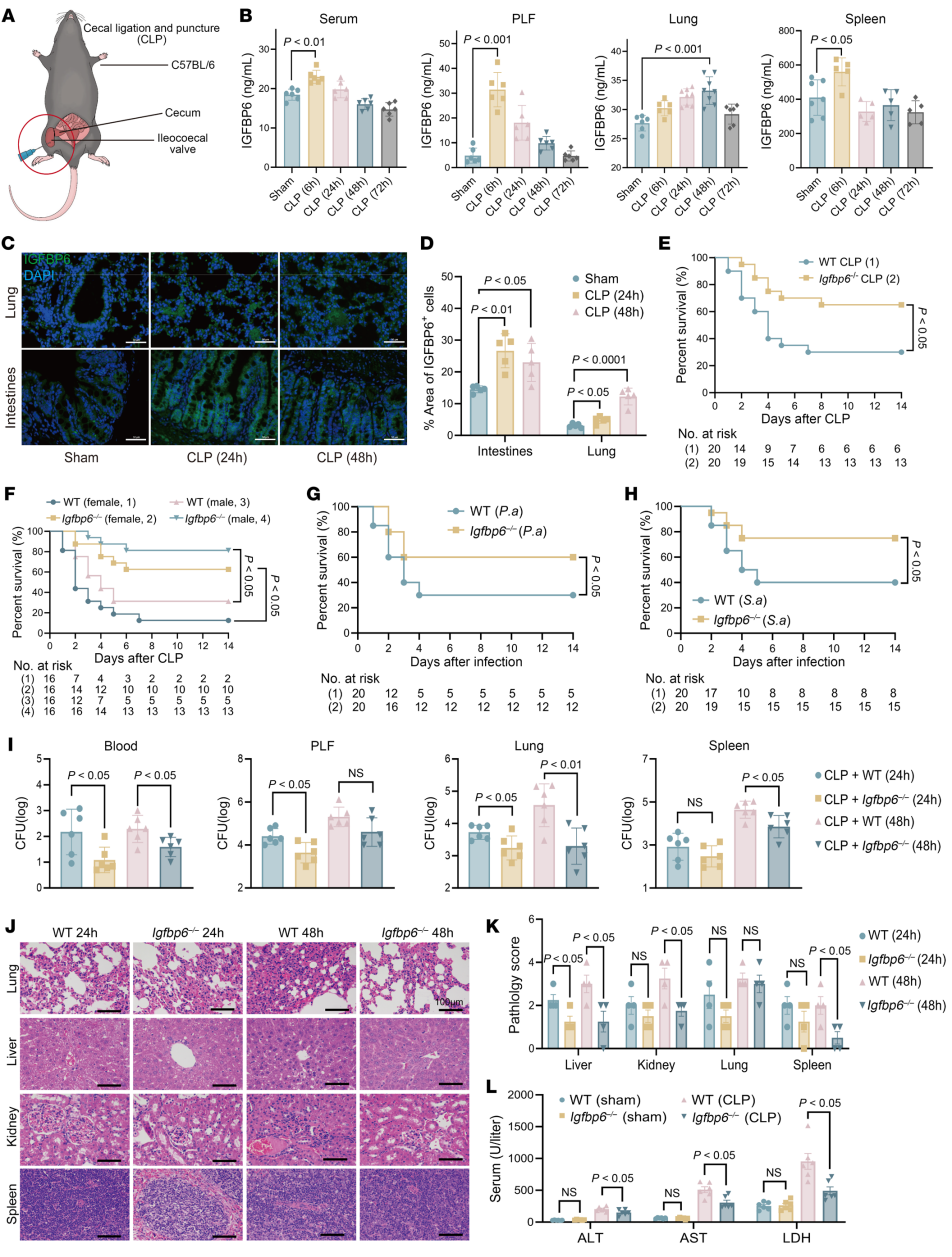
Genetic ablation of IGFBP6 improves sepsis outcomes in murine models. (**A**) Experimental design: Male C57BL/6N mice underwent CLP (24-gauge needle) or sham surgery. (**B**) IGFBP6 levels (ELISA) in serum, PLF, lung, and spleen after CLP (*n* = 5–8/group) at different time points (6, 24, 48, and 72 h). (**C**) Representative fluorescence images of IGFBP6 expression in lung and intestinal tissue after CLP. FITC-conjugated anti-IGFBP6 (1:1,000) and DAPI (1 μg/mL) were used to label IGFBP6 and the nucleus. Scale bar: 50 μm. (**D**) Quantitative fluorescence intensity for **C**. (**E**) Comparison of survival between WT and *Igfbp6^–/–^* mice after CLP (*n* = 20/group). (**F**) Sex-matched survival analysis in WT and *Igfbp6^–/–^* mice after CLP (*n* = 16/group; 14-day monitoring). (**G** and **H**) Comparison of survival between WT and *Igfbp6^–/–^* mice challenged with *P*. *aeruginosa* (5 × 10^7^ CFU, **G**) or *S*. *aureus* (3 × 10^8^ CFU, **H**) (*n* = 20/group). (**I**) Bacterial loads in PLF, blood, lungs, and spleens of WT and *Igfbp6^–/–^* mice at 24/48 hours after CLP (*n* = 6/group). (**J**) Representative H&E staining images of lung, liver, spleen, and kidney tissues of WT and *Igfbp6^–/–^* mice at 24/48 hours after CLP (scale bar: 100 μm). (**K**) Histopathological scoring of organ damage for **J** (*n* = 4/group). (**L**) Serological biomarkers of organ injury (ALT, AST, LDH) in WT or *Igfbp6^–/–^* mice at 24/48 hours after CLP (*n* = 6/group). All data are representative of 3 independent experiments. Each dot in graphs represents individual biological replicate. Student’s *t* test in **I**–**L**; 1-way ANOVA in **B** and **D**; log-rank test in **E**–**H**; NS, not significant. ALT, alanine aminotransferase; AST, aspartate aminotransferase; CLP, cecal ligation and puncture; LDH, lactate dehydrogenase [LDH].

To investigate the role of IGFBP6 in sepsis pathogenesis, we established a murine sepsis model induced by cecal ligation and puncture (CLP) and observed significant elevation of IGFBP6 levels in the serum, peritoneal lavage fluid (PLF), and key organs (lung and spleen) during sepsis progression (Figure 3, A and B). Immunofluorescence analysis further confirmed enhanced IGFBP6 expression in the pulmonary and intestinal tissues of septic mice (Figure 3, C and D). Cellular origin studies revealed that bacterial stimulation induced marked IGFBP6 upregulation in macrophages and primary lung/intestinal epithelial cells within 48 hours, while neutrophil expression decreased (Supplemental Figure 4, A–D). Consistent findings were observed in mouse lung epithelial (MLE-12) and intestinal epithelial (MODE-K) lines (Supplemental Figure 4, D and E), suggesting epithelial cells and macrophages constitute primary sources of systemic IGFBP6 elevation during sepsis. Notably, TLR signaling pathway analysis revealed that TLR2 and/or TLR4 deficiency substantially attenuated IGFBP6 expression in PLF, pulmonary tissue, macrophages, and epithelial cells during sepsis (Supplemental Figure 5, A–E), establishing TLR2/4-mediated regulation of IGFBP6 production.

To delineate the pathophysiological implications of IGFBP6 elevation, we employed an IGFBP6 knockout (*Igfbp6^–/–^*) mouse in polymicrobial sepsis models. Genetic ablation of IGFBP6 conferred significant survival advantages in CLP-induced polymicrobial sepsis (Figure 3E) in a sex-independent manner (Figure 3F). This protective effect extended to monomicrobial sepsis induced by clinically relevant pathogens (20, 21) *Staphylococcus* *aureus* (Gram-positive) and *Pseudomonas* *aeruginosa* (Gram-negative) (Figure 3, G and H). Effective and rapid bacterial clearance is a fundamental determinant of outcomes in sepsis (22, 23). Enhanced bacterial clearance in *Igfbp6^–/–^* mice was evidenced by reduced microbial burdens in PLF, spleen, blood, and lung samples compared with WT controls (Figure 3I).

Considering vital organ dysfunction and damage due to sepsis (24), multiple organ damage was assessed. Histopathological analysis demonstrated attenuated multiorgan damage in knockout mice, with significantly reduced injury scores in lung, liver, spleen, and kidney after CLP (Figure 3, J and K). Correspondingly, biochemical biomarkers of hepatocellular injury (alanine aminotransferase [ALT] and aspartate aminotransferase [AST]) and generalized cellular damage (lactate dehydrogenase [LDH]) were markedly decreased in *Igfbp6^–/–^* mice relative to WT controls (Figure 3L), collectively indicating that IGFBP6 depletion mitigates sepsis-associated organ dysfunction.

### IGFBP6 facilitates the pathogenesis and progression of sepsis.

**Figure 4:**
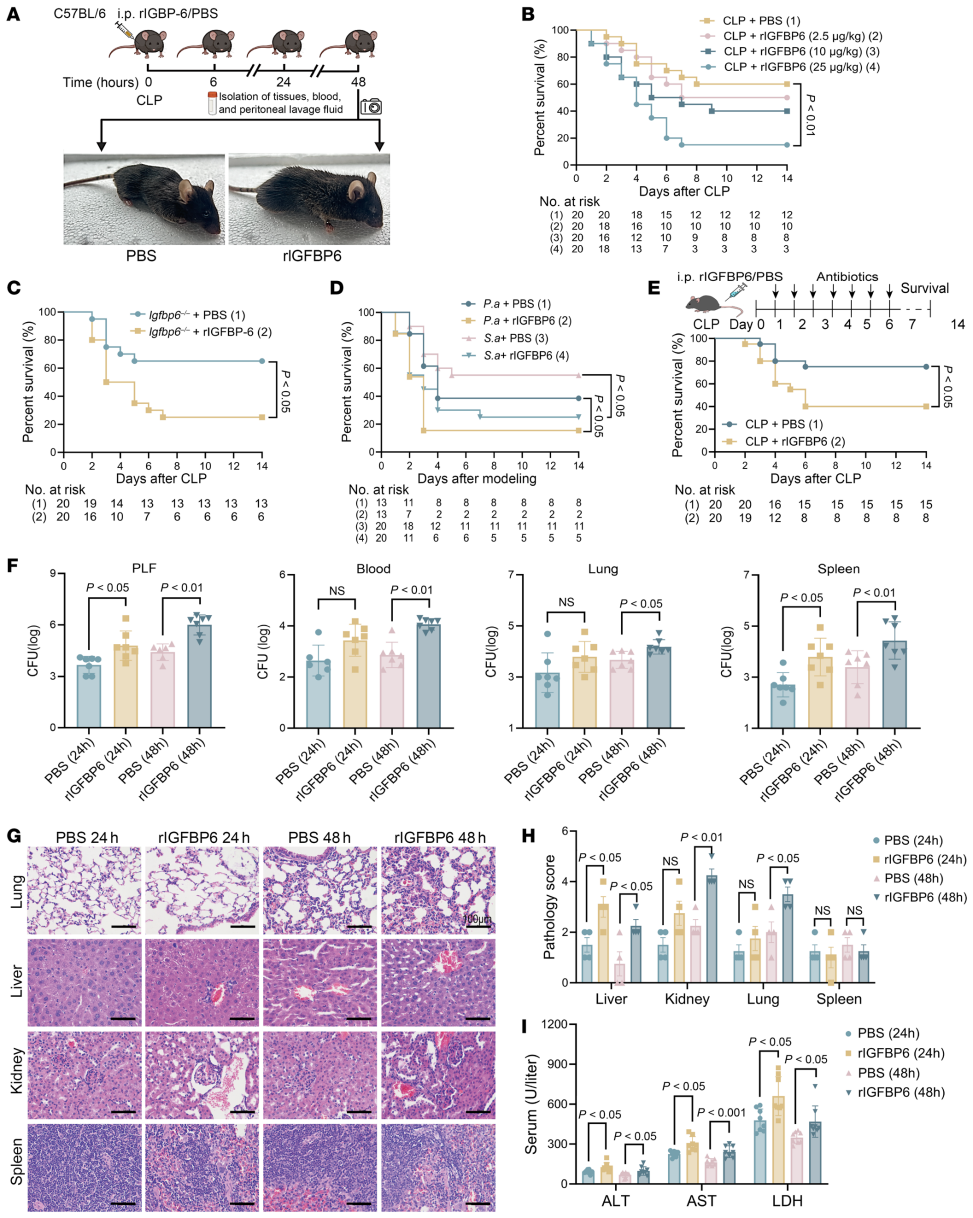
IGFBP6 promotes the occurrence and progression of sepsis. (**A**) The study protocol is shown. rIGFBP6 or PBS control was i.p. administered to C57BL/6N mice after CLP. (**B**) Dose-dependent survival outcomes in septic mice treated with rIGFBP6 (2.5, 10, 25 μg/kg) or PBS (*n* = 20/group). (**C**) Survival curves of septic *Igfbp6^–/–^* mice treated with rIGFBP6 or PBS (*n* = 20/group). (**D**) Survival curves of septic mice treated with meropenem for 7 days after CLP in PBS and rIGFBP6 groups (*n* = 20/group). (**E**) Survival curves of mice challenged with *P*. *aeruginosa* (5 × 10^7^ CFU) or *S*. *aureus* (3 × 10^8^ CFU) after rIGFBP6 administration (*n* = 13/group). (**F**) Bacterial loads in the PLF, blood, lungs, and spleens of mice treated with rIGFBP6 or PBS at 24/48 hours after CLP (*n* = 7/group). (**G**) Representative H&E staining images of lung, liver, spleen, and kidney tissues from mice treated with rIGFBP6 or PBS 24/48 hours after CLP (scale bar: 100 μm). (**H**) Histopathological quantification of organ damage for **G** (*n* = 4/group). (**I**) Serological biomarkers of organ injury (ALT, AST, LDH) in PBS- or rIGFBP6-treated mice (*n* = 8 per group) at 24/48 hours after CLP. All data are representative of 3 independent experiments. Each dot in graphs represents data from an individual biological replicate. Student’s *t* test in **F**–**I**; log-rank test in **B**–**E**; NS, not significant. ALT, alanine aminotransferase; AST, aspartate aminotransferase; CLP, cecal ligation and puncture; LDH, lactate dehydrogenase. PLF, peritoneal lavage fluid.

To elucidate the pathophysiological contributions of IGFBP6 in sepsis progression, recombinant IGFBP6 protein (rIGFBP6) was used for in vivo experiments (Figure 4A). Exogenous rIGFBP6 administration significantly exacerbated mortality in both WT and *Igfbp6^–/–^* septic mice compared with PBS-treated controls (Figure 4, B and C). This detrimental effect was consistently replicated in pathogen-specific sepsis paradigms induced by intraperitoneal injection of *P*. *aeruginosa* and *S*. *aureus* (Figure 4D). After antibiotic intervention, PBS-treated controls exhibited an 80% survival rate, whereas rIGFBP6 administration resulted in a markedly reduced survival rate of 40% (Figure 4E). Notably, persistent unresolved infections were observed in rIGFBP6-treated mice even after 7 days of antibiotic therapy (Supplemental Figure 6). Consistent with survival deficits, increased bacterial loads were also observed in samples of PLF, spleen, blood, and lung from rIGFBP6-treated septic WT mice compared with PBS controls (Figure 4F). Histopathological evaluation revealed enhanced multiorgan inflammatory damage in rIGFBP6-treated septic mice, with significantly aggravated injury scores in lung, liver, spleen, and kidney at 24 hours and 48 hours after CLP (Figure 4, G and H). Concurrently, serum biochemical biomarkers of tissue damage (ALT, AST, LDH) were also significantly elevated in rIGFBP6-treated mice at both time points (Figure 4I). These findings collectively indicate that elevated IGFBP6 levels compromise host antimicrobial defenses, aggravate multiorgan damage, and ultimately drive sepsis mortality.

### IGFBP6 impairs the chemotaxis of macrophages in sepsis pathogenesis.

**Figure 5:**
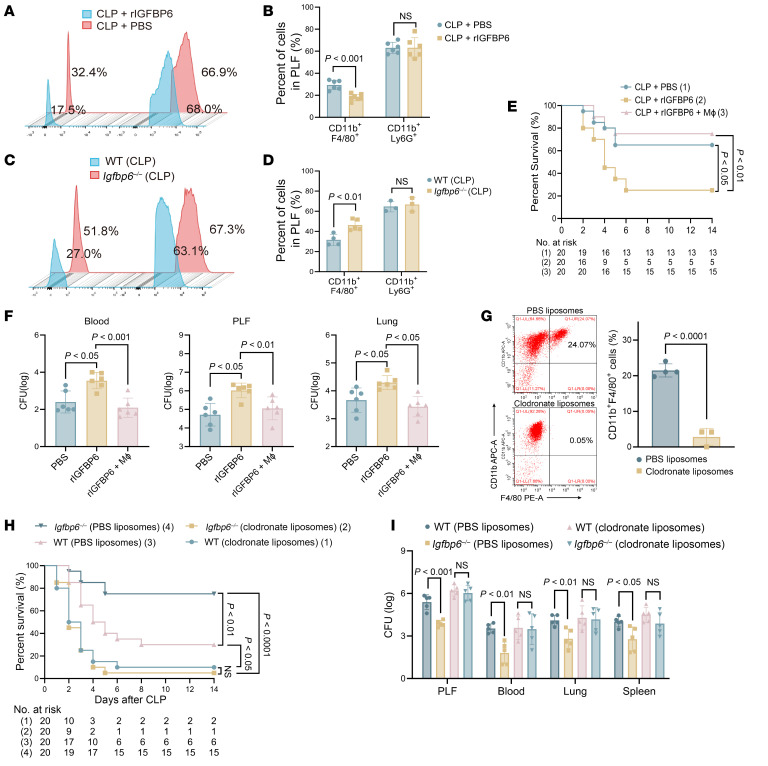
IGFBP6 impairs the chemotaxis of macrophages in sepsis. (**A** and **B**) Peritoneal macrophage (CD11b^+^F4/80^+^) and neutrophil (CD11b^+^Ly6G^+^) frequencies in rIGFBP6- or PBS-treated septic mice, analyzed by flow cytometry (*n* = 6–7/group). (**C** and **D**) Peritoneal macrophage and neutrophil frequencies in septic WT or *Igfbp6^–/–^* mice, analyzed by flow cytometry (*n* = 4–5/group). (**E**) Survival outcomes in rIGFBP6-treated mice after adoptive transfer of macrophages (1 × 10^7^/mice, *n* = 20/group). (**F**) Bacterial loads in PLF, blood, and lungs from PBS- or rIGFBP6-treated mice after macrophage adoptive transfer (*n* = 6/group). (**G**) Macrophage depletion efficacy (clodronate-liposomes versus control) assessed by CD11b^+^F4/80^+^ cell frequencies in PLF at 24 hours after CLP. Representative flow cytometry plots are shown (*n* = 3–4/group). (**H**) Mortality of WT and *Igfbp6^–/–^* septic mice in the presence or absence of macrophage depletion after CLP (*n* = 20/group). (**I**) Bacterial loads in PLF, blood, lung, and spleen of WT and *Igfbp6^–/–^* septic mice in the presence or absence of macrophage depletion after CLP (*n* = 5/group). All data are representative of 3 independent experiments. Each dot in graphs represents data from an individual mouse and an individual animal tissue. Student’s *t* test in **B**, **D**, **G**, and **I**; 1-way ANOVA in **F**; log-rank test in **E** and **H**; NS, not significant. CLP, cecal ligation and puncture; PLF, peritoneal lavage fluid.

Although accumulating evidence highlights the pivotal role of innate immune cells in sepsis progression, the mechanistic involvement of IGFBP6 in modulating these cells remains elusive. Notably, rIGFBP6 administration markedly reduced WBC count (Supplemental Figure 7A), whereas IGFBP6 deficiency increased WBC count in the PLF of septic mice (Supplemental Figure 7B). These findings were corroborated by flow cytometry data at 24 hours after CLP, in which the F4/80^+^ macrophage proportions in the PLF of rIGFBP6-treated mice were significantly reduced compared with controls, although with no concurrent alterations in Ly6G^+^ neutrophil populations (Figure 5, A and B). Immunofluorescence and flow cytometry further demonstrated diminished macrophage (F4/80^+^) recruitment to pulmonary and intestinal tissues after rIGFBP6 administration (Supplemental Figure 7, D and E). Conversely, *Igfbp6^–/–^* mice exhibited enhanced peritoneal F4/80^+^ macrophage accumulation relative to WT counterparts (Figure 5, C and D).

To determine the causality between IGFBP6-mediated macrophage deficiency and impaired host defense, adoptive transfer experiments were conducted. Macrophage reconstitution effectively rescued the IGFBP6-induced mortality (Figure 5E) and bacterial burden (Figure 5F), while macrophage ablation via clodronate liposomes (Figure 5G) abolished the survival advantage (Figure 5H) and enhanced bacterial clearance (Figure 5I) conferred by IGFBP6 deficiency. Collectively, these data establish that IGFBP6 compromises host antimicrobial defense by impairing macrophage recruitment to infection foci, thereby exacerbating systemic pathogen dissemination during sepsis.

### IGFBP6 impairs epithelial cell–derived CCL2 secretion.

**Figure 6:**
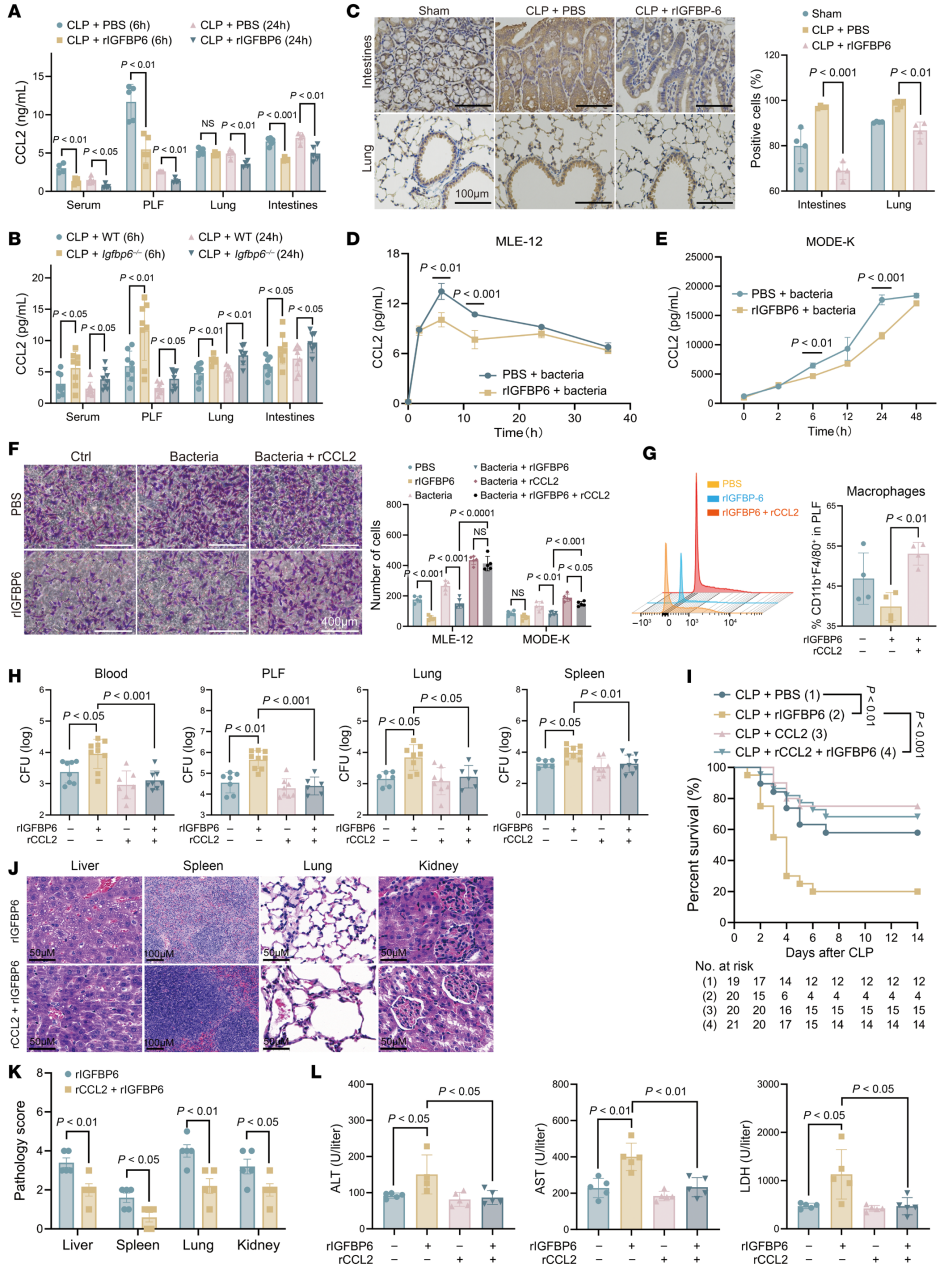
IGFBP6 inhibits the secretion of epithelial cell–derived CCL2. (**A**) CCL2 concentrations in serum, PLF, lung, and intestinal tissues from PBS- or rIGFBP6-treated septic mice assayed by ELISA (*n* = 4–6/group). (**B**) CCL2 concentrations in serum, PLF, lung, and intestinal tissues from septic WT or *Igfbp6^–/–^* mice assayed by ELISA (*n* = 8/group). (**C**) Representative IHC images and quantitative results of CCL2 expression in lung and intestinal tissues of sham control, rIGFBP6-treated, or PBS-treated septic mice (*n* = 4–5/group). Scale bar: 100 μm. (**D** and **E**) CCL2 secretion (ELISA) by MLE-12 (**D**) and MODE-K (**E**) epithelial cells stimulated with heat-killed *P*. *aeruginosa* (MOI 100) ± rIGFBP6 pretreatment (*n* = 8/group). (**F**) Macrophage chemotaxis assay: crystal violet–stained migrated cells (representative images) and quantitative analysis (*n* = 5/group). Scale bar: 400 μm. (**G**) Flow cytometry data for macrophage frequencies in PLF of rIGFBP6-treated septic mice receiving rCCL2 or PBS (*n* = 4/group). (**H**) Bacterial loads in PLF and blood from mice treated with rIGFBP6 ± rCCL2 24 hours after cecal ligation and puncture (*n* = 8–9/group). (**I**) Survival rate of septic mice treated with rIGFBP6 ± rCCL2 (half initial dose as daily maintenance for next 48 h, *n* = 19–21/group). (**J**) Representative H&E staining images of lung, liver, spleen, and kidney tissues from septic mice treated with rIGFBP6 ± rCCL2. Scale bars: 50 μm (liver, lung, and kidney), 100 μm (spleen). (**K**) Quantitative data for **J** (*n* = 5/group). (**L**) Serological biomarkers of organ injury (alanine aminotransferase, aspartate aminotransferase, LDH) in septic WT mice treated with rIGFBP6 ± rCCL2 (*n* = 4–6/group). All data are representative of 3 independent experiments. Each dot in graphs represents data from an individual biological/technical replicate. Student’s *t* test in **A**, **B**, **D**, **E**, and **K**; 1-way ANOVA in **C**, **F**–**H**, and **L**; log-rank test in **I**; NS, not significant. PLF, peritoneal lavage fluid.

Given the documented chemotactic regulatory effects of IGFBP6 in specific cell types (25), we investigated its potential chemotactic activity in macrophages. However, our findings demonstrated that IGFBP6 did not exhibit direct chemotactic effects on macrophages (Supplemental Figure 8, A and B). We were, therefore, prompted to examine other biomarkers of macrophage chemotaxis at foci of infection. Previous evidence implicated CCL2 as critical for orchestrating macrophage recruitment and bacterial clearance in sepsis (26). Notably, our analyses revealed dynamic CCL2 upregulation in PLF during early sepsis, which was substantially attenuated by rIGFBP6 administration (Figure 6A), with minimal alterations observed in other cytokines/chemokines (Supplemental Figures 9 and 10). In vivo validation confirmed pronounced IGFBP6-mediated CCL2 suppression across the PLF, serum, and lung/intestinal tissues of septic mice versus controls (Figure 6A), whereas IGFBP6 deficiency amplified CCL2 levels systemically (Figure 6B). Given the chemokine gradient dependency of immune cell trafficking (27, 28) and the epithelial enrichment of IGFBP6, we investigated tissue-specific CCL2 modulation. IHC analyses confirmed significant CCL2 inhibition in lung and intestinal epithelia of rIGFBP6-treated mice (Figure 6C). Subsequent in vitro analyses utilizing MLE-12 and MODE-K epithelial cell lines demonstrated that heat-inactivated bacterial challenge (MOI 100) induced CCL2 expression, which was suppressed by 40% with rIGFBP6 treatment (Figure 6, D and E). Importantly, this inhibitory effect was absent in macrophages, neutrophils, and lymphocytes (Supplemental Figure 8, C–E). A Transwell coculture model simulating epithelial-macrophage crosstalk under bacterial stimulation revealed that rIGFBP6 treatment significantly impaired macrophage migration (Figure 6F and Supplemental Figure 8G), indicating IGFBP6 indirectly modulates macrophage recruitment via epithelial CCL2 suppression. Functional rescue experiments demonstrated that exogenous rCCL2 administration restored F4/80^+^ macrophage infiltration in PLF (Figure 6G), enhanced bacterial clearance (Figure 6H), improved survival (Figure 6I), attenuated organ injury (Figure 6, J and K), and decreased serum biomarkers (ALT, AST, and LDH) (Figure 6L) in rIGFBP6-treated septic mice. These findings collectively establish IGFBP6 as a negative regulator of macrophage trafficking via suppression of epithelial CCL2 secretion.

### IGFBP6 downregulates CCL2 expression by inhibiting the phosphorylation and nuclear translocation of STAT1 in epithelial cells.

**Figure 7:**
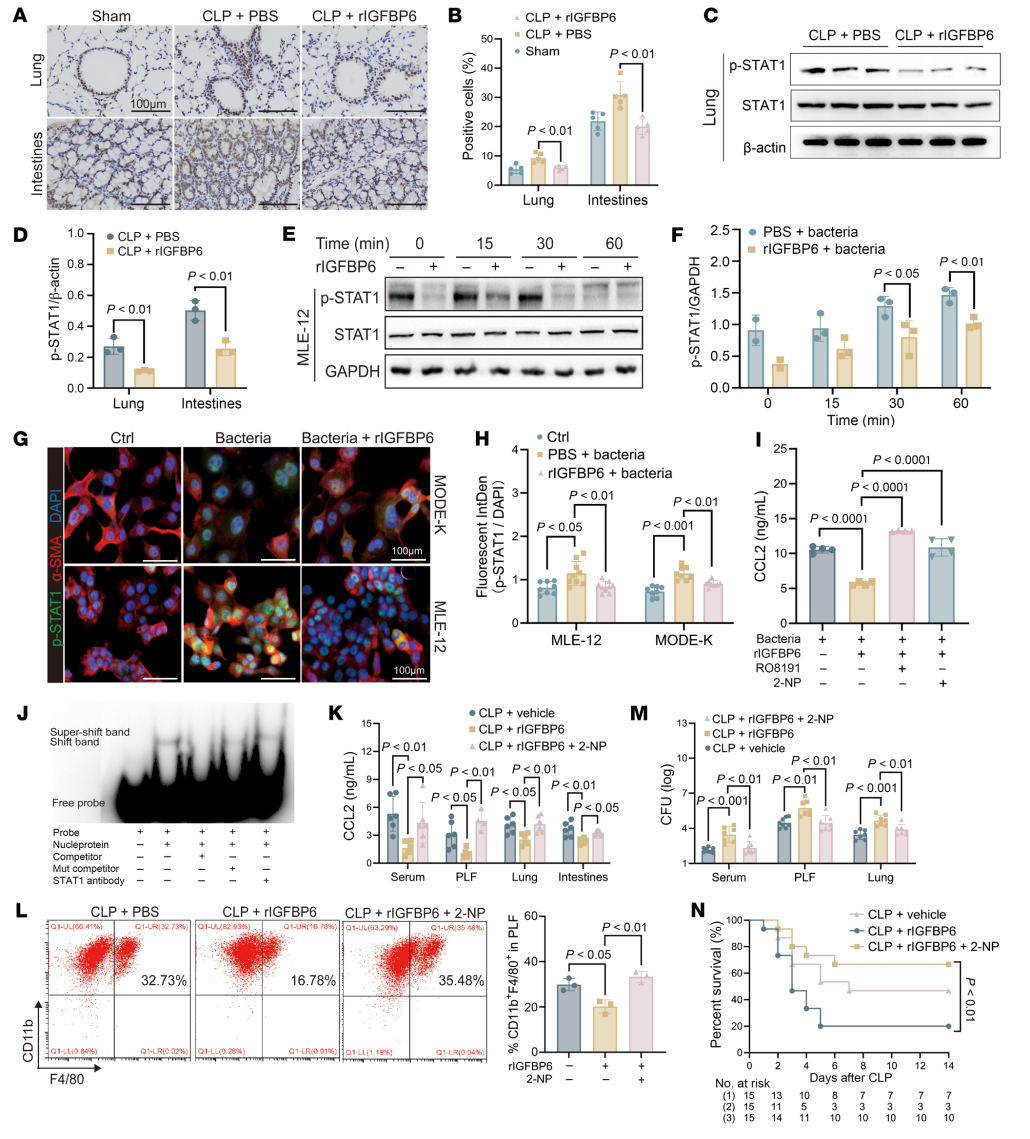
IGFBP6 downregulates CCL2 expression by inhibiting the phosphorylation and nuclear translocation of STAT1 in epithelial cells. (**A** and **B**) IHC analysis of phosphorylated STAT1 (p-STAT1) in lung and intestinal tissues from septic mice. Scale bar: 100 μm. The mean optical density of p-STAT1 was calculated (*n* = 5/group). (**C** and **D**) Western blot quantification of p-STAT1 levels in lungs of PBS- versus rIGFBP6-treated septic mice (*n* = 3/group). (**E** and **F**) Phosphorylation of STAT1 in *P*. *aeruginosa*–stimulated MLE-12 ± rIGFBP6 pretreatment at the indicated time points (MOI 100) (*n* = 3/group). (**G**) Representative confocal images and quantification of fluorescence intensity of p-STAT1 translocation in MLE-12 and MODE-K cells (p-STAT1, FITC; α-SMA, rhodamine phalloidin; nuclei, DAPI). Scale bar: 100 μm. (**H**) Quantitative data for **G** (*n* = 8–9/group). (**I**) CCL2 levels in MLE-12 cells (*n* = 4/group) pretreated with STAT1 agonist RO8191 (10 μM) or 2-NP (10 μM) for 12 hours. (**J**) EMSA performed to detect direct binding of STAT1 to the CCL2 promoter in MLE-12 in vitro. (**K**) CCL2 levels in serum, PLF, lung, and intestine of rIGFBP6 ± 2-NP–treated septic mice (*n* = 6/group). (**L**) Macrophage (CD11b^+^F4/80^+^) frequencies in PLF of rIGFBP6 ± 2-NP–treated septic mice (24 h after CLP, *n* = 3/group). (**M**) Bacterial loads in serum, PLF, and lung from rIGFBP6 ± 2-NP–treated septic mice (24 h after CLP, *n* = 8 per group). (**N**) Survival rates of PBS- or rIGFBP6-treated mice i.p. injected with or without 2-NP (*n* = 15/group). All data except **A**, **G**, and **N** are representative of 3 independent experiments. Each dot in graphs represents data from an individual biological/technical replicate. Student’s *t* test in **D** and **F**; 1-way ANOVA in **B**, **H**, **I**, **K**, **L**, and **M**; log-rank test in **N**; NS, not significant. CLP, cecal ligation and puncture; PLF, peritoneal lavage fluid.

Although STAT signaling is implicated in CCL2 regulation (29, 30), its involvement in IGFBP6-mediated CCL2 suppression has remained undefined, to our knowledge. IHC analysis revealed diminished STAT1 phosphorylation in the lung and intestinal tissues of septic mice after rIGFBP6 treatment (Figure 7, A and B), corroborated by Western blot (Figure 7, C and D, and Supplemental Figure 11A). These finding were further verified in epithelial cells, within which the phosphorylation of STAT1 in IGFBP6-treated MLE-12 and MODE-K cells was significantly reduced compared with the control group (Figure 7, E and F), with no concurrent alterations in other signaling cascades (Supplemental Figure 11, B and C). Moreover, immunofluorescence confirmed IGFBP6-mediated inhibition of STAT1 nuclear translocation (Figure 7, G and H). To establish causality, RO8191(a JAK/STAT agonist) and 2-NP (a transcription enhancer of STAT1) (31) were used to verify that IGFBP6-dependent STAT1 phosphorylation suppression directly represses CCL2 expression via JAK/STAT1 pathway inhibition (Figure 7I and Supplemental Figure 11D). Although STAT1 has been implicated in CCL2 expression, its direct transcriptional role has remained uncharacterized, to our knowledge. EMSA utilizing STAT1-specific probes targeting the CCL2 promoter (Supplemental Figure 11E and Supplemental Table 13) confirmed direct interaction between the STAT1 and CCL2 promoter (Figure 7J and Supplemental Figure 11F). In vivo functional validation through 2-NP administration in rIGFBP6-treated septic mice restored STAT1 phosphorylation in target tissues (Supplemental Figure 11, G and H), concomitant with elevated CCL2 levels in the serum, PLF, lung, and intestine (Figure 7K). Flow cytometry results revealed rescued macrophage recruitment (Figure 7L), and bacterial clearance capacity (Figure 7M) and survival rates (Figure 7N) were significantly improved. These findings delineate a mechanism wherein IGFBP6 disrupts STAT1 activation dynamics to transcriptionally suppress epithelial CCL2 production, thereby involved in sepsis-associated immune dysregulation.

### IGFBP6 inhibits STAT1/CCL2 signaling by binding to prohibitin-2.

**Figure 8:**
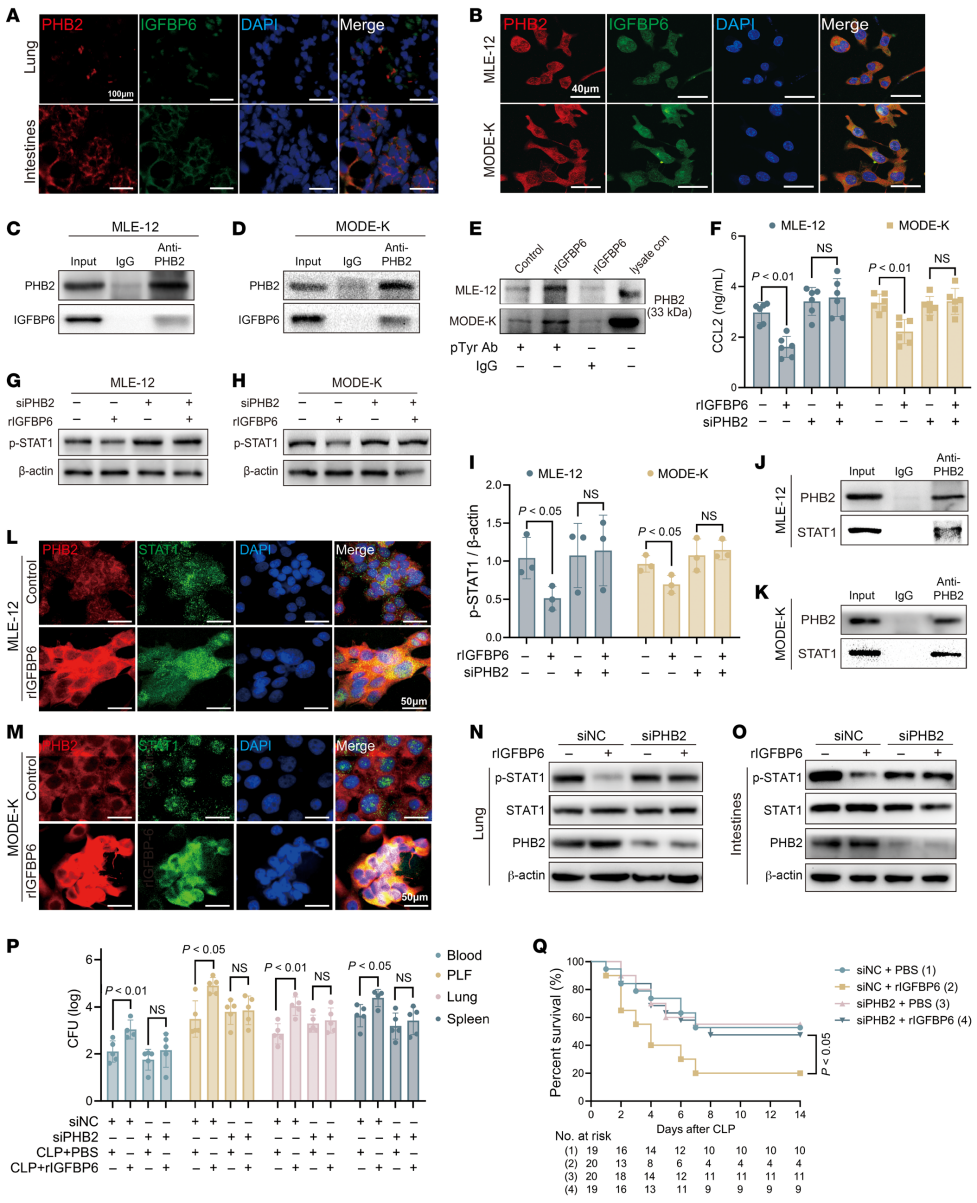
IGFBP6 inhibits STAT1 pathway via PHB2 interaction. (**A**) Representative confocal images of colocalization of IGFBP6 (FITC) and PHB2 (rhodamine phalloidin) in lung and intestinal tissues of septic mice. Nuclei counterstained with DAPI. Scale bar: 100 μm. (**B**) Representative confocal images of colocalization of IGFBP6 (FITC) and PHB2 (rhodamine phalloidin) in MLE-12 and MODE-K cells (nuclei, DAPI). Scale bar: 40 μm. (**C** and **D**) Co-IP of IGFBP6-PHB2 interaction in MLE-12 (**C**) and MODE-K (**D**). (**E**) Phosphotyrosine-specific binding assay: cell lysates from MLE-12 and MODE-K incubated with rIGFBP6 (200 ng/mL) were incubated with phosphotyrosine (pTyr Ab) or IgG control; PHB2 detected by Western blot. (**F**) CCL2 secretion in PHB2-silenced (siPHB2, 48 h) versus control (siNC) epithelial cells (MLE-12 and MODE-K) after *P*. *aeruginosa* stimulation (12 h, MOI 100; *n* = 6/group). (**H** and **I**) Phosphorylation of STAT1 in siPHB2/siNC-treated MLE-12 (**G**) and MODE-K (**H**) after *P*. *aeruginosa* challenge (30 min, MOI 100) (*n* = 3/group). (**J** and **K**) Co-IP of STAT1 with PHB2 in rIGFBP6-treated MLE-12 (**J**) and MODE-K (**K**). (**L** and **M**) Representative confocal images of the colocalization of STAT1 (FITC) and PHB2 (rhodamine phalloidin) in rIGFBP6- or PBS-treated MLE-12 (**L**) and MODE-K (**M**) (nuclei, DAPI). Scale bar: 50 μm. (**N** and **O**) After 24 hours of siNC or siPHB2 (33 μg/mouse) in vivo transfection, PHB2 level and phosphorylation of STAT1 in lung (**N**) and intestinal (**O**) tissues of PBS- and rIGFBP6-treated septic mice measured by Western blot. (**P**) Mouse PHB2 gene KD by in vivo siPHB2 transfection. Bacterial loads in peritoneal lavage fluid, blood, lung, and spleen from PBS- or rIGFBP6-treated mice assayed at 24 hours after cecal ligation and puncture (*n* = 5/group). (**Q**) After PHB2 gene KD by in vivo siPHB2 transfection, survival rates of PBS- or rIGFBP6-treated septic mice were observed for up to 2 weeks (*n* = 19–20/group). Data in **F** and **P** represent 3 independent experiments. Each dot in graphs represents data from an individual biological/technical replicate; 1-way ANOVA in **F**, **I**, and **P**; log-rank test in **Q**; NS, not significant.

To our knowledge, previous studies have not elucidated the IGF-II dependency of IGFBP6 functions; here, we confirmed that IGFBP6 suppresses CCL2 expression in an IGF-II–independent manner (Supplemental Figure 12, A and B). Prohibitin-2 (PHB2), a transmembrane protein receptor localized to the plasmalemma, mitochondrial inner membrane, and nuclear envelope, was identified as a direct interacting partner of IGFBP6 (32). Immunofluorescence analysis revealed colocalization of IGFBP6 with PHB2 in pulmonary and intestinal tissues of septic mice (Figure 8A), as well as in MLE-12 and MODE-K cell lines (Figure 8B). Direct binding was confirmed through co-IP assays (Figure 8, C and D), with subsequent studies showing IGFBP6-induced tyrosine phosphorylation of PHB2 in epithelial cells (Figure 8E). To clarify the role of PHB2 on STAT1/CCL2 regulation, 3 PHB2-targeting siRNA sequences were designed (Supplemental Table 14), among which PHB2-MUS-887 exhibited optimal knockdown efficacy (Supplemental Figure 12, C and D). PHB2 silencing abolished IGFBP6-mediated suppression of CCL2 (Figure 8F) and STAT1 regulation in both MLE-12 and MODE-K cell lines (Figure 8, G–I). Crucially, Co-IP and immunofluorescence assays demonstrated IGFBP6-dependent complex formation between PHB2 and STAT1 (Figure 8, J–M), directly implicating the PHB2/STAT1 axis in IGFBP6-mediated CCL2 regulation.

In vivo validation using TransIT-QR–delivered siPHB2 demonstrated that PHB2 knockdown abrogated rIGFBP6’s effects on STAT1 phosphorylation (Figure 8, N and O), bacterial clearance (Figure 8P), and survival outcomes (Figure 8Q) in septic mice. These data conclusively indicate that the role of IGFBP6 during sepsis is PHB2 dependent.

### IGFBP6 impairs macrophage-mediated bacterial phagocytosis and killing.

**Figure 9:**
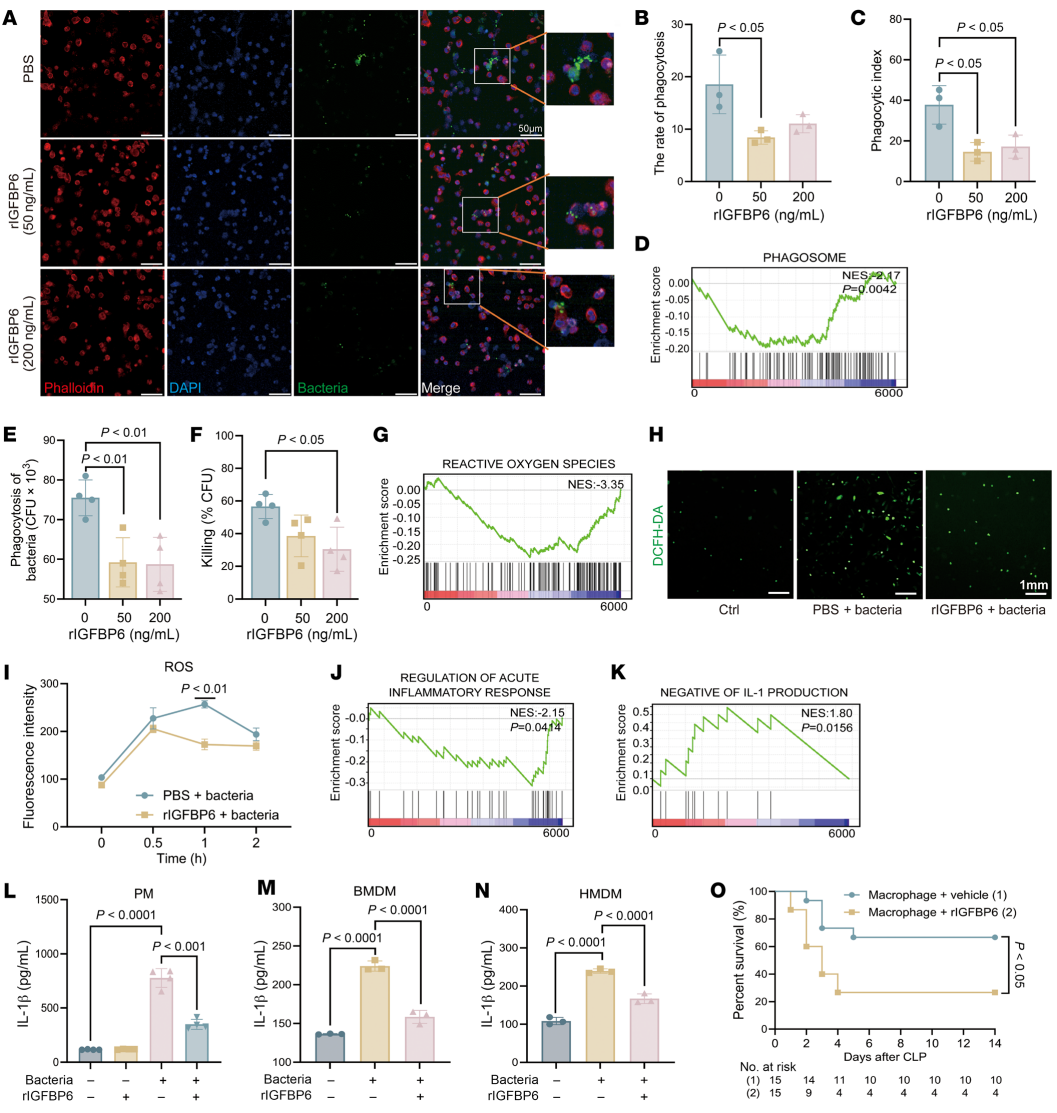
IGFBP6 impairs macrophage-mediated bacterial phagocytosis and killing. (**A**) Phagocytic activity of PMs (1 × 10^6^ cells) pretreated with rIGFBP6 (50/200 ng/mL) or PBS for 4 hours; then challenged with FITC-labeled *P*. *aeruginosa* (30 min, 37°C). Representative images from 3 independent experiments (scale bar: 50 μm). (**B** and **C**) Quantification of phagocytosis rate (**B**) and phagocytic index (**C**). (**D**) Gene set enrichment analysis (GSEA) of phagosome in the rIGFBP6 + bacteria group compared with PBS + bacteria group. (**E**) rIGFBP6-pretreated PMs (5 × 10^5^ cells) were challenged with *P*. *aeruginosa* (30 min, 37°C). Internalized bacteria were quantified by CFU counting (*n* = 4/group). (**F**) rIGFBP6-pretreated PMs (5 × 10^5^ cells) were challenged with *P*. *aeruginosa*. Bar chart shows macrophage killing rate (*n* = 7/group). (**G**) GSEA of ROS in the rIGFBP6 + bacteria group versus PBS + bacteria group. (**H**) Representative ROS fluorescence images (DCFDA staining). Scale bar: 1 mm. (**I**) Time-dependent ROS production in rIGFBP6-pretreated (200 ng/mL) PMs (1 × 10^6^ cells) challenged with heat-inactivated *P*. *aeruginosa* (*n* = 4/group). (**J** and **K**) GSEA of inflammation-related pathways in the rIGFBP6 + bacteria group versus PBS + bacteria group. (**L**–**N**) IL-1β levels in supernatants from rIGFBP6-pretreated (200 ng/mL, 4 h) PMs (5 × 10^5^ cells, **L**, *n* = 4/group), BMDMs (**M**, *n* = 3/group), and HMDMs (**N**, *n* = 3/group) after *P*. *aeruginosa* challenge (6 h). (**O**) Survival outcomes after adoptive transfer of rIGFBP6- versus PBS-treated PMs (*n* = 15/group). All data are representative of 3 independent experiments. Each dot in graphs represents an individual biological/technical replicate. Student’s *t* test in **I**; 1-way ANOVA in **B**, **C**, **E**, **F**, **I**, and **N**; log-rank test in **O**; NS, not significant. BMDMs, BM-derived macrophages; HMDMs, human monocyte–derived macrophages; PMs, peritoneal macrophages.

As primary effector cells for bacterial clearance in sepsis (33, 34), macrophages are critically regulated by IGFBP6, which was previously shown to inhibit their chemotaxis. We further investigated its impact on macrophage bactericidal activity. rIGFBP6 treatment significantly impaired the bacterial phagocytosis of macrophages (Figure 9, A–C). Gene Set Enrichment Analysis (GSEA) revealed negative enrichment of differentially expressed proteins (DEPs) in the phagosome Kyoto Encyclopedia of Genes and Genomes (KEGG) pathway after rIGFBP6 treatment (NES = –2.17; Figure 9D). Additionally, rIGFBP6 impaired bacterial killing of macrophages (Figure 9, E and F) and suppressed ROS production compared with PBS controls (Figure 9, G–I). Notably, rIGFBP6 exhibited no direct antibacterial activity (Supplemental Figure 13, A and B) or neutrophil antimicrobial functional modulation (Supplemental Figure 14, A and B).

Given the critical role of IL-1β in macrophage-driven immune activation and antiinfective responses (35, 36), rIGFBP6 treatment reduced IL-1β levels in both the PLF and serum of septic mice (Supplemental Figure 9). In bacteria-infected macrophages, GSEA highlighted suppression of acute inflammatory response and IL-1 production pathways (Figure 9, J and K), corroborated by ELISA showing reduced IL-1β levels in peritoneal macrophages, BM-derived macrophages, and human monocyte–derived macrophages (HMDMs) after rIGFBP6 treatment, while TNF-α and IL-6 remained unaffected (Figure 9, L–N, and Supplemental Figure 15, A–F). Critically, adoptive transfer of rIGFBP6-treated macrophages into septic mice exacerbated mortality compared with PBS-treated controls (Figure 9O). These findings collectively suggest that IGFBP6 exacerbates sepsis by compromising macrophage-mediated antimicrobial activity and inflammatory activation.

### IGFBP6 impairs macrophage antibacterial functions through Akt pathway inhibition.

**Figure 10:**
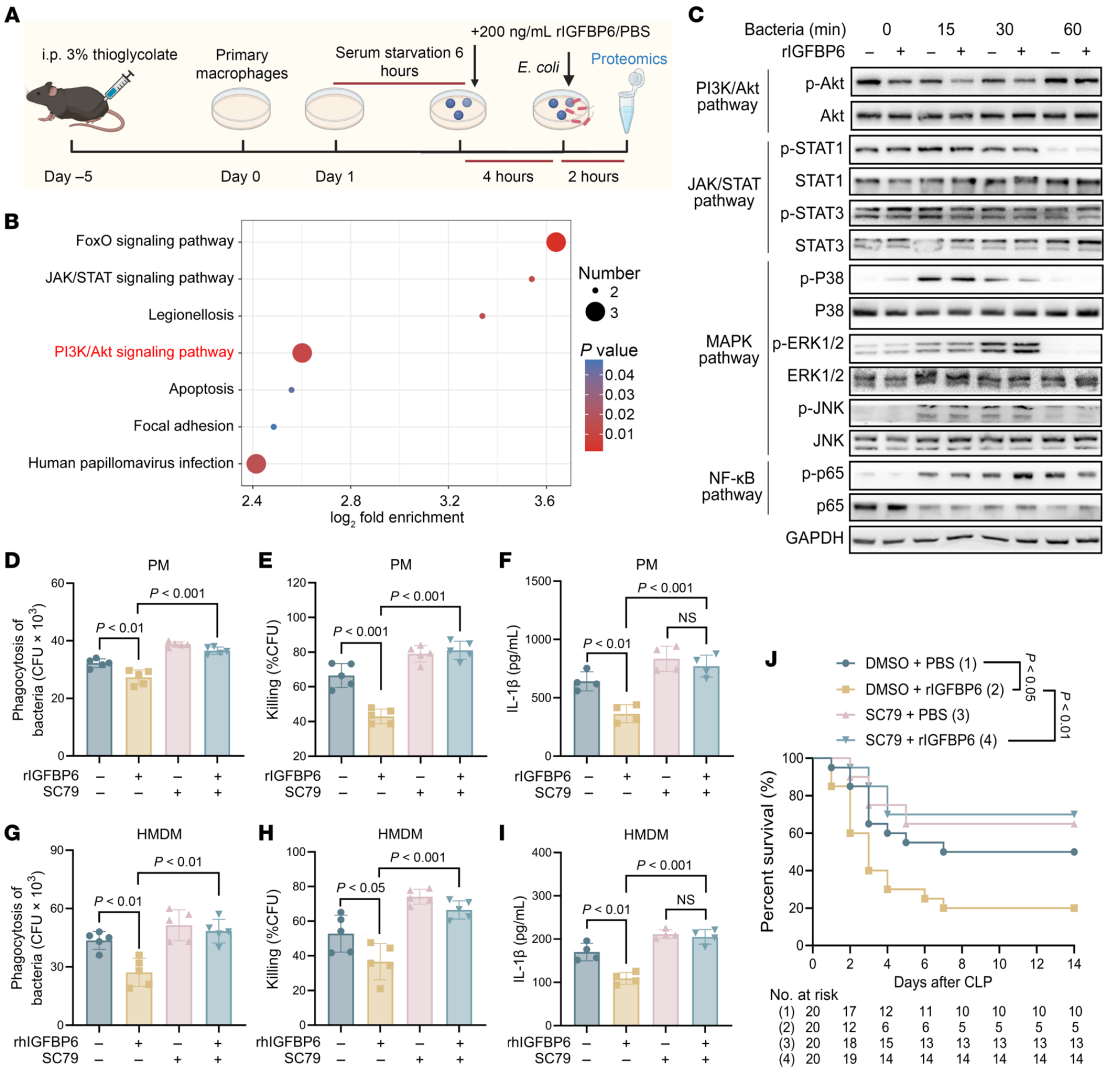
IGFBP6 impairs macrophage antibacterial functions via Akt pathway inhibition. (**A**) Experimental workflow for proteomic analysis of rIGFBP6- versus PBS-treated macrophages. (**B**) KEGG pathway analysis of DEPs between rIGFBP6-treated versus PBS-treated macrophages, with PI3K-Akt signaling highlighted (red). (**C**) Western blot analysis of PI3K/Akt, JAK/STAT, MAPK, and NF-κB pathway activation in PMs treated with rIGFBP6 (200 ng/mL) ± heat-killed *P*. *aeruginosa*. Phosphorylated proteins detected using specific antibodies: p-Akt, p-STAT1, p-STAT3, p-P38MAPK, p-ERK1/2, p-JNK, and p-p65. Representative blots from triplicate experiments shown. (**D** and **E**) PMs (*n* = 5/group) pretreated with or without rIGFBP6 (200 ng/mL, for 3 h), followed by Akt agonist SC79 incubation (25 μM, for 1 h), were assessed for phagocytosis (**D**) and killing (**E**) of *P*. *aeruginosa*. (**F**) IL-1β secretion determined in PMs pretreated with or without rIGFBP6 (200 ng/mL, for 3 h), followed by SC79 incubation (25 μM, for 1 h) and *P*. *aeruginosa* challenge (6 h, *n* = 4/group). (**G**–**I**) SC79-mediated recovery of antimicrobial functions in HMDMs (from 5 healthy volunteers, treated with rhIGFBP6 (200 ng/mL, for 3 h) ± SC79 (25 μM), followed by *P*. *aeruginosa* challenge). Phagocytosis (**G**) and killing (**H**) of *P*. *aeruginosa*; IL-1β secretion (**I**; *n* = 5/group). (**J**) Survival analysis of CLP-induced septic mice treated with SC79 (10 mg/kg) ± rIGFBP6 (*n* = 20/group). All data are representative of 3 independent experiments. Dots indicate biological replicates (culture wells); 1-way ANOVA in **D**–**I**; log-rank test in **J**; NS, not significant. CLP, cecal ligation and puncture; HMDMs, human monocyte–derived macrophages; PMs, peritoneal macrophages.

Proteomic analysis identified PI3K/Akt signaling as the main pathway enriched in IGFBP6-treated macrophages (Figure 10, A and B). To further characterize IGFBP6-mediated signaling, we assessed Akt phosphorylation after heat-killed *P*. *aeruginosa* challenge. rIGFBP6 specifically inhibited Akt phosphorylation without affecting NF-κB p65, MAPK, or JAK/STAT pathway activation (Figure 10C). Using SC79, a specific Akt agonist (37), at concentrations optimized through MTT cytotoxicity assays (Supplemental Figure 16, A and B), we demonstrated that Akt activation rescued IGFBP6-mediated impairment of bacterial phagocytosis, killing, and IL-1β production in macrophages (Figure 10, D–F). These effects were confirmed in rhIGFBP6-treated HMDMs, where SC79 restored bacterial clearance (Figure 10, G and H) and IL-1β secretion (Figure 10I). Importantly, SC79 treatment demonstrated therapeutic efficacy by improving survival outcomes in IGFBP6-treated septic mice (Figure 10J).

## Discussion

Sepsis-associated multiple organ dysfunction stems primarily from dysregulated host antiinfective responses, involving both exogenous pathogens and endogenous immune imbalances. Seminal advances in host-pathogen interaction research have established resistance (pathogen control and elimination) and disease tolerance (tissue damage control) as complementary, yet distinct, pillars of host defense (38–40). The interplay between microbial virulence factors and compromised immune regulation determines clinical trajectories ranging from disease progression to recovery, with poorly characterized immune modulators playing pivotal roles. Our study identifies IGFBP6 as a critical regulator that simultaneously undermines both resistance and disease tolerance, thereby exacerbating sepsis severity.

Our study establishes IGFBP6 as a therapeutic target in sepsis through 4 key findings: (a) Elevated circulating IGFBP6 levels in adult and pediatric patients with sepsis correlate with disease severity compared with healthy and non-septic infection controls. (b) IGFBP6 exacerbates sepsis by impairing macrophage chemotaxis and antimicrobial functions. (c) PHB2/STAT1/CCL2 axis modulation reverses IGFBP6-mediated sepsis pathology. (d) Akt pathway inhibition constitutes the principal mechanism underlying IGFBP6-induced impaired antimicrobial function of macrophages.

Crucially, these mechanistic insights map onto the resistance-tolerance framework: (a) IGFBP6-mediated suppression of macrophage chemotaxis and bactericidal activity directly reduces host resistance, leading to elevated bacterial load. (b) Independent of pathogen burden, IGFBP6 aggravates multitissue damage, indicating impaired disease tolerance. Thus, high IGFBP6 expression clinically predicts worse outcomes by reflecting combined defects in both arms of host defense. These findings position IGFBP6 as both a prognostic biomarker and therapeutic candidate for restoring immune homeostasis in sepsis.

Although IGFBP6’s roles in metabolic disorders and oncogenesis are documented, its immunoregulatory functions remain sparse. Emerging evidence from rheumatoid arthritis (10), heat-stressed DCs (41), and cystic fibrosis models (42) suggests context-dependent immune modulation, though IGFBP6’s sepsis-specific mechanisms were previously uncharacterized, to our knowledge.

Impaired pathogen clearance caused by dysregulated immunity drives sepsis mortality (43). Macrophages, central to leukocyte-mediated bacterial eradication (44–46), require precise chemotactic regulation mediated by CCL2/MCP-1 — a key macrophage chemoattractant influencing maturation, mobilization, and inflammatory recruitment (47). Our work demonstrates IGFBP6 disrupts macrophage chemotaxis via epithelial CCL2 suppression, exacerbating pathogen accumulation. This defect in bacterial clearance exemplifies compromised host resistance (1). CCL2 supplementation or IGFBP6 deficiency effectively counteracts these pathological effects, aligning with prior sepsis studies (34, 48–50). Although the literature suggests a dual role for CCL2/MCP1 (proinflammatory and cardiac injury), we have focused on the chemotactic effect of CCL2 on macrophages, which is crucial for early bacterial clearance in sepsis.

Contrary to the classical IGFBP6 dogma that defines it as an IGF2 antagonist (51, 52), mutational analyses by Fu et al. demonstrate IGFBP6’s IGF2-independent biological activity—a notion we verified in septic mice by administering either recombinant IGF2 protein (rIGF2) or an anti-IGF2 neutralizing antibody. Although IGFBP6-mediated regulation of CCL2 is independent of IGF2, the potential involvement of IGF2-dependent pathways in IGFBP6’s modulation of sepsis progression remains an open question requiring systematic investigation.

Mechanistically, we identified PHB2 as a critical mediator of IGFBP6’s transcriptional regulation. Although PHB2 typically functions as a chromatin-remodeling corepressor (53–55), we demonstrated its direct role in suppressing STAT1 phosphorylation and nuclear translocation, thereby inhibiting CCL2 transcription. EMSA confirmed STAT1’s direct binding to the CCL2 promoter, with 2-NP treatment reversing this suppression through STAT1 activation.

Our study provides initial insights into IGFBP6 and sepsis, but there are still some limitations: (a) Although IGFBP6 knockout models show therapeutic potential, the clinical translatability of genetic ablation is limited, and specific neutralizing antibodies or inhibitors targeting IGFBP6 remain undeveloped. Thus, development of specific neutralizing agents remains an urgent priority. (b) TLR2/4-mediated IGFBP6 regulation represents an unexplored mechanistic axis for sepsis progression worthy of further exploration. (c) The IGFBP6-Akt network in septic macrophages needs further elucidation. (d) Future studies employing tissue-specific or temporal IGFBP6 modulation could further dissect how resistance and tolerance mechanisms are differentially regulated during sepsis progression.

Currently, the clinical management of sepsis is still limited to supportive interventions, and definitive treatment strategies remain unclear. Considering that IGFBP6 plays a role in exacerbating immune damage during the pathological process of sepsis, inhibiting IGFBP6 expression is expected to be a potential approach to alleviate symptoms. By simultaneously enhancing resistance (improved bacterial clearance) and restoring tolerance (reduced tissue damage), IGFBP6-targeted therapies may offer a dual-benefit approach. As a biomarker of the disease, high levels of IGFBP6 indicate the risk of sepsis and dire predictions for survival. We have presented an extensive study focused upon IGFBP6 as a direct indicator of sepsis diagnosis and progression, which is also fortuitous as a mechanism for alleviation and survival.

## Methods

### Sex as a biological variable.

Because sex was not considered a biological variable or the focus of this investigation, both sexes were proportionally balanced in both the human clinical cohort and the animal models to enhance the overall generalizability of the findings across the population.

### Statistics.

All analyses were done using GraphPad Prism 10.1.2. Data are presented as mean ± SEM. Statistical significance (*P* < 0.05) was determined by unpaired *t* test (2-tailed, 2 groups) and 1-way ANOVA followed by Tukey’s post hoc test (more than 2 groups). For survival studies, Kaplan-Meier analyses were performed, followed by the log-rank test. Identified outliers were excluded. Statistical analysis was performed in all the required experiments.

### Study approval.

This study involving human participants was reviewed and approved by the Clinical Research Ethics Committee of Chongqing Medical University (No. 301 of Ethical Review 2022). No informed consent was needed because the study was noninterventional and residual blood samples were obtained after routine clinical testing. Nevertheless, non-opposition documentation was obtained from all participants or their legally authorized representatives, and the study was conducted in accordance with the Declaration of Helsinki. All animal experiments were performed in accordance with the guidelines of the IACUC of Chongqing Medical University (IACUC-CQMU-2024–0494).

### Data availability.

The raw data underlying all figures and statistical values in the main manuscript and supplemental materials are provided in the Supporting Data Values file. The full-length, uncropped original Western blots are provided in the Supplemental materials. Because of ethical restrictions related to study participant privacy (approved by the Clinical Research Ethics Committee of Chongqing Medical University), the data cannot be made publicly available in a public repository, but de-identified data (with all personal identifiers removed) can be made available from the corresponding author upon reasonable scientific request and via a signed data transfer agreement, which will further ensure compliance with privacy protection standards. All additional data related to the mouse studies, including original experimental records and supplemental analyses, are available from the corresponding author Zhixin Song upon reasonable request.

Details on the methods are provided in the Supplemental Methods.

## Author contributions

DC, ZS, and KC conceived and designed the experiments. KC, YR, XY, YH, QZ, and HW performed the experiments. KC, ZS, HT, and YL analyzed the data. KC and ZS wrote the article. DC, YY, DPM, XG, and XZ revised the article. All authors have reviewed and approved the final version of article.

## Funding support

- Chongqing Natural Science Foundation Joint Fund for Innovation and Development, Municipal Education Commission/Key Project grant CSTB2022NSCQ-LZX0045 to ZS.
- National Natural Science Foundation of China grant 82272399 to DC.
- Chongqing Young and Middle-Aged Medical Talents project to ZS.
- CQMU (Chongqing Medical University) Program for Youth Innovation in Future Medicine to DC.
- Distinguished Young Scholars of the Children’s Hospital of Chongqing Medical University to ZS.
- Clinical Research Project for the Summit Program of Children’s Hospital of Chongqing Medical University grant CHCMU-2024-XKDF-1002 to DC.
- National Natural Science Foundation of China grant 82302609 to XG.

## Supplementary Material

Supplemental data

Unedited blot and gel images

Supporting data values

## Version 1 09/02/2025

In-Press Preview

## Version 2 11/03/2025

Electronic publication
