# Supplementary material for: IGFBP6 orchestrates antiinfective immune collapse in murine sepsis via prohibitin-2–mediated immunosuppression
Source: J Clin Invest. 2025 Sep 2;135(21):e184721. doi: 10.1172/JCI184721 (PMC12578393; doi:10.1172/JCI184721)
Supplement: Supplemental data [file jci-135-184721-s342.pdf]

IGFBP6 orchestrates anti-infective immune collapse in murine sepsis via prohibitin-2-mediated immunosuppression

Kai Chen[1,2], Ying Hu[1,2], Xiaoyan Yu[1], Hong Tang[3], Yanting Ruan[1], Yue Li[4], Xun Gao[5], Qing Zhao[6], Hong Wang[2], Xuemei Zhang[2], David Paul Molloy[7], Yibing Yin[2], Dapeng Chen[1]*, Zhixin Song[1]*

Expanded materials and methods

Study Population

Participants in this study included both males and females. For adult cohort, the discovery cohort comprised 91 patients (age ≥ 18 years) admitted to the ICU of the Second Affiliated Hospital of Chongqing Medical University (Chongqing, China). The validation cohort comprised 163 patients (age ≥ 18 years) admitted to the ICU of the First Affiliated Hospital of Chongqing Medical University (Chongqing, China). All participants met the diagnostic Sepsis-3 criteria, defined as life-threatening organ dysfunction caused by a dysregulated host response to infection, with a Sequential [Sepsis-related] Organ Failure Assessment (SOFA) score ≥2, Septic shock was further characterized by persistent hypotension requiring vasopressors to maintain mean arterial pressure (MAP) ≥65 mmHg and serum lactate levels >2 mmol/L (18 mg/dL) despite adequate fluid resuscitation (1). Demographic and clinical data were systematically collected, including age, sex, comorbidities, SOFA scores at admission, infection etiology, microbiological findings (pathogen identification and infection source), therapeutic interventions (mechanical ventilation, vasopressor use, hydrocortisone administration, renal replacement therapy), ICU length of stay, and 28-day mortality. Exclusion

criteria encompassed pregnancy, breastfeeding, active malignancy, prior organ transplantation, HIV infection, autoimmune disorders, or immunosuppressive therapy use (2, 3). Peripheral blood samples were collected at ICU admission and separated by centrifugation (1500g×10 min, 4°C), then stored at -80°C until analysis. Age- and sex-matched non-septic infected patients and healthy volunteers from the same hospitals served as control groups.

For pediatric cohorts, serum samples from 61 patients (<18 years) in the discovery cohort were obtained from Kunming Children's Hospital (Yunnan, China), while the validation cohort (n = 145) was derived from the Children's Hospital of Chongqing Medical University (Chongqing, China). Pediatric sepsis was diagnosed according to the 2024 International Consensus Criteria for Pediatric Sepsis, requiring confirmed infection combined with a Phoenix Sepsis Score (PSS) ≥2 (4). Neonates and preterm infants (gestational age < 37 weeks) hospitalized during the perinatal period were excluded (4). Control groups comprised age-matched non-septic infected children and healthy volunteers.

Animals

Male and female wild type (WT) C57BL/6N mice (6-8 weeks old) and IGFBP6-deficient (*Igfbp6*$^{-/-}$) mice on a C57BL/6N genetic background were procured from the Laboratory Animal Center of Chongqing Medical University (LAC-CQMU) and Cyagen Biotechnology Co., Ltd. (Suzhou, China), respectively. All animals were raised under Specific Pathogen Free (SPF) laboratory of LAC-CQMU. The deletion of *Igfbp6* was verified by polymerase chain reaction (PCR)-based genotyping of tail-derived genomic DNA using two primer sets (TSE041, TSINGKE): Primer set 1: 5'-CTGGACTCTCTGGAAGGAGTG-3' (forward), 5'-ACCAGCCCTTCACTTGTAGCC-3' (reverse), Primers 2: 5'-

CTGGACTCTCTGGAAGGAGTG-3’ (forward), 5’-ACTTCACGTTCCACTCAAGGC-3’ (reverse). The thermal cycler settings of *Igfbp6* were 3 minutes at 94°C; 35 cycles of 30 seconds at 94°C, 35 seconds at 60°C, and 35 seconds at 72°C; and extension for 5 minutes at 72°C. PCR products were resolved by 2% agarose gel electrophoresis for genotype confirmation.

Sepsis Model

CLP-induced polymicrobial sepsis model was established as described previously (5-7). Briefly, age-matched male and female C57BL/6N mice were anesthetized intraperitoneally (i.p.) with xylazine (4.5 mg/kg) and ketamine (90 mg/kg). A 1 cm midline laparotomy was performed to expose the cecum, which was then ligatured at its external third, and punctured through with a 21-gauge needle (severe CLP, resulting in 0 to 20% survival) or with a 24-gauge needle (resulting in 50 to 60% survival). Sham-operated (control) animals underwent identical laparotomy without cecal ligation or puncture. The cecum was then returned to the abdominal cavity, and the incision was closed in two layers using 5-0 absorbable sutures. Postoperative care included subcutaneous administration of 1 mL prewarmed Ringer’s lactate solution containing buprenorphine (0.05 mg/kg) for fluid resuscitation and analgesia. Mice were maintained in a temperature-controlled incubator and monitored every 6 hours for the first 48 hours, followed by 8-hour intervals until study completion. To administer antibiotic therapy, CLP models were given 5 mg/mL meropenem in saline at a dose of 25 mg/kg through intraperitoneal injection immediately post-surgery, and then once a day for 7 consecutive days. Mice exhibiting severe morbidity (e.g., non-responsiveness, labored breathing, or >20% weight loss within 24 h) were euthanized via cervical dislocation under anesthesia. Survival was tracked for 14 consecutive days.

Treatment of mice with rIGFBP6, rCCL2 and agonist

Recombinant murine IGFBP6 was administered i.p. at 2.5, 10 and 50 μg/kg at 1h post-CLP, with PBS serving as the vehicle control. A dose of 25 μg/kg rIGFBP6 was used in all experiments except the survival experiments. For survival experiments, we maintained therapeutic relevance by administering half the initial dose of rIGFBP6 at 24 and 48 hours post-CLP. Recombinant murine CCL2 (rCCL2) was delivered i.p. at 25 μg/kg at 1h post-CLP, with PBS administered to controls. For *in vivo* activation of STAT1, the agonist 2-NP was dissolved in a dimethyl sulfoxide (DMSO) solution (10% DMSO + 90% corn oil) and administrated i.p. immediately following CLP.

Single-bacterial sepsis model

A *S. aureus* - induced peritonitis-related sepsis model was established by i.p. injection of 3×10^8 CFU into male C57BL/6N mice. For the *P. aeruginosa* - induced sepsis, 5×10^7 CFU were injected i.p. into male C57BL/6N mice. Survival was monitored twice daily for 14 days post-infection.

Quantification of bacterial loads

Peritoneal lavage fluids (PLF) were obtained through infusion of 5 mL PBS into the murine abdominal cavity using a sterile syringe, followed by gentle aspiration. Specimens including PLF, blood, and tissue samples were aseptically collected following CLP. All biological samples underwent serial 10-fold dilution in PBS, and plated on blood agar (tryptone soya agar with 5% sheep blood) plates (Thermo Fisher Scientific), and incubated at 37°C for 18h, after which the number of colonies was counted.

Histopathology, immunohistochemistry, and immunofluorescence

Following euthanasia, mice were transcardially perfused with PBS, followed by 4% paraformaldehyde for tissue fixation. Organs were paraffin-embedded and sectioned at 5μm thickness for hematoxylin and eosin (H&E) staining. Histopathological scoring was performed using a standardized four-stage grading system to assess inflammation and tissue damage (8). For immunohistochemistry (IHC) and immunofluorescence (IF), slices were dewaxed and rehydrated, followed by microwave repair of antigens in citric acid buffers (pH 6.0). The specimens were then permeabilized with PBS containing 0.1% Triton X-100, and blocked endogenous peroxidase with 3% H_2O_2. Thereafter, sections were blocked with 5% BSA for 30 minutes at room temperature and incubated with anti-CCL2 antibodies or anti-phospho-STAT1 antibodies overnight at 4°C in a wet box. For immunohistochemistry, sections were incubated with HRP-conjugated secondary antibody and peroxidase TMB substrate kit for 1 hour at room temperature. Hematoxylin was used for nucleus counterstaining. For immunofluorescence, after blocked with 5% BSA, sections and cell slides were incubated with anti-IGFBP6, anti-F4/80, anti-α-SMA, anti-PHB2, anti-STAT1 overnight at 4°C and then incubated with CY3 or FITC conjugated secondary antibody. DAPI was used for nuclear staining. Sections were observed and photographed with an inverted fluorescence microscope (Olympus IX71). Fluorescence integrated density was analyzed using Image J software (version 5.0, NIH).

Epithelial cell culture and treatment

The mouse lung epithelial cell line (MLE-12) and mouse small intestinal epithelial cell line (MODE-K) were maintained in Dulbecco's Modified Eagle Medium (DMEM, 11995065, Gibco) supplemented with 10% fetal bovine serum (FBS, C2910-0500, VivaCell) and 1% penicillin/streptomycin (Gibco BRL) at 37°C under 5% CO_2. For experiments, Cells were

seeded in six-well plates at 1×10^5/mL and cultured in serum-free medium for 12h when the cells reached 80% confluence. After starvation, cells were pretreated with rIGFBP6 (200 ng/mL) for 6 hours, followed by stimulation with inactivated *P. aeruginosa* (MOI = 100).

Characterization of leukocytes

Peritoneal cell suspensions were pelleted and resuspended in PBS. Cytospin slides were prepared and stained with a Wright-Giemsa stain. Cells were washed in FACS buffer (1% BSA in PBS), stained with fluorophore-conjugated antibodies against CD11b, Ly6G, and F4/80, and analyzed using a FACScan flow cytometer (Becton Dickinson, NJ, USA). Data from $\geq 10^5$ events per sample were processed with FC Express software. Gating strategies for macrophage and neutrophil populations are detailed in Supplementary Figure 7B.

Serum biochemistry

Blood was collected in tubes with heparin after cardiac puncture and centrifuged to separate serum. Serum alanine aminotransferase (ALT), aspartate aminotransferase (AST), and lactate dehydrogenase (LDH) were determined with commercial kits (Sigma-Aldrich, St. Louis, MO), using a Hitachi analyzer (Boehringer Mannheim; Germany) according to the manufacturers' instructions.

***In vivo* depletion of macrophages**

Clodronate-encapsulated liposomes were delivered i.p. (200 μL, 5 mg/mL), to deplete macrophages at 48 hours before CLP-induced sepsis. PBS-encapsulated liposomes were delivered in a similar way as a control.

Isolation and culture of primary murine macrophages and neutrophils

Primary peritoneal macrophages were isolated from the peritoneal exudates of C57BL/6N mice

that were pretreated with 3% thioglycolate i.p. for 5 days. The peritoneal exudate cells were harvested via cold PBS lavage, resuspended in DMEM supplemented with 10% FBS, and adjusted to 1×10^6 cells/mL. After 2-hour incubation at 37°C under 5% CO_2, non-adherent cells were removed by PBS washing. Adherent cells (>90% F4/80$^+$ by FCM) were maintained in complete DMEM (6, 9). Bone marrow-derived macrophages (BMDMs) were generated by flushing femurs and tibias with DMEM. Cells were cultured for 7 days in DMEM containing 10% FBS and 50 ng/mL recombinant murine M-CSF, yielding >90% F4/80$^+$ macrophages (10). Bone marrow neutrophils were isolated using a murine neutrophil isolation kit. Purity (>90% CD11b$^+$Ly6G$^+$) was confirmed via FCM as previously described (11).

Macrophage migration assay

Cell migration assays were performed using a 24-well transwell® apparatus with a 0.4 μm pore size (Corning Inc., NY, USA). Approximately 6×10^4 peritoneal macrophage with a volume of 200 μL were loaded into the upper chambers of the trans-well plates. $1\text{x}10^5$ MLE-12 or MODE-K with a volume of 600μL conditioned media was collected and added to the lower chambers. After 6 hours of rIGFBP6 pre-stimulation, heat-inactivated bacteria were added to the upper chambers of the trans-well plates. After 12 hours incubation, the cells were fixed with pre-cooled methanol and stained with crystal violet. Non-migrating cells on the upper surface of the membrane were gently removed. The numbers of migrated cells were counted in five randomly chosen fields per insert using Image J software.

Adoptive transfer of macrophages

Peritoneal macrophages were isolated from C57BL/6N WT or *Igfbp6*$^{-/-}$ mice (age 6-8 weeks, male/female 1:1) as described above and treated with 200 ng/mL of rIGFBP6 or PBS for 24

hours to activate the macrophages. The cell viability was calculated by 0.4% Trypan Blue staining solution, and the cell viability was required to be no less than 90%. Cell transfer was performed as previously described (12). In short, 6 hours after CLP surgery, the qualified macrophages treated with rIGFBP6 or PBS were collected, washed twice with PBS, the cell density was adjusted to 1×10^8/mL, and 1×10^7 (100 uL) macrophages were injected intravenously into the mice.

Culture of human monocyte-derived macrophages (HMDM)

Blood was obtained from healthy individuals from the in-house blood donation unit. PBMCs were isolated by density centrifugation using Ficoll-Paque™ PREMIUM 1.084. Isolated PBMCs were incubated with CD14-binding MACS beads for 15 min. Cells were magnetically sorted by positive selection using LS columns (130-042-401; Miltenyi Biotec). Isolated $CD14^+$ cells were differentiated into macrophages using 50 ng/mL M-CSF in RPMI 1640 medium supplemented with 5% heat-inactivated FBS and 2 mM GlutaMAX. Cells were seeded in 24-well plates at 5×10^5 for bacterial infection or cell supernatant acquisition and differentiated into hMDM for 7 d prior to use in experiments. In selected experiments, cells were treated with or without 25 μM of SC79 in the presence or absence of recombinant human IGFBP6 Protein (200 ng/mL) for 4 hours before stimulation with *P. aeruginosa*.

Bacterial phagocytosis assays

Fluorescein isothiocyanate (FITC, Invitrogen)-labeled heat-killed *P. aeruginosa* was first prepared by incubation of *P. aeruginosa* with 0.5 mg/mL FITC for 30 minutes at 37°C. Macrophages (1×10^6 cells) or neutrophils (1×10^6 cells) were incubated with FITC-labeled *P. aeruginosa* at a multiplicity of infection (MOI) of 100 for 30 minutes at 37°C to induce

phagocytosis. Cells were then washed twice with cold PBS, cytoskeleton was stained with TRITC Phalloidin and cell nuclei were stained with 4',6-diamidino-2-phenylindole, followed by visualization using confocal laser scanning microscopy (TCS SP8, Leica).

Bacterial killing assays

Intracellular bacterial killing was determined by incubation of macrophages with live *P. aeruginosa* in the presence or absence of rIGFBP6. Briefly, murine peritoneal macrophages (5×10^5 cells) were infected with live *P. aeruginosa* (MOI = 1:100) at 37°C for 30 minutes. Macrophages were then washed with buffer containing tobramycin (100 µg/mL) to remove extracellular bacteria and subsequently lysed with lysis buffer (Promega). Live intracellular bacteria were quantified by culture of lysates for determination of bacterial uptake (at t = 0 hour) and intracellular killing (at t = 2 hours). Killing rate was calculated from the percentage of colonies present at t = 2 hours as compared to t = 0 hour, as follows: 100 − [number of CFU at t = 2 hours] / [number of CFU at t = 0 hour]. For intracellular bacterial killing by neutrophils, primary murine neutrophils (1×10^6 cells) were infected with live *P. aeruginosa* at an MOI of 100 at 37°C for 30 minutes. The neutrophils were then resuspended in medium containing 100 µg/mL tobramycin to remove extracellular bacteria and subsequently lysed in PBS containing 0.1% Triton 100 for assessment of uptake (t = 0 hour). Additional samples were incubated for 1 additional hour (t = 2 hour) to assess bacterial killing as described above.

Reactive oxygen species (ROS) measurement

Peritoneal macrophages were incubated with heat-inactivated *P. aeruginosa* at an MOI of 100 in DMEM cultured at 37 ºC in 5% CO_2 for 0, 0.5, 1, 2 hours. To measure total intracellular ROS activity, macrophages were treated with the fluorogenic probe DCFH-DA (catalog S0033,

Beyotime) at 5 μM for 30 minutes at 37°C. The medium was then removed, and the cells were returned to prewarmed fresh growth medium. The emitted fluorescence was detected by a fluorescent microplate reader using 490/520 nm excitation/emission filters (Molecular Devices). ROS activity was reported as fluorescence intensity.

Proteomics and analyses

In brief, peritoneal macrophages treated with rIGFBP6 or PBS was sonicated three times on ice using a high intensity ultrasonic processor (Scientz) in lysis buffer (8 M urea, 1% protease inhibitor cocktail). The remaining debris was removed, and the supernatant was collected and the protein concentration was determined with BCA kit. For digestion, the protein solution was reduced with 5 mM dithiothreitol for 30 min at 56 °C and alkylated with 11 mM iodoacetamide for 15 min at room temperature in darkness. The protein sample was then diluted by adding 100 mM TEAB to urea concentration less than 2 M. Finally, trypsin was added for digestion. The peptides were desalted by C18 SPE column. The resulting MS/MS data were processed using MaxQuant search engine (v.1.6.15.0). Tandem mass spectra were searched against the human SwissProt database (20422 entries) concatenated with reverse decoy database. FDR was adjusted to < 1%. This bioinformatics Methods contain GO analysis, COG analysis, pathway functional annotation analysis, and time series analysis. Based on the quantitative results, the differentially abundant proteins between groups were identified, and function enrichment analysis, protein-protein interaction (PPI) analysis, and subcellular localization analysis of the differentially abundant proteins were performed.

Electrophoretic Mobility Shift Assay (EMSA)

The direct interaction between STAT1 and CCL2 promoter was examined using an EMSA/Gel

Shift Kit in accordance with the manufacturer's instructions. Cell nuclear extracts were isolated and incubated with biotin-labeled probes containing the CCL2 consensus sequence. Next, the specific anti-STAT1 antibody was added to the mixture of nuclear extracts and DNA probes. The DNA-protein complexes were transferred onto a nylon binding membrane and detected using a streptavidin-horseradish peroxidase conjugate enhanced chemiluminescence (ECL) detection system. The primer sequence of the CCL2 promoter is shown in Supplemental Table 13.

Western blot

Cells were lysed with RIPA buffer (Beyotime) containing PMSF (Beyotime) and phosphatase inhibitors (Beyotime). The proteins in lysates were denaturated with SDS-PAGE loading buffer (Beyotime) containing β-mercaptoethanol (β-ME) and heated at 100 °C for 10 minutes. Proteins were separated on SDS-PAGE gels and transferred to polyvinylidene difluoride (PVDF) (Merk-Millipore, Billerica, USA). Membranes were blocked with 5% BSA for 2 h and incubated at 4°C overnight with primary antibodies. Then, membranes were incubated with HRP conjugate secondary antibodies for 1 h and visualized using ECL chemiluminescence analysis by Image Lab 6.0 (Bio-Rad, CA, USA). The relative expression levels of the target proteins were standardized to internal controls while densitometric quantification of band intensities was calculated using ImageJ 5.0 software (NIH, MD, USA).

Co-Immunoprecipitation

Co-Immunoprecipitation (Co-IP) was performed according to the manufacturer's instructions for the Pierce™ Co-Immunoprecipitation Kit. First, the primary antibody was immobilized by coupling it to the resin for 2 hours. Then, the pretreated cell lysate was incubated with the

immobilized antibody at 4°C overnight to form immune complex. Finally, the bound protein was eluted for western blotting analysis.

3-(4, 5-dimethylthiazol-2-yl)-2, 5-diphenyltetrazolium bromide (MTT) assay

Peritoneal murine macrophages (1×10^5 cells/0.2 mL) were inoculated into a 96-well plate. Various inhibitors at serial concentrations were added to the cells. At 24 hours after incubation, MTT (50 µg; M5655, Sigma) was added to each well and incubated for 2 hours. Viable cells took up MTT and reduced it into dark blue, water-insoluble formazan by mitochondrial dehydrogenase, which reflected the normal function of mitochondria and cell viability. The cells were then lysed with DMSO to yield the color solution. The absorbance at 550 nm was measured to quantify the viable cells.

SiRNA *in vivo* transfection

Trans IT® -QR Hydrodynamic Delivery Solution was used to deliver siRNA to mice (13). Use a restraint device to secure the mouse during the injection, the total volume required was calculated according to mouse weight/10 + 0.1mL. The siRNA and Delivery Solution were mixed and injected through the tail vein at a constant speed within 4-7 seconds.

References:

1. Singer M, Deutschman CS, Seymour CW, Shankar-Hari M, Annane D, Bauer M, et al. The Third International Consensus Definitions for Sepsis and Septic Shock (Sepsis-3). *Jama.* 2016;315(8):801-10.
2. Santos I, Colaço HG, Neves-Costa A, Seixas E, Velho TR, Pedroso D, et al. CXCL5-mediated recruitment of neutrophils into the peritoneal cavity of Gdf15-deficient mice protects against abdominal sepsis. *Proc Natl Acad Sci U S A.* 2020;117(22):12281-7.
3. Mikacenic C, Price BL, Harju-Baker S, O'Mahony DS, Robinson-Cohen C, Radella F, et al. A Two-Biomarker Model Predicts Mortality in the Critically Ill with Sepsis. *Am J Respir Crit Care Med.* 2017;196(8):1004-11.
4. Schlapbach LJ, Watson RS, Sorce LR, Argent AC, Menon K, Hall MW, et al. International Consensus Criteria for Pediatric Sepsis and Septic Shock. *Jama.* 2024;331(8):665-74.
5. Rittirsch D, Huber-Lang MS, Flierl MA, and Ward PA. Immunodesign of experimental sepsis by cecal ligation and puncture. *Nat Protoc.* 2009;4(1):31-6.
6. Song Z, Zhang X, Zhang L, Xu F, Tao X, Zhang H, et al. Progranulin Plays a Central Role in Host Defense during Sepsis by Promoting Macrophage Recruitment. *Am J Respir Crit Care Med.* 2016;194(10):1219-32.
7. Bai H, Lu Q, Wu C, Xu F, Liu J, Wang K, et al. Bone morphogenetic protein 9 is a candidate prognostic biomarker and host-directed therapy target for sepsis. *Science translational medicine.* 2024;16(732):eadi3275.
8. Achouiti A, Vogl T, Urban CF, Rohm M, Hommes TJ, van Zoelen MA, et al. Myeloid-

related protein-14 contributes to protective immunity in gram-negative pneumonia derived sepsis. *PLoS Pathog.* 2012;8(10):e1002987.

9. Nascimento DC, Viacava PR, Ferreira RG, Damaceno MA, Piñeros AR, Melo PH, et al. Sepsis expands a CD39(+) plasmablast population that promotes immunosuppression via adenosine-mediated inhibition of macrophage antimicrobial activity. *Immunity.* 2021;54(9):2024-41.e8.

10. Zhao T, Zhong G, Wang Y, Cao R, Song S, Li Y, et al. Pregnane X Receptor Activation in Liver Macrophages Protects against Endotoxin-Induced Liver Injury. *Advanced science (Weinheim, Baden-Wurttemberg, Germany).* 2024;11(19):e2308771.

11. Tsuji N, Tsuji T, Yamashita T, Hayase N, Hu X, Yuen PS, et al. BAM15 treats mouse sepsis and kidney injury, linking mortality, mitochondrial DNA, tubule damage, and neutrophils. *J Clin Invest.* 2023;133(7).

12. Lu XJ, Chen J, Yu CH, Shi YH, He YQ, Zhang RC, et al. LECT2 protects mice against bacterial sepsis by activating macrophages via the CD209a receptor. *J Exp Med.* 2013;210(1):5-13.

13. Lewis DL, and Wolff JA. Systemic siRNA delivery via hydrodynamic intravascular injection. *Advanced drug delivery reviews.* 2007;59(2-3):115-23.

Supplemental Table 1. Clinical characteristics of sepsis patients and healthy volunteers in adult discovery cohort.

Characteristics	Healthy controls (*n* = 48)	Infection controls (*n* = 42)	All sepsis patients (*n* = 91)	Sepsis survivors patients (*n* = 75)	Sepsis non-survivors patients (*n* = 16)	*P* value (Survivors VS Non-survivors)
Male/Female	28/20	32/10	48/43	38/37	10/6	0.0812
Age, years	51(35-61)	65(56-78)	59(47-70)	62(47-72)	60(51-67)	0.7040
WBC, 10^9/L	NA	11.3(7.7-13.8)	11.9(6.0-14.4)	14.5(6.9-17.2)	11.5(6.2-15.8)	0.2590
PLT, 10^9/L	NA	190(113-256)	158(80-254)	118(81-248)	159(54-255)	0.9472
CRP, mg/L	NA	48(11-75)	101(34-180)	88(30-156)	113(55-189)	0.1275
PCT, ng/mL	NA	17.1(0.4-24.8)	38.9(1.9-49.5)	31.4(0.5-59.4)	70.1(3.6-105.1)	0.2348
Infection site, no. of patients						
Respiratory	NA	42	40	26	14	-
Abdominal	NA	0	5	4	1	-
Vascular	NA	0	24	22	2	-
Urinary	NA	0	18	16	2	-
Other	NA	0	21	17	4	-
Isolates, no. of patients						
Gram positive	NA	12	26	22	4	-
Gram negative	NA	24	42	36	6	-
Fungus	NA	2	6	4	2	-
Miscellaneous	NA	2	10	6	4	-
Other	NA	2	7	7	0	-
Sepsis shock	NA	-	26	16	10	**0.0103**
SOFA score	NA	-	6(4-8)	8(4-8)	9(8-10)	**<0.0001**
ICU stay	NA	10(8-13)	27(10-48)	25(10-48)	19(9-26)	0.2511

Categorical variables are expressed as *n* (%), and continuous variables were expressed as median (interquartile range). Comparisons between sepsis patients and sepsis shock patients, survivors and non-survivors were performed with chi-square or Fisher exact tests for qualitative variables and Mann-Whitney *U* for quantitative variables. SOFA score was measured after 24 hours of ICU stay.

Abbreviations: WBC, white blood cell; PLT, platelet; CRP, C-reactive protein; PCT, procalcitonin; SOFA, Sequential Organ Failure Assessment; ICU, intensive care unit; NA, not applicable.

Supplemental Table 2. Clinical characteristics of sepsis patients and healthy volunteers in the adult validation cohort.

Characteristics	Healthy controls (*n* = 92)	Infection controls (*n* = 116)	All sepsis patients (*n* = 163)	Sepsis survivors patients (*n* = 102)	Sepsis non-survivors patients (*n* = 61)	*P* value (Survivors VS Non-survivors)
Male/Female	49/43	73/43	86/77	56/46	30/31	0.479
Age, years	53(50-60)	65(59-79)	56(53-76)	65(55-79)	64(56-73)	0.522
WBC, 10^9/L	NA	10.7(6.3-13.8)	12.0(6.3-13.9)	11.1(6.5-12.7)	10.0(8.3-12.1)	0.417
CRP, mg/L	NA	43.5(5.0-69.2)	138(73.6-200.0)	127.2(68-200.0)	146.4(109.3-200.0)	0.104
PCT, ng/mL	NA	9.9(0.1-9.1)	39.6(2.8-54.7)	51.4(2.7-56.6)	62.4(4.9-98.1)	0.190
PLT, 10^9/L	NA	223(177-278)	120(60-165)	133(73-207)	95(43-144)	0.200
Infection site, no. of patients						
Respiratory	NA	85	67	45	22	-
Abdominal	NA	9	45	28	17	-
Vascular	NA	0	19	6	13	-
Urinary	NA	6	14	10	4	-
Other	NA	16	18	13	5	-
Isolates, no. of patients						
Gram positive	NA	36	47	32	15	-
Gram negative	NA	52	67	44	23	-
Fungus	NA	9	15	9	6	-
Miscellaneous	NA	12	28	12	16	-
Other	NA	7	6	5	1	-
Sepsis shock	NA	-	61	30	31	**0.0001**
APACHE II score	NA	8(4-12)	18(13-23)	14(9-18)	21(17-27)	**0.0025**
SOFA score	NA	-	8(5-11)	7(4-9)	11(9-13)	**0.0014**
ICU stay	NA	12(5-19)	10(3-15)	11(3-15)	8(1-11)	0.469

Categorical variables are expressed as *n* (%), and continuous variables were expressed as median (interquartile range). Comparisons between sepsis patients and sepsis shock patients, survivors and non-survivors were performed with chi-square or Fisher exact tests for qualitative variables and Mann-Whitney *U* for quantitative variables. APACHE II score and SOFA score were measured after 24 hours of ICU stay.

Abbreviations: WBC, white blood cell; PLT, platelet; CRP, C-reactive protein; PCT,

procalcitonin; APACHE II, acute physiology and chronic health evaluation II; SOFA, Sequential Organ Failure Assessment; ICU, intensive care unit; NA, not applicable.

Supplemental Table 3. Clinical characteristics of sepsis patients and healthy volunteers in the pediatric discovery cohort.

Characteristics	Healthy controls (*n* = 53)	All sepsis patients (*n* = 61)	Sepsis survivors patients (*n* = 50)	Sepsis non-survivors patients (*n* = 11)	*P* value (Survivors VS Non-survivors)
Male/Female	30/23	30/31	23/27	7/4	0.280
Age, years	7(3-12)	3(1-4)	3(2-8)	2(1-3)	0.222
WBC, 10^9/L	NA	15.1(10.2-21.3)	14.5(10.6-18.4)	12.3(5.8-18.8)	0.204
CRP, mg/L	NA	76.7(21.9-128.4)	62.2(17.0-101.0)	98.8(31.0-179.4)	0.199
PCT, ng/mL	NA	7.1(0.3-3.7)	1.2(0.3-1.5)	30.3(3.2-49.7)	**<0.0001**
Infection site, no. of patients					
Respiratory	NA	47	40	7	-
Abdominal	NA	3	2	1	-
Vascular	NA	7	4	3	-
Urinary	NA	3	2	1	-
Other	NA	4	3	1	-
Isolates, no. of patients					
Gram positive	NA	15	13	2	-
Gram negative	NA	6	4	2	-
Virus	NA	29	24	5	-
Fungus	NA	2	2	0	-
Miscellaneous	NA	6	4	2	-
Other	NA	3	3	0	-
Sepsis shock	NA	12	4	8	**<0.0001**
pSOFA socre	NA	5(4-8)	5(2-6)	8(7-9)	**0.0002**
ICU stay	NA	7(5-8)	7(5-8)	9(2-8)	0.252

Categorical variables are expressed as *n* (%), and continuous variables were expressed as median (interquartile range). Comparisons between sepsis patients and sepsis shock patients, survivors and non-survivors were performed with chi-square or Fisher exact tests for qualitative variables and Mann-Whitney *U* for quantitative variables. pSOFA score was measured after 24 hours of ICU stay.

Abbreviations: WBC, white blood cell; CRP, C-reactive protein; PCT, procalcitonin; pSOFA, pediatric Sequential Organ Failure Assessment; ICU, intensive care unit; NA, not applicable.

Supplemental Table 4. Clinical characteristics of sepsis patients and healthy volunteers in the pediatric validation cohort.

Characteristics	Healthy controls (*n* = 98)	Infection controls (*n* = 102)	All sepsis patients (*n* = 145)	Sepsis survivors patients (*n* = 105)	Sepsis non-survivors patients (*n* = 40)	*P* value (Survivors VS Nonsurvivors)
Male/Female	52/46	57/45	79/64	57/48	24/16	0.476
Age, years	8(6-12)	5(2-8)	7(4-10)	6(3-8)	7(3-11)	0.690
WBC, 10^9/L	NA	10.4(6.5-12.2)	14.1(5.6-19.5)	15.8(5.9-20.4)	9.2(5.5-10.5)	0.103
CRP, mg/L	NA	19.0(1.8-30.4)	50.0(4.5-70.1)	45.7(4.2-68.7)	67.9(4.8-126.5)	0.055
PCT, ng/mL	NA	0.5(0.1-0.4)	14.3(0.7-16.2)	11.0(0.6-11.0)	28.4(2.4-40.0)	**0.0003**
Infection site, no. of patients						
Respiratory	NA	82	69	44	27	-
Abdominal	NA	10	30	18	12	-
Vascular	NA	0	14	8	6	-
Urinary	NA	6	23	14	9	-
Other	NA	9	31	21	10	-
Isolates, no. of patients						
Gram positive	NA	17	33	23	10	-
Gram negative	NA	26	28	20	8	-
Virus	NA	49	60	45	15	-
Fungus	NA	0	7	5	2	-
Miscellaneous	NA	7	10	6	4	-
Other	NA	3	5	6	1	-
Sepsis shock	NA	-	44	23	21	**<0.0001**
pSOFA score	NA	0.07(0-1)	7(4-10)	6(3-7)	12(7-15)	**0.0001**
ICU stay	NA	5(4-7)	13(7-15)	15(8-17)	8(4-12)	0.294

Categorical variables are expressed as *n* (%), and continuous variables were expressed as median (interquartile range). Comparisons between sepsis patients and sepsis shock patients, survivors and non-survivors were performed with chi-square or Fisher exact tests for qualitative variables and Mann-Whitney *U* for quantitative variables. pSOFA score was measured after 24 hours of ICU stay.

Abbreviations: WBC, white blood cell; CRP, C-reactive protein; PCT, procalcitonin; pSOFA, pediatric Sequential Organ Failure Assessment; ICU, intensive care unit; NA, not applicable.

Supplemental Table 5. Association between admission concentrations of serum IGFBP6 and 28-day mortality for the discovery cohort of adults with sepsis.

Parameters	Standard error	Wald	IQR Hazard Ratio (95% CI)	*P* value
Univariate Cox models				
IGFBP6	0.003	16.884	1.011 (1.006 – 1.016)	**<0.001**
SOFA	0.098	16.339	1.487 (1.227 – 1.803)	**<0.001**
CRP	0.004	5.321	1.009 (1.001 – 1.016)	**0.021**
PCT	0.002	1.583	1.003 (0.998 – 1.007)	0.208
WBC	0.037	0.329	0.979 (0.911 – 1.052)	0.566
Neu	0.041	0.863	0.962 (0.888 – 1.043)	0.353
PLT	0.002	0.163	1.001 (0.997 – 1.005)	0.687
Cr	0.001	7.906	1.004(1.001 – 1.006)	**0.005**
Multivariate Cox models				
IGFBP6	0.003	5.258	1.008 (1.001 – 1.015)	**0.022**
SOFA	0.196	4.076	1.485 (1.012 – 2.181)	**0.043**
CRP	0.005	3.804	1.011 (1.000 – 1.022)	0.051
PCT	0.004	1.934	0.995 (0.988 – 1.002)	0.164
WBC	0.158	0.312	0.915 (0.671 – 1.248)	0.577
Neu	0.168	0.052	0.963 (0.693 – 1.337)	0.820
PLT	0.003	0.144	0.999 (0.994 – 1.004)	0.705
Cr	0.002	2.521	1.003 (0.999 – 1.008)	0.112

Boldface value indicate significance at *P*<0.05. IQR, interquartile range; CI, confidence interval.

Supplemental Table 6. Association between admission concentrations of serum IGFBP6 and 28-day mortality in the validation cohort of adults with sepsis.

Parameters	Standard error	Wald	IQR Hazard Ratio (95% CI)	*P* value
Univariate Cox models				
IGFBP6	0.002	45.865	1.011 (1.008 – 1.015)	**<0.0001**
SOFA	0.035	33.869	1.229 (1.146 – 1.317)	**<0.0001**
APACHE II	0.014	26.627	1.075 (1.046 – 1.105)	**<0.0001**
WBC	0.021	0.517	1.016 (0.974 – 1.059)	0.472
CRP	0.002	4.654	1.004 (1.000 – 1.007)	**0.031**
PCT	0.005	2.849	1.009 (0.999 – 1.019)	0.091
Neu	0.010	0.960	1.010 (0.991 – 1.029)	0.327
Multivariate Cox models				
IGFBP6	0.002	17.8	1.010 (1.006 – 1.015)	**<0.0001**
SOFA	0.045	7.871	1.146 (1.050 – 1.252)	**0.002**
APACHE II	0.021	0.971	1.021 (0.980 – 1.064)	0.324
WBC	0.021	0.653	1.107 (0.975 – 1.060)	0.441
CRP	0.002	0.182	0.999 (0.995 – 1.003)	0.564

PCT	0.006	0.011	1.001 (0.989 – 1.013)	0.336
Neu	0.010	0.547	0.992 (0.972 – 1.011)	0.392

Boldface value indicate significance at *P*<0.05. IQR, interquartile range; CI, confidence interval.

Supplemental Table 7. Correlation coefficient of ROC curve for predicting sepsis death in the adult discovery cohort.

Parameter	Cutoff value	AUC (95% CI)	Sensitivity (%)	Specificity (%)	Youden index (%)	*P* value
IGFBP6	221.1	0.89 (0.82 – 0.96)	87.50	82.67	70.17	**<0.0001**
SOFA	7.50	0.89 (0.82 – 0.96)	87.50	78.67	66.17	**<0.0001**
CRP	121.7	0.68 (0.55 – 0.81)	68.75	63.38	32.13	**0.0273**
PCT	20.49	0.69 (0.54 – 0.83)	73.33	64.71	38.04	**0.0224**
WBC	8.710	0.57 (0.39 – 0.76)	58.33	67.74	26.07	0.4284
PLT	190.0	0.55 (0.36 – 0.75)	50.00	72.88	22.88	0.5702

Boldface value indicate significance at *P*<0.05.

Supplemental Table 8. Correlation coefficient of ROC curve to predict death with sepsis in the adult validation cohort.

Parameter	Cutoff value	AUC (95% CI)	Sensitivity (%)	Specificity (%)	Youden index (%)	*P* value
IGFBP6	209.7	0.76 (0.69 – 0.83)	77.05	71.57	48.62	**<0.0001**
SOFA	5.50	0.75 (0.67 – 0.82)	93.44	41.18	34.62	**<0.0001**
APACHE II	17.50	0.68 (0.60 – 0.77)	72.13	58.82	30.95	**0.0001**
CRP	28.53	0.59 (0.51 – 0.68)	90.16	29.41	19.57	**0.0465**
PCT	15.31	0.65 (0.56 – 0.76)	56.67	75.49	32.16	**0.0019**
WBC	21.36	0.50 (0.41 – 0.59)	13.11	95.10	8.21	0.9891

Boldface value indicate significance at *P*<0.05.

Supplemental Table 9. Association between admission concentrations of serum IGFBP6 and

28-day mortality of septic patients in the pediatric discovery cohort.

Parameters	Standard error	Wald	IQR Hazard Ratio (95% CI)	*P* value
Univariate Cox models				
IGFBP6	0.003	16.403	1.011 (1.006 – 1.016)	**<0.001**
pSOFA	0.123	6.631	1.374 (1.079 – 1.750)	**0.010**
WBC	0.047	1.659	0.941 (0.859 – 1.032)	0.198
PCT	0.013	13.028	1.049 (1.022 – 1.077)	**<0.001**
CRP	0.005	0.028	1.001 (0.992 – 1.010)	0.868
Neu	0.054	0.494	0.963 (0.866 – 1.070)	0.482
PLT	0.002	6.534	0.994 (0.990 – 0.999)	**0.011**
Multivariate Cox models				
IGFBP6	0.008	4.817	1.018 (1.002 – 1.035)	**0.028**
pSOFA	0.243	2.628	1.484 (0.921 – 2.391)	0.105
WBC	0.311	1.058	0.726 (0.395 – 1.336)	0.304
PCT	0.028	1.210	0.990 (0.979 – 1.002)	0.102
CRP	0.006	2.670	1.081 (0.967 – 1.096)	0.216
Neu	0.363	0.881	1.406 (0.690 – 2.862)	0.348
PLT	0.003	1.014	0.997 (0.991 – 1.003)	0.314

Boldface value indicate significance at *P*<0.05. IQR, interquartile range; CI, confidence interval.

Supplemental Table 10. Association between admission concentrations of serum IGFBP6 and 28-day mortality of sepsis patients in the pediatric validation cohort.

Parameters	Standard error	Wald	IQR Hazard Ratio (95% CI)	*P* value
Univariate Cox models				
IGFBP6	0.002	52.990	1.014 (1.010 – 1.018)	**<0.001**
pSOFA	0.037	56.516	1.323 (1.230 – 1.423)	**<0.001**
PCT	0.002	52.756	1.016 (1.012 – 1.020)	**<0.001**
CRP	0.003	3.338	1.006 (1.000 – 1.012)	**0.068**
Neu	0.025	1.655	0.969 (0.923 – 1.017)	0.198
Multivariate Cox models				
IGFBP6	0.007	4.727	1.007 (1.001 – 1.013)	**0.030**
pSOFA	0.061	8.612	1.194 (1.061 – 1.345)	**0.003**
PCT	0.004	0.332	1.002 (0.994 – 1.011)	0.564
CRP	0.004	0.590	0.997 (0.989 – 1.005)	0.442
Neu	0.030	0.843	0.972 (0.916 – 1.032)	0.358

Boldface value indicate significance at *P*<0.05. IQR, interquartile range; CI, confidence interval.

Supplemental Table 11. Correlation coefficient of ROC curve for predicting sepsis death in the pediatric discovery cohort.

Parameter	Cutoff value	AUC (95% CI)	Sensitivity (%)	Specificity (%)	Youden index (%)	*P* value
IGFBP6	242.4	0.88 (0.75 – 1.00)	72.73	100	72.73	**<0.0001**
pSOFA	6.500	0.82 (0.69 – 0.94)	81.82	78.00	59.82	**0.0008**
CRP	178.1	0.58 (0.38 – 0.79)	27.27	95.92	23.19	0.3900
PCT	1.440	0.68 (0.48 – 0.89)	72.73	71.74	44.47	0.0587
WBC	10.34	0.61 (0.42 – 0.81)	45.45	79.59	25.04	0.2478
PLT	255.0	0.83 (0.66 – 0.99)	81.82	87.76	69.58	**0.0007**

Boldface value indicate significance at *P*<0.05.

Supplemental Table 12. Correlation coefficient of ROC curve for predicting sepsis death in the pediatric validation cohort.

Parameter	Cutoff value	AUC (95% CI)	Sensitivity (%)	Specificity (%)	Youden index (%)	*P* value
IGFBP6	165.7	0.83 (0.75 – 0.90)	62.50	88.57	51.07	**<0.0001**
pSOFA	9.500	0.79 (0.71 – 0.88)	67.50	86.67	54.17	**<0.0001**
CRP	118.5	0.57 (0.46 – 0.68)	25.00	94.29	19.29	0.1751
PCT	1.905	0.72 (0.63 – 0.81)	82.50	50.48	32.98	**<0.0001**
WBC	8.815	0.65 (0.54 – 0.75)	60.00	69.52	29.52	**0.0067**
PLT	75.50	0.60 (0.49 – 0.72)	35.00	91.43	26.43	0.0610

Boldface value indicate significance at *P*<0.05.

Supplemental Table 13. The primer sequence of the CCL2 promoter.

Primer Name	Sequence (5’to 3’)	Modification
CCL2-F	AAGAAATCTAAACCTGGAAAGCTGAATTAA	
CCL2-R	TTAATTCAGCTTTCCAGGTTTAGATTTCTT	
CCL2-F-BIO	AAGAAATCTAAACCTGGAAAGCTGAATTAA	5’BIO

mtCCL2-F	AAGAAATCTAAACGACGAAAGCTGAATTAA	
mtCCL2-R	TTAATTCAGCTTTCGTCGTTTAGATTTCTT	
mtCCL2-F-BIO	AAGAAATCTAAACGACGAAAGCTGAATTAA	5’BIO

Note: F, forward primer; R, reverse prime

Supplemental Table 14. siRNA sequences used to target PHB2 gene.

Name	Forward 5’- 3’	Reverse 5’- 3’
siNC	UUCUCCGAACGUGUCACGUTT	ACGUGACACGUUCGGAGAATT
siPHB2-1 (PHB2-mus-942)	CCACAUCACAGAACCGAAUTT	AUUCGGUUCUGUGAUGUGGTT
siPHB2-2 (PHB2-mus-544)	GCUGCCGUCCAUUGUUAAUTT	AUUAACAAUGGACGGCAGCTT
siPHB2-3 (PHB2-mus-887)	GGCUAUAUCAAGCUCCGAATT	UUCGGAGCUUGAUAUAGCCTT

Note: siPHB2, small interfering PHB2

KEY RESOURCES TABLE

REAGENT or RESOURCE	SOURCE	IDENTIFIER
**Antibodies and fluorescence dyes**		
Mouse IGFBP-6 Antibody	R&D Systems	Cat# AF776-SP; RRID: AB_355592
DAPI	Thermofisher Scientific	Cat# D3571; RRID:AB_2307445
TRITC Phalloidin	Solarbio	Cat# CA1610
APC anti-mouse/human CD11b	BioLegend	Cat# 101211, Clone: M1/70<br>RRID:AB_312794
FITC anti-mouse Ly-6G	BioLegend	Cat# 127605, Clone: 1A8<br>RRID:AB_1236488
PE anti-mouse F4/80	BioLegend	Cat# 111703, Clone: W20065D<br>RRID:AB_2936728
TruStain FcX (anti-mouse CD16/32)	BioLegend	Cat# 101319; Clone: 93<br>RRID:AB_1574973
Anti -F4/80 Rabbit pAb	ServiceBio	Cat# GB113373, RRID:AB_2938980
Fluorescein (FITC)-AffiniPure Goat Anti-Rabbit IgG (H+L)	Jackson ImmunoResearch Labs	Cat# 111-095-003, RRID:AB_2337972
Cy3 conjugated Goat Anti-Rabbit IgG	ServiceBio	Cat# GB21303, RRID:AB_2861435
Anti-MCP1 Rabbit Antibody	ServiceBio	Cat# GB11199, RRID:AB_2922435
Phospho-Stat1 (Tyr701) Rabbit mAb	Cell Signaling Technology	Cat# 9167, Clone: 58D6<br>RRID:AB_561284
Stat1 (D1K9Y) Rabbit mAb	Cell Signaling Technology	Cat# 14994, Clone: D1K9Y<br>RRID:AB_2737027
Phospho-Akt (Ser473) Rabbit mAb	Cell Signaling Technology	Cat# 4060, Clone: D9E<br>RRID:AB_2315049
Akt (pan) (C67E7) Rabbit mAb	Cell Signaling Technology	Cat# 4691, RRID:AB_915783
Phospho-Stat3 (Tyr705) Rabbit mAb	Cell Signaling Technology	Cat# 9145, Clone: D3A7<br>RRID:AB_2491009
Stat3 (D3Z2G) Rabbit mAb	Cell Signaling Technology	Cat# 12640, RRID:AB_2629499
Phospho-NF-κB p65 (Ser536) Rabbit mAb	Cell Signaling Technology	Cat# 3033, Clone: 93H1<br>RRID:AB_331284
NF-kappaB p65 Rabbit mAb	Cell Signaling Technology	Cat# 8242, Clone: D14E12<br>RRID:AB_10859369
Phospho-p44/42 MAPK (Erk1/2) (Thr202/Tyr204) Rabbit mAb	Cell Signaling Technology	Cat# 4370, Clone: D13.14.4E<br>RRID:AB_2315112
p44/42 MAPK (Erk1/2) (137F5) Rabbit mAb	Cell Signaling Technology	Cat# 4695, RRID:AB_390779
Phospho-p38 MAPK (Thr180/Tyr182) Rabbit mAb	Cell Signaling Technology	Cat# 4511, Clone: D3F9<br>RRID:AB_2139682
Phospho-SAPK/JNK (Thr183/Tyr185) Rabbit mAb	Cell Signaling Technology	Cat# 4668, Clone: 81E11<br>RRID:AB_823588
SAPK/JNK Antibody	Cell Signaling Technology	Cat# 9252, RRID:AB_2250373
p38 MAPK Rabbit mAb	Cell Signaling Technology	Cat# 8690, Clone:D13E1

		RRID:AB_10999090
β-Actin Mouse mAb	Cell Signaling Technology	Cat# 3700, Clone:8H10D10<br>RRID:AB_2242334
GAPDH XP Rabbit mAb	Cell Signaling Technology	Cat# 5174, Clone:D16H11<br>RRID:AB_10622025
Anti - alpha smooth muscle Actin Rabbit pAb	ServiceBio	Cat# GB111364, RRID:AB_2910228
Prohibitin 2 antibody	Proteintech	Cat# 12295-1-AP, RRID:AB_2164779
Pan Phospho-Tyrosine Rabbit mAb	Abclonal	Cat# AP1316, RRID:AB_2864021
**Chemicals, peptides, and recombinant proteins**		
Recombinant Mouse IGFBP-6 Protein	R&D Systems	Cat# 776-B6
Recombinant Mouse CCL2/JE/MCP-1 Protein	R&D Systems	Cat# 479-JE
Mouse M-CSF Recombinant Protein	PeproTech	Cat# 315-02
Recombinant Human M-CSF Protein	R&D Systems	Cat#: 216-MCC
RO8191	MedChemExpress	Cat# HY-W063968
2-NP	MedChemExpress	Cat# HY-W013523
TransIT® -QR Hydrodynamic Delivery Solution	Mirusbio	Cat# MIR 5240
Lipofectamine™ 2000	Thermofisher Scientific	Cat# 11668030
Thioglycollate Medium	Solarbio	Cat# LA4590
Clodronate-encapsulated liposomes	LIPOSOMA	Cat# C-005
Ficoll-Paque PREMIUM	cytiva	Cat# 17544602
**Critical commercial assays**		
Human IGFBP-6 DuoSet ELISA	R&D Systems	Cat# DY-876
Mouse IGFBP-6 DuoSet ELISA	R&D Systems	Cat# DY-776
Mouse CXCL1/KC DuoSet ELISA	R&D Systems	Cat# DY-453
Mouse IL-1β ELISA	BioLegend	Cat# 432601
Mouse TNF-α ELISA	BioLegend	Cat# 430901
Mouse IFN-γ ELISA	BioLegend	Cat# 430801
Mouse IL-4 ELISA	BioLegend	Cat# 431101
Mouse IL-6 ELISA	BioLegend	Cat# 431301
Mouse IL-10 ELISA	BioLegend	Cat# 431411
Mouse IL-17A ELISA	BioLegend	Cat# 432501
Mouse CCL2/MCP-1 ELISA	BioLegend	Cat# 432704
Mouse CCL3/MIP-1 alpha DuoSet ELISA	R&D Systems	Cat# DY-450
Mouse CXCL10/IP-10/CRG-2 DuoSet ELISA	R&D Systems	Cat# DY-466
Mouse CXCL12/SDF-1 DuoSet ELISA	R&D Systems	Cat# DY-460
Mouse CXCL16 DuoSet ELISA	R&D Systems	Cat# DY-503

Neutrophil Isolation Kit, mouse	Miltenyi Biotec	Cat# 130-097-658
CD14 MicroBeads, human	Miltenyi Biotec	Cat# 130-050-201
EMSA/Gel-Shift Kit	Beyotime	Cat# GS002
Pierce Co-Immunoprecipitation (Co-IP) Kit	Thermofisher Scientific	Cat# 26149
**Experimental models: Organisms/strains**		
WT	Charles River	C57BL/6N
Igfbp6$^{-/-}$ (Igfbp6^{em1})	Cyagen Biosciences Inc	MGI:96441 (backcrossed to C57BL/6N)
Tlr2$^{-/-}$ (Tlr2^{em1C})	Cyagen Biosciences Inc	MGI:1346060 (backcrossed to C57BL/6)
Tlr4$^{-/-}$ (Tlr4^{em1C})	Cyagen Biosciences Inc	MGI:96824 (backcrossed to C57BL/6)
Pseudomonas aeruginosa	ATCC 27853	
Staphylococcus aureus	ATCC 25923	
Murine Lung Epithelial-12 (MLE-12)	Sunncell	Cat# SNL-414
MODE-K	BeNa Culture Collection	Cat# BNCC338300

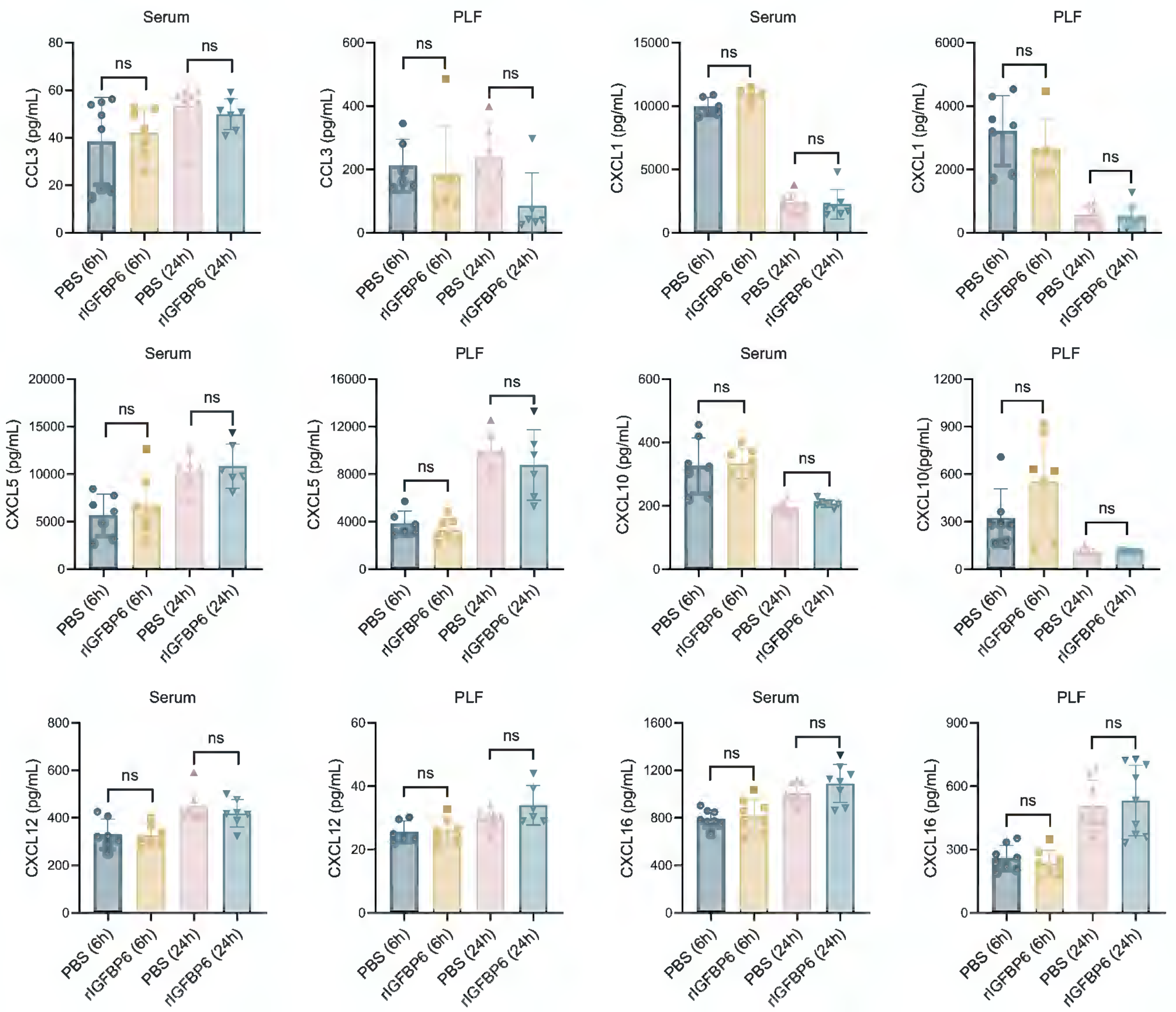
Serum
PLF
CCL3 (pg/mL)
CXCL1 (pg/mL)
CXCL5 (pg/mL)
CXCL10 (pg/mL)
CXCL10(pg/mL)
CXCL12 (pg/mL)
CXCL16 (pg/mL)
ns
PBS (6h)
rIGFBP6 (6h)
PBS (24h)
rIGFBP6 (24h)

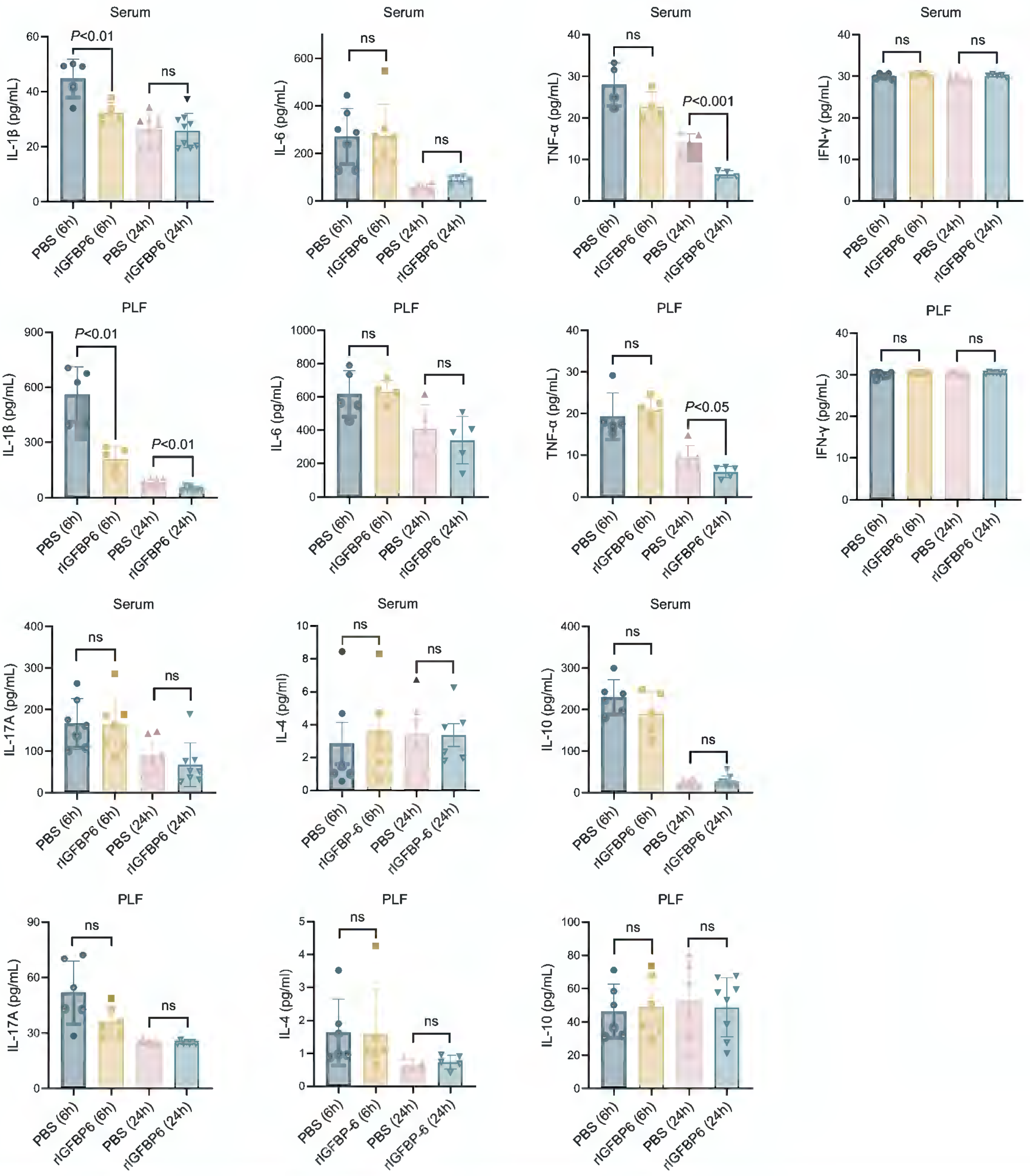

Serum
IL-1β (pg/mL)
P<0.01
ns
IL-6 (pg/mL)
TNF-α (pg/mL)
P<0.001
IFN-γ (pg/mL)
PLF
P<0.05
IL-17A (pg/mL)
IL-4 (pg/ml)
IL-10 (pg/mL)
PBS (6h)
rIGFBP6 (6h)
PBS (24h)
rIGFBP6 (24h)
rIGFBP-6 (6h)
rIGFBP-6 (24h)

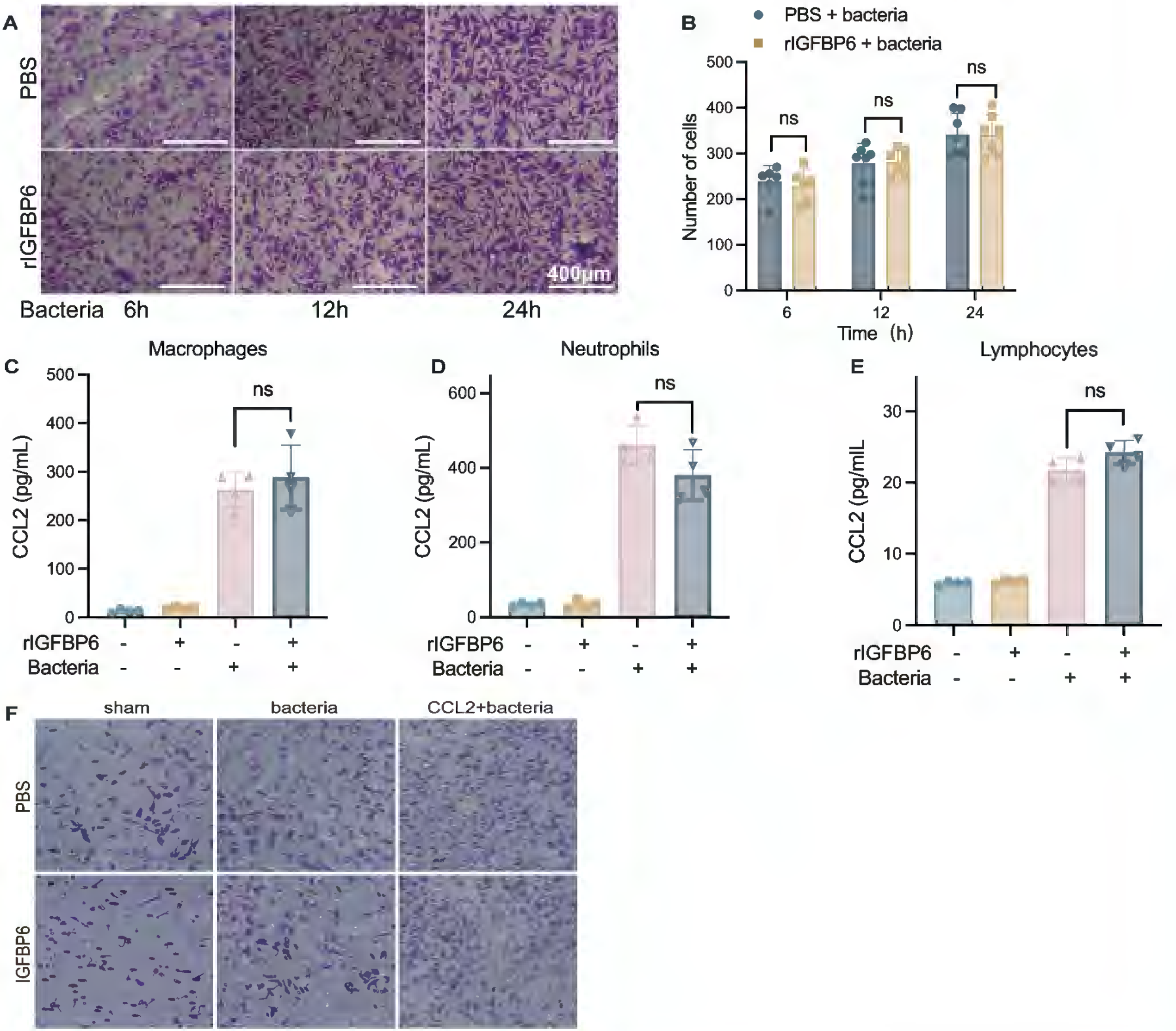

A
PBS
rIGFBP6
400μm
Bacteria 6h
12h
24h
B
PBS + bacteria
rIGFBP6 + bacteria
Number of cells
ns
Time (h)
C
Macrophages
CCL2 (pg/mL)
rIGFBP6
Bacteria
D
Neutrophils
E
Lymphocytes
CCL2 (pg/mIL
F
sham
bacteria
CCL2+bacteria
PBS
IGFBP6

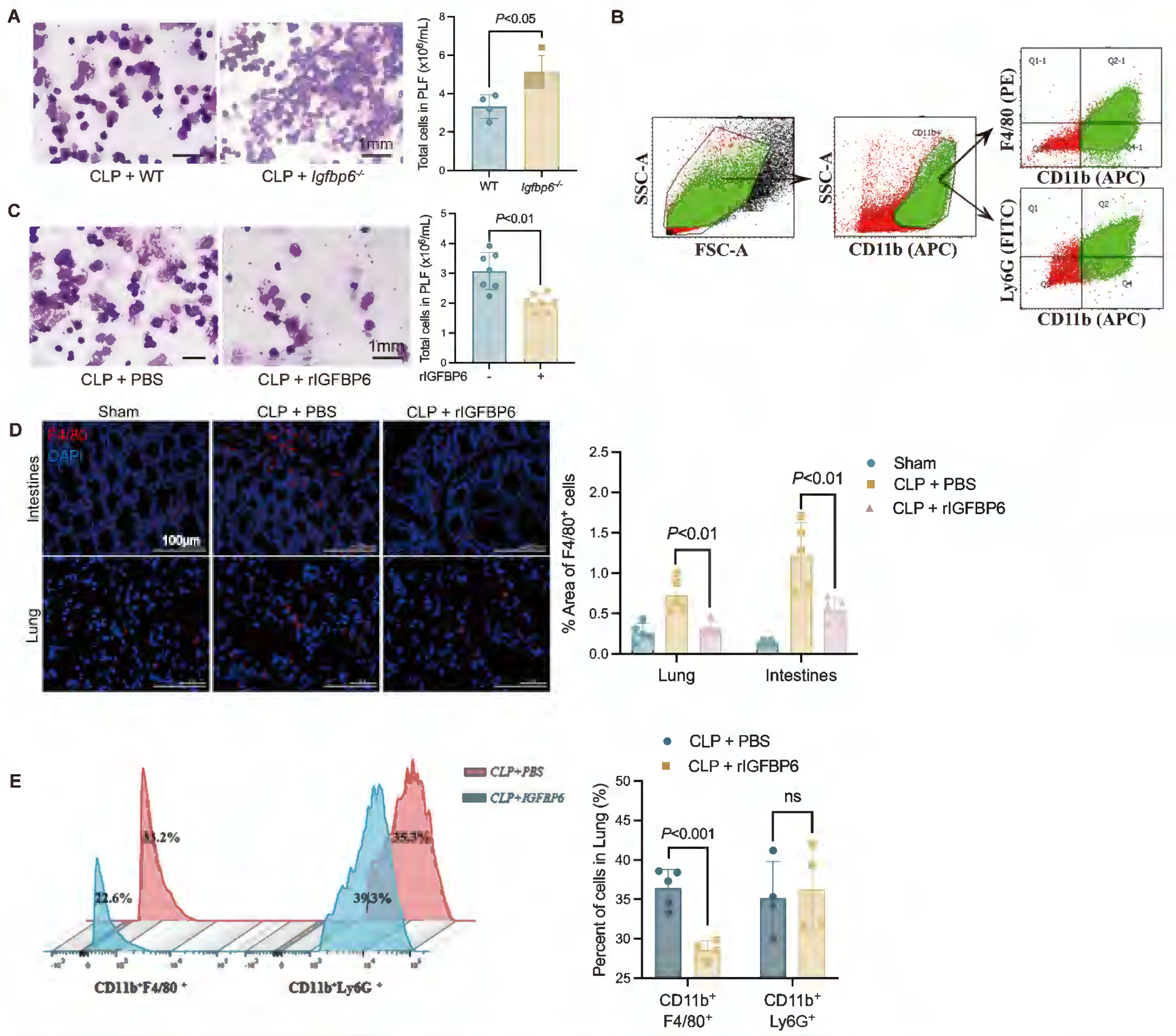
A
CLP + WT
CLP + Igfbp6-/-
1mm
Total cells in PLF (x10^6/mL)
P<0.05
WT
Igfbp6-/-
B
SSC-A
FSC-A
CD11b (APC)
CD11b+
F4/80 (PE)
Q1-1
Q2-1
Q4-1
Ly6G (FITC)
Q1
Q2
Q4
C
CLP + PBS
CLP + rIGFBP6
1mm
Total cells in PLF (x10^6/mL)
P<0.01
rIGFBP6
D
Sham
CLP + PBS
CLP + rIGFBP6
F4/80
DAPI
Intestines
Lung
100μm
% Area of F4/80+ cells
P<0.01
P<0.01
Lung
Intestines
E
CLP+PBS
CLP+IGFBP6
35.2%
22.6%
35.3%
39.3%
CD11b+F4/80+
CD11b+Ly6G+
Percent of cells in Lung (%)
P<0.001
ns
CD11b+ F4/80+
CD11b+ Ly6G+

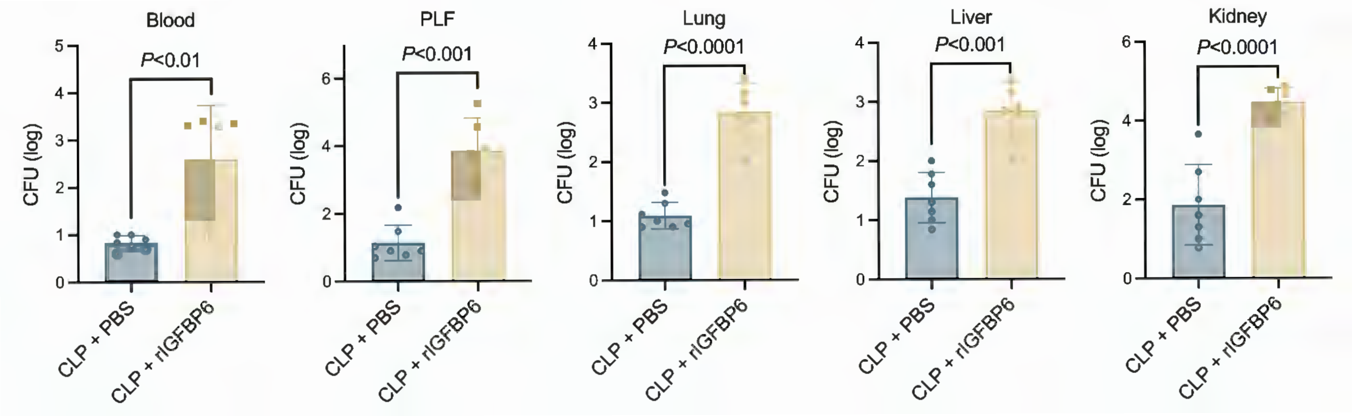

Blood
PLF
Lung
Liver
Kidney
CFU (log)
P<0.01
P<0.001
P<0.0001
P<0.001
P<0.0001
CLP + PBS
CLP + rIGFBP6

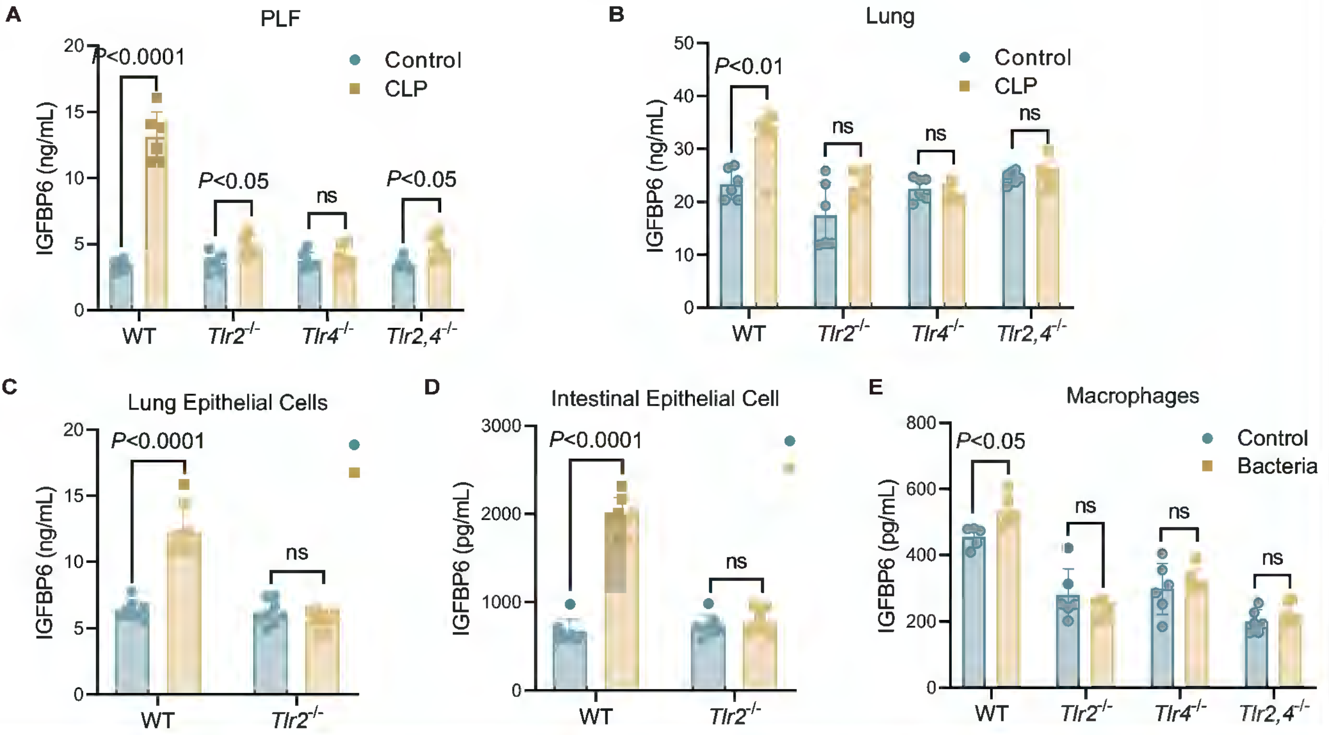
A
PLF
IGFBP6 (ng/mL)
P<0.0001
P<0.05
ns
P<0.05
Control
CLP
WT
Tlr2-/-
Tlr4-/-
Tlr2,4-/-
B
Lung
IGFBP6 (ng/mL)
P<0.01
ns
ns
ns
Control
CLP
WT
Tlr2-/-
Tlr4-/-
Tlr2,4-/-
C
Lung Epithelial Cells
IGFBP6 (ng/mL)
P<0.0001
ns
WT
Tlr2-/-
D
Intestinal Epithelial Cell
IGFBP6 (pg/mL)
P<0.0001
ns
WT
Tlr2-/-
E
Macrophages
IGFBP6 (pg/mL)
P<0.05
ns
ns
ns
Control
Bacteria
WT
Tlr2-/-
Tlr4-/-
Tlr2,4-/-

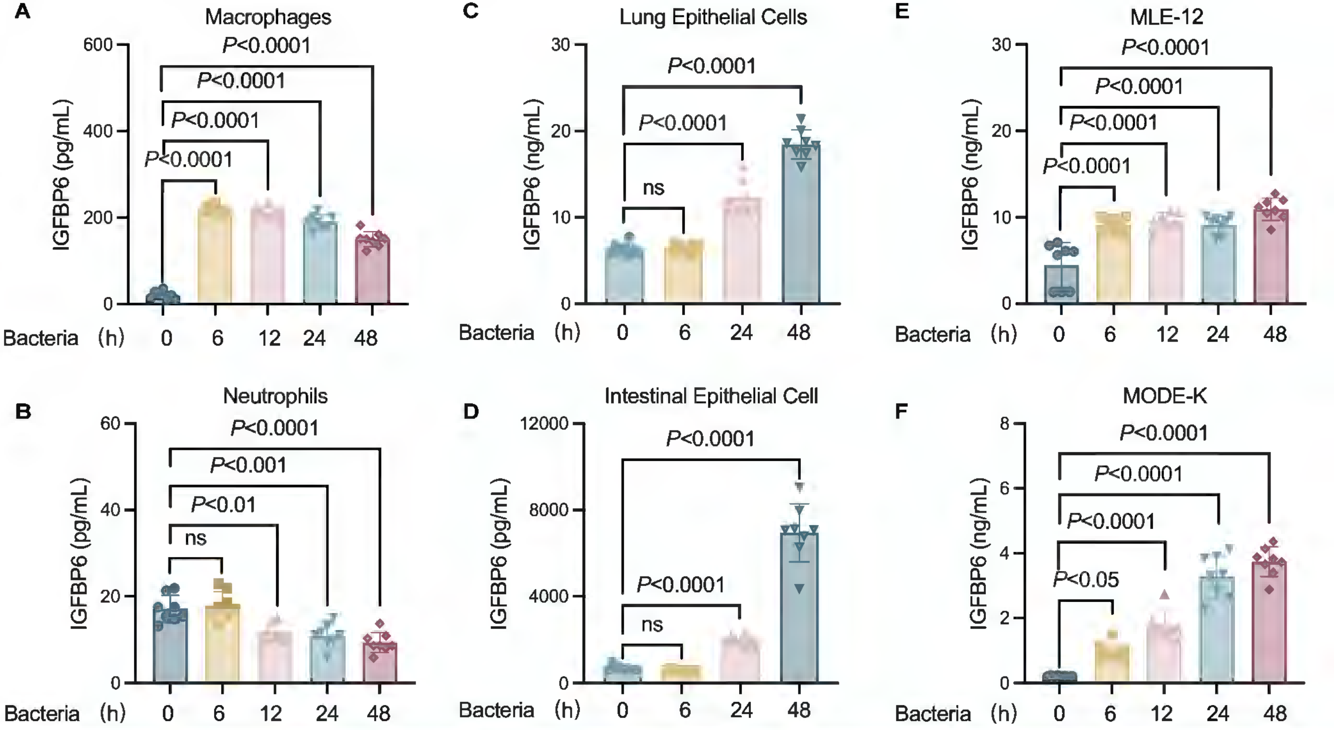
A
Macrophages
IGFBP6 (pg/mL)
P<0.0001
P<0.0001
P<0.0001
P<0.0001
Bacteria (h) 0 6 12 24 48
B
Neutrophils
IGFBP6 (pg/mL)
P<0.0001
P<0.001
P<0.01
ns
Bacteria (h) 0 6 12 24 48
C
Lung Epithelial Cells
IGFBP6 (ng/mL)
P<0.0001
P<0.0001
ns
Bacteria (h) 0 6 24 48
D
Intestinal Epithelial Cell
IGFBP6 (pg/mL)
P<0.0001
P<0.0001
ns
Bacteria (h) 0 6 24 48
E
MLE-12
IGFBP6 (ng/mL)
P<0.0001
P<0.0001
P<0.0001
P<0.0001
Bacteria (h) 0 6 12 24 48
F
MODE-K
IGFBP6 (ng/mL)
P<0.0001
P<0.0001
P<0.0001
P<0.05
Bacteria (h) 0 6 12 24 48

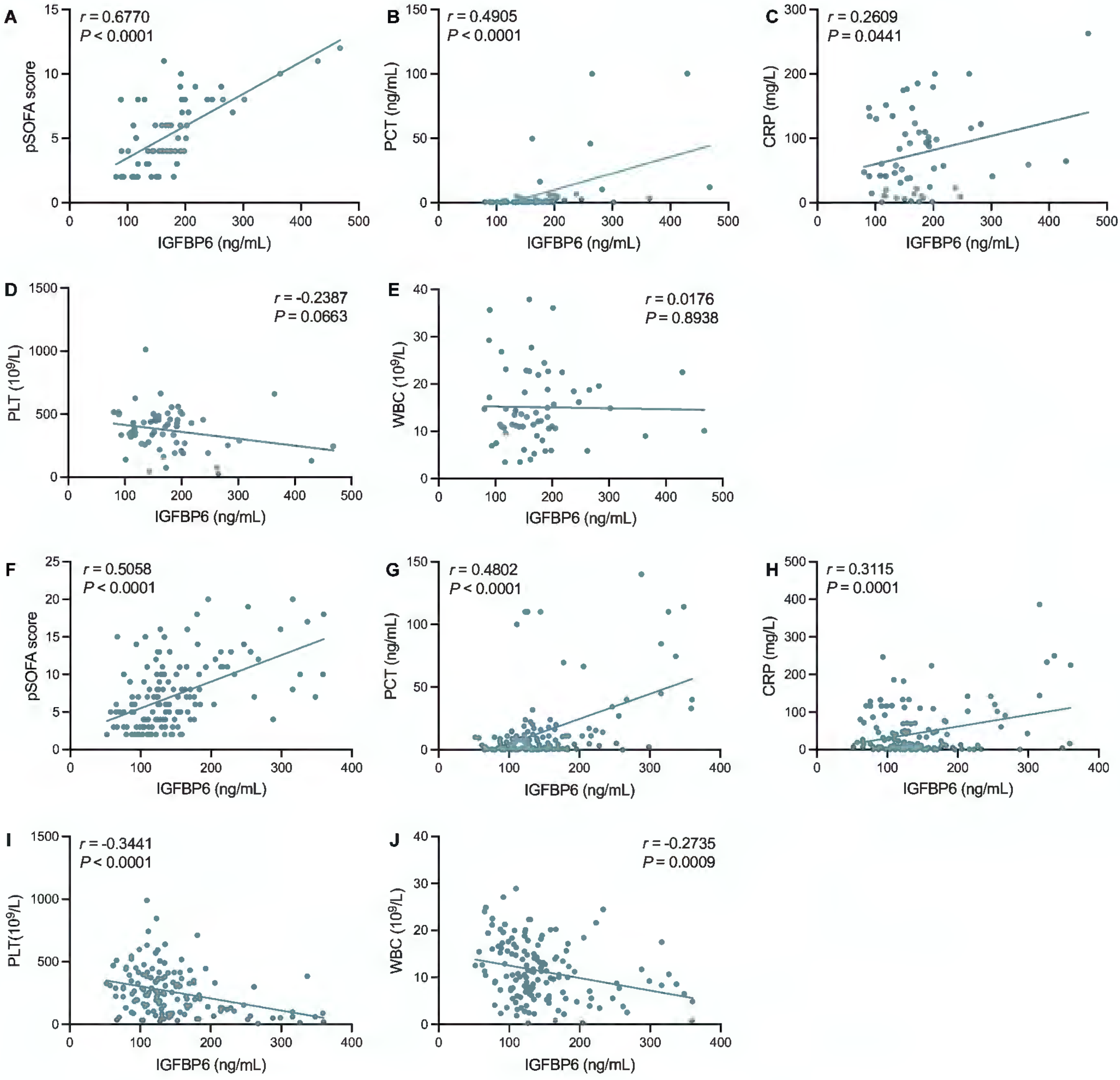
A
r = 0.6770
P < 0.0001
pSOFA score
IGFBP6 (ng/mL)
B
r = 0.4905
P < 0.0001
PCT (ng/mL)
IGFBP6 (ng/mL)
C
r = 0.2609
P = 0.0441
CRP (mg/L)
IGFBP6 (ng/mL)
D
r = -0.2387
P = 0.0663
PLT (10⁹/L)
IGFBP6 (ng/mL)
E
r = 0.0176
P = 0.8938
WBC (10⁹/L)
IGFBP6 (ng/mL)
F
r = 0.5058
P < 0.0001
pSOFA score
IGFBP6 (ng/mL)
G
r = 0.4802
P < 0.0001
PCT (ng/mL)
IGFBP6 (ng/mL)
H
r = 0.3115
P = 0.0001
CRP (mg/L)
IGFBP6 (ng/mL)
I
r = -0.3441
P < 0.0001
PLT(10⁹/L)
IGFBP6 (ng/mL)
J
r = -0.2735
P = 0.0009
WBC (10⁹/L)
IGFBP6 (ng/mL)

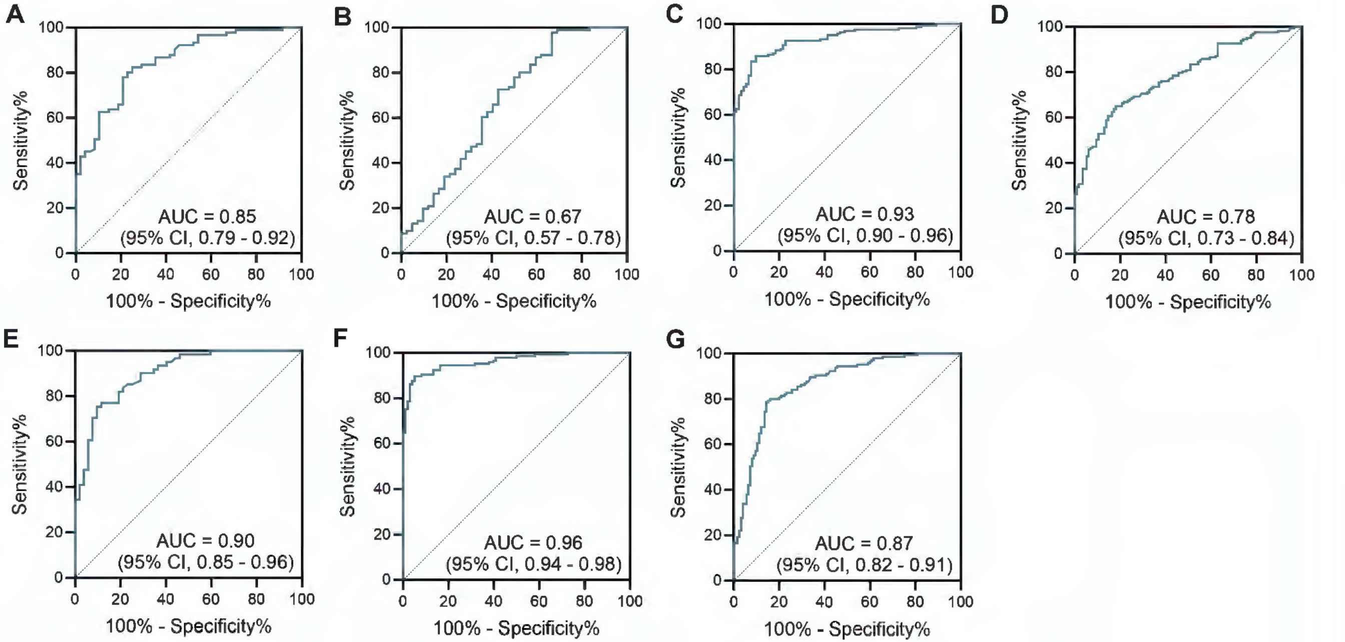
A
AUC = 0.85
(95% CI, 0.79 - 0.92)
B
AUC = 0.67
(95% CI, 0.57 - 0.78)
C
AUC = 0.93
(95% CI, 0.90 - 0.96)
D
AUC = 0.78
(95% CI, 0.73 - 0.84)
E
AUC = 0.90
(95% CI, 0.85 - 0.96)
F
AUC = 0.96
(95% CI, 0.94 - 0.98)
G
AUC = 0.87
(95% CI, 0.82 - 0.91)
Sensitivity%
100% - Specificity%

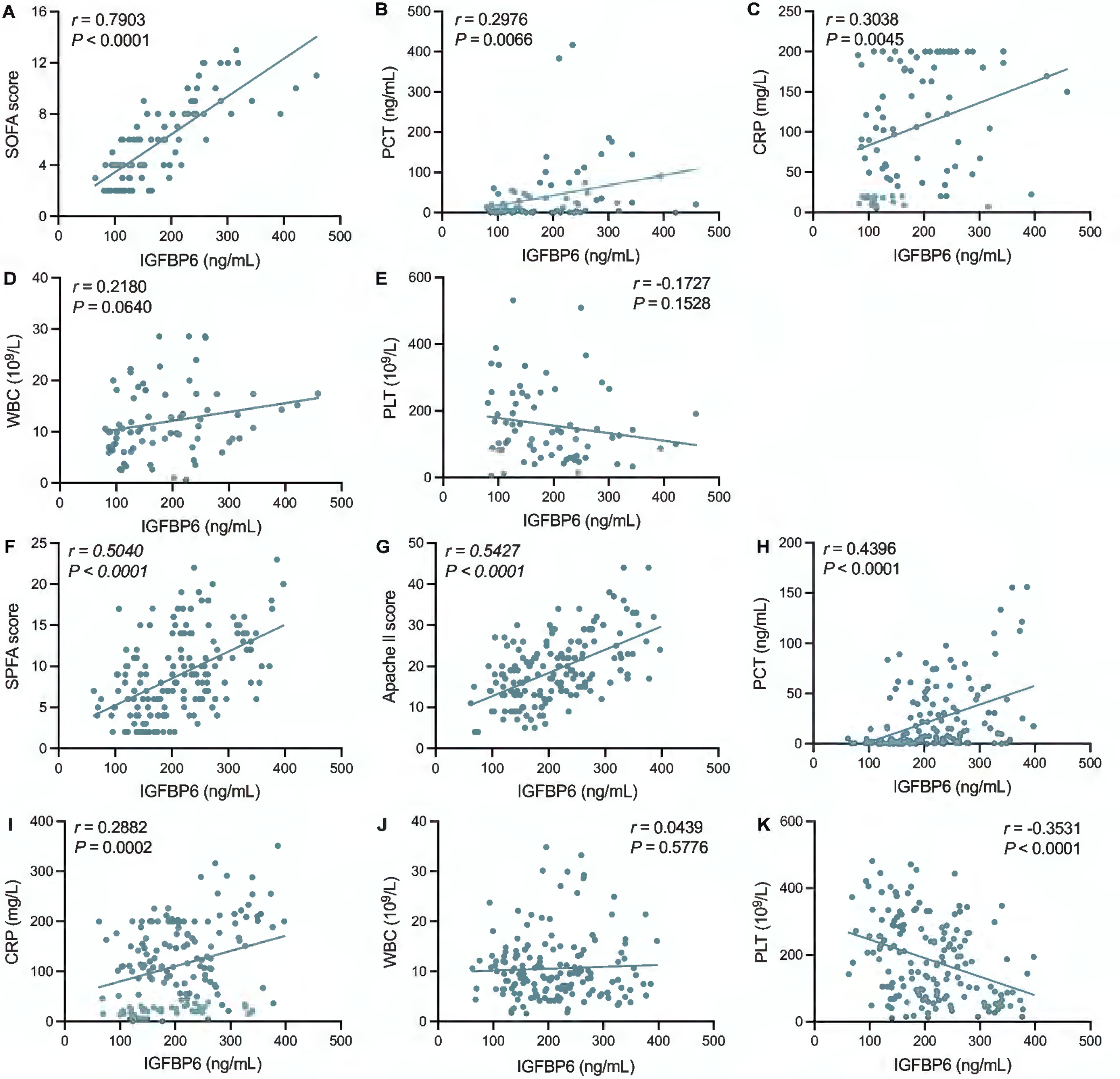

A
r = 0.7903
P < 0.0001
SOFA score
IGFBP6 (ng/mL)
B
r = 0.2976
P = 0.0066
PCT (ng/mL)
IGFBP6 (ng/mL)
C
r = 0.3038
P = 0.0045
CRP (mg/L)
IGFBP6 (ng/mL)
D
r = 0.2180
P = 0.0640
WBC (10^9/L)
IGFBP6 (ng/mL)
E
r = -0.1727
P = 0.1528
PLT (10^9/L)
IGFBP6 (ng/mL)
F
r = 0.5040
P < 0.0001
SPFA score
IGFBP6 (ng/mL)
G
r = 0.5427
P < 0.0001
Apache II score
IGFBP6 (ng/mL)
H
r = 0.4396
P < 0.0001
PCT (ng/mL)
IGFBP6 (ng/mL)
I
r = 0.2882
P = 0.0002
CRP (mg/L)
IGFBP6 (ng/mL)
J
r = 0.0439
P = 0.5776
WBC (10^9/L)
IGFBP6 (ng/mL)
K
r = -0.3531
P < 0.0001
PLT (10^9/L)
IGFBP6 (ng/mL)

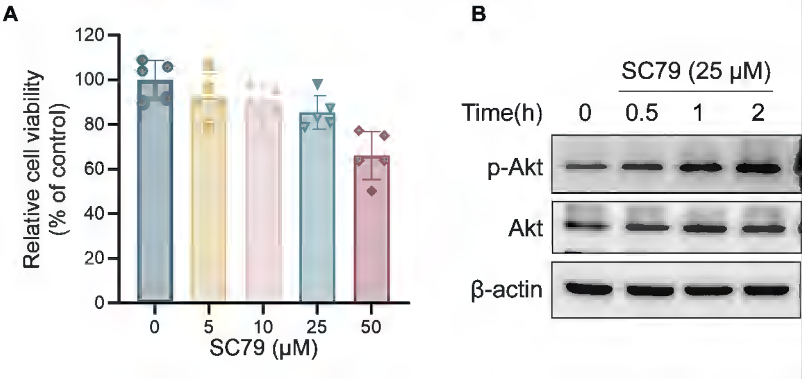
A
Relative cell viability
(% of control)
120
100
80
60
40
20
0
0
5
10
25
50
SC79 (μM)
B
SC79 (25 μM)
Time(h)
0
0.5
1
2
p-Akt
Akt
β-actin

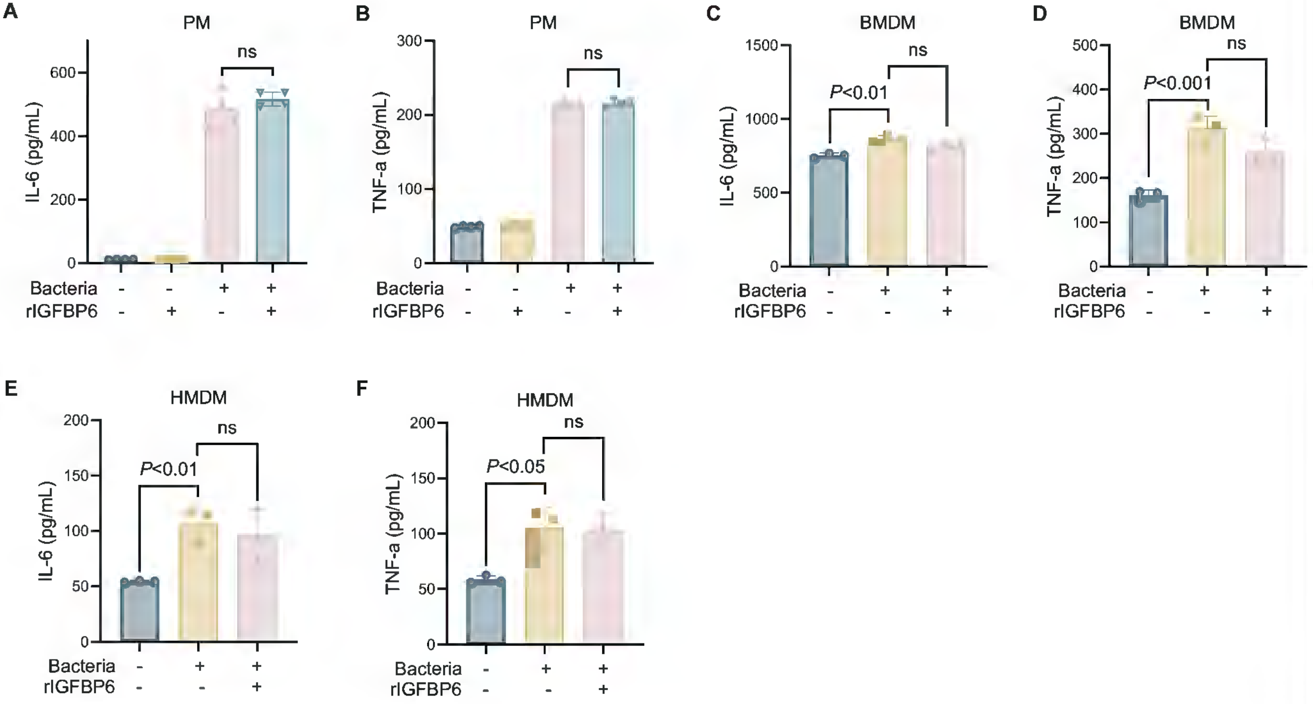
A
PM
IL-6 (pg/mL)
600
400
200
0
ns
Bacteria - - + +
rIGFBP6 - + - +
B
PM
TNF-a (pg/mL)
300
200
100
0
ns
Bacteria - - + +
rIGFBP6 - + - +
C
BMDM
IL-6 (pg/mL)
1500
1000
500
0
P<0.01
ns
Bacteria - + +
rIGFBP6 - - +
D
BMDM
TNF-a (pg/mL)
500
400
300
200
100
0
P<0.001
ns
Bacteria - + +
rIGFBP6 - - +
E
HMDM
IL-6 (pg/mL)
200
150
100
50
0
P<0.01
ns
Bacteria - + +
rIGFBP6 - - +
F
HMDM
TNF-a (pg/mL)
200
150
100
50
0
P<0.05
ns
Bacteria - + +
rIGFBP6 - - +

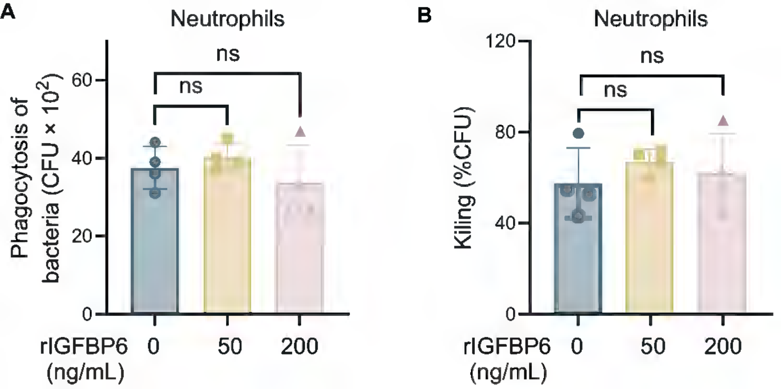

A
Neutrophils
ns
ns
Phagocytosis of bacteria (CFU × 10²)
0
20
40
60
rIGFBP6 (ng/mL)
0
50
200
B
Neutrophils
ns
ns
Kiling (%CFU)
0
40
80
120
rIGFBP6 (ng/mL)
0
50
200

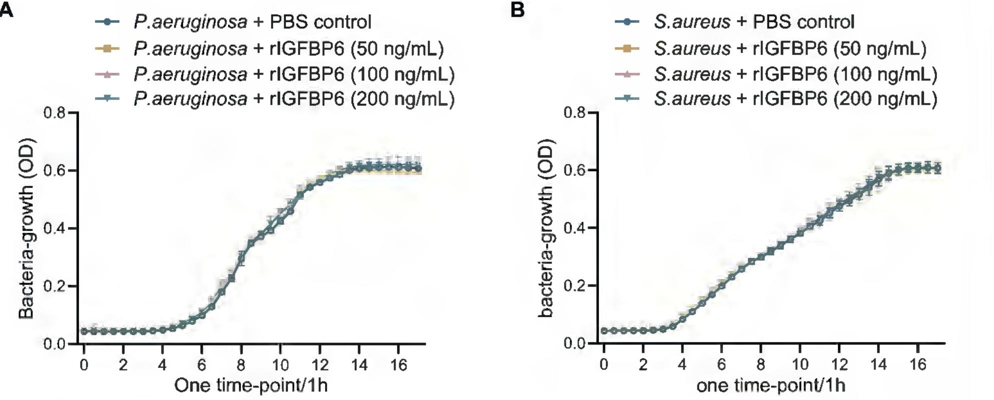
A
P.aeruginosa + PBS control
P.aeruginosa + rIGFBP6 (50 ng/mL)
P.aeruginosa + rIGFBP6 (100 ng/mL)
P.aeruginosa + rIGFBP6 (200 ng/mL)
Bacteria-growth (OD)
0.0
0.2
0.4
0.6
0.8
0
2
4
6
8
10
12
14
16
One time-point/1h
B
S.aureus + PBS control
S.aureus + rIGFBP6 (50 ng/mL)
S.aureus + rIGFBP6 (100 ng/mL)
S.aureus + rIGFBP6 (200 ng/mL)
bacteria-growth (OD)
0.0
0.2
0.4
0.6
0.8
0
2
4
6
8
10
12
14
16
one time-point/1h

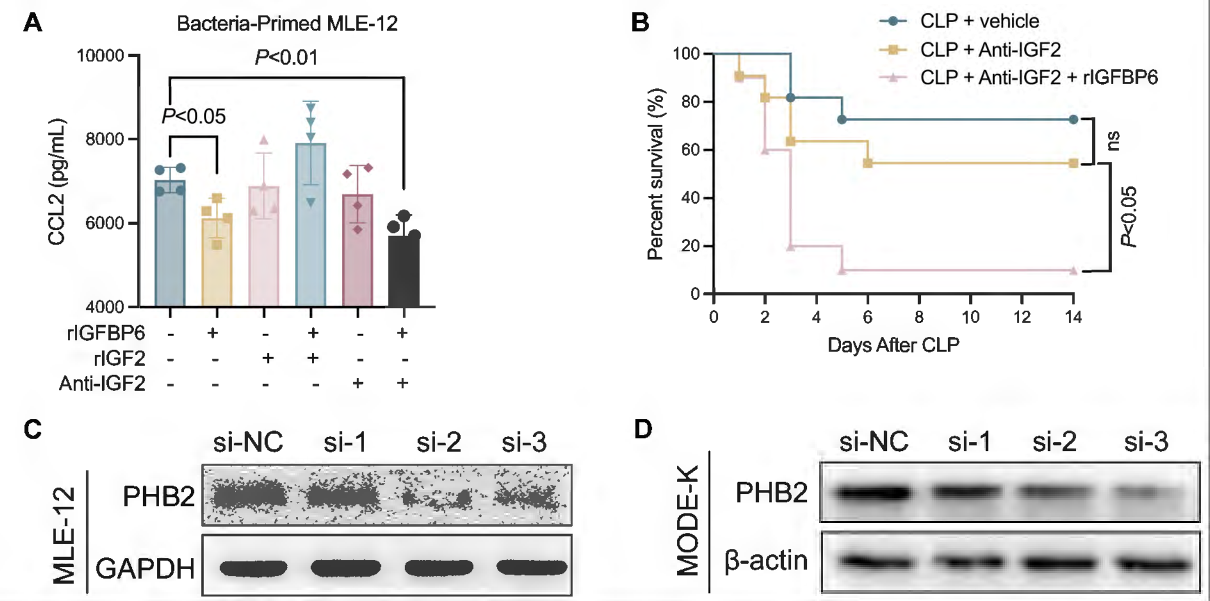
A
Bacteria-Primed MLE-12
P<0.01
P<0.05
CCL2 (pg/mL)
10000
8000
6000
4000
rIGFBP6 - + - + - +
rIGF2 - - + + - -
Anti-IGF2 - - - - + +
B
CLP + vehicle
CLP + Anti-IGF2
CLP + Anti-IGF2 + rIGFBP6
Percent survival (%)
ns
P<0.05
Days After CLP
C
si-NC si-1 si-2 si-3
MLE-12
PHB2
GAPDH
D
si-NC si-1 si-2 si-3
MODE-K
PHB2
β-actin

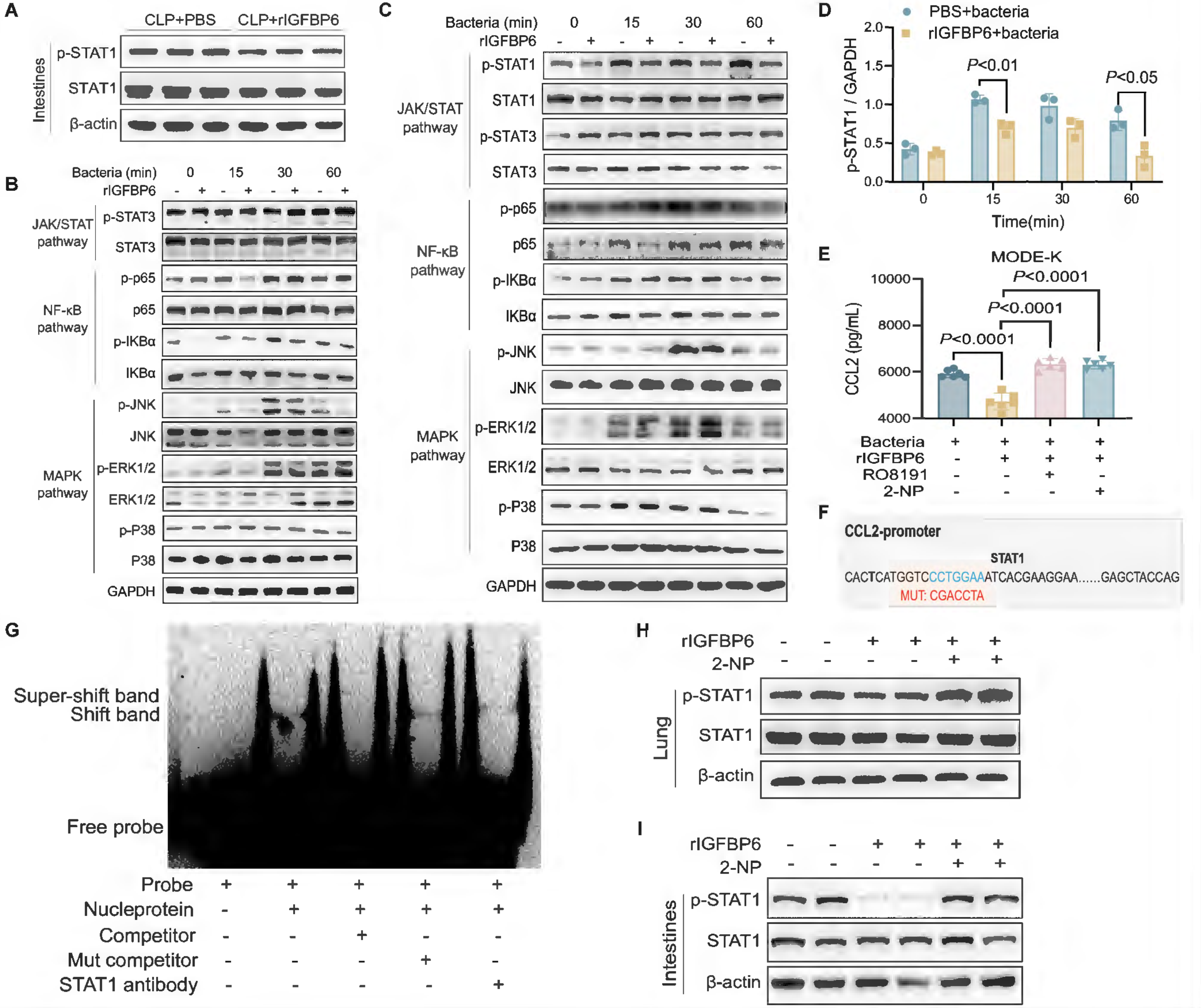
A
CLP+PBS
CLP+rIGFBP6
Intestines
p-STAT1
STAT1
β-actin
B
Bacteria (min) 0 15 30 60
rIGFBP6 - + - + - + - +
JAK/STAT pathway
p-STAT3
STAT3
NF-κB pathway
p-p65
p65
p-IKBα
IKBα
MAPK pathway
p-JNK
JNK
p-ERK1/2
ERK1/2
p-P38
P38
GAPDH
C
Bacteria (min) 0 15 30 60
rIGFBP6 - + - + - + - +
JAK/STAT pathway
p-STAT1
STAT1
p-STAT3
STAT3
NF-κB pathway
p-p65
p65
p-IKBα
IKBα
MAPK pathway
p-JNK
JNK
p-ERK1/2
ERK1/2
p-P38
P38
GAPDH
D
PBS+bacteria
rIGFBP6+bacteria
p-STAT1 / GAPDH
P<0.01
P<0.05
Time(min)
E
MODE-K
CCL2 (pg/mL)
P<0.0001
P<0.0001
P<0.0001
Bacteria + + + +
rIGFBP6 - + + +
RO8191 - - + -
2-NP - - - +
F
CCL2-promoter
STAT1
CACTCATGGTCCCTGGAAATCACGAAGGAA......GAGCTACCAG
MUT: CGACCTA
G
Super-shift band
Shift band
Free probe
Probe + + + + +
Nucleprotein - + + + +
Competitor - - + - -
Mut competitor - - - + -
STAT1 antibody - - - - +
H
rIGFBP6 - - + + + +
2-NP - - - - + +
Lung
p-STAT1
STAT1
β-actin
I
rIGFBP6 - - + + + +
2-NP - - - - + +
Intestines
p-STAT1
STAT1
β-actin

Supplemental Figure 1. Correlation analysis between circulating IGFBP6 and clinical parameters in adult patients with sepsis.

(A-E) Discovery cohort (ICU admission): IGFBP6 correlations with SOFA scores (A), PCT concentrations (B), CRP concentrations (C), WBC counts (D) and PLT counts (E) in adult patients with sepsis. **(F-K)** Validation cohort (ICU admission): IGFBP6 correlations with SOFA scores (F), APACHE II scores (G), PCT concentrations (H), CRP concentrations (I), WBC counts (J) and PLT counts (K) in adult patients with sepsis. Spearman's rank correlation analysis performed for all comparisons.

Supplemental Figure 2. Diagnostic efficacy of IGFBP6 in sepsis stratification.

(A-B) Adult discovery cohort: ROC curve of IGFBP6 for sepsis diagnosis (A), and for distinguishing diagnosis of sepsis from non-septic infection (B). **(C-D)** Adult validation cohort: ROC curve of IGFBP6 for sepsis diagnosis (C), and for distinguishing diagnosis of sepsis from non-septic infection. **(E-F)** Pediatric cohorts: ROC curve of IGFBP6 for the diagnosis of sepsis in the pediatric discovery (E) and validation (F) cohort, respectively. **(G)** ROC curve of IGFBP6 for the distinguishing diagnosis of sepsis and non-septic infection in the pediatric validation cohort.

Supplemental Figure 3. Correlation analysis between circulating IGFBP6 and clinical parameters in pediatric patients with sepsis.

(A-E) Discovery cohort (ICU admission): Correlation analysis between IGFBP6 and pSOFA scores (A), PCT concentrations (B), CRP concentrations (C), PLT counts (D) and WBC counts

(E) in pediatric patients with sepsis. **(F-J)** Validation cohort (ICU admission): Correlation of IGFBP6 levels with pSOFA scores (F), PCT concentrations (G), CRP concentrations (H), PLT counts (I) and WBC counts (J) in pediatric patients with sepsis. Spearman's rank correlation analysis performed for all comparisons.

Supplemental Figure 4. Cellular sources of sepsis-elevated IGFBP6.

(A-D) IGFBP6 concentrations in the supernatant of heat-inactivated *P.aeruginosa* (MOI = 100) challenged macrophages (A), neutrophils (B), primary lung epithelial cells (C) and intestinal epithelial cells (D) of C57BL/6N mice, were measured by ELISA at the indicated time points (*n* = 8/group). **(E-F)** IGFBP6 levels in the supernatant of MLE-12 (E) (*n* = 8/group) and MODE-K (F) (*n* = 8/group) cells stimulated with heat-inactivated *P.aeruginosa* (MOI = 100) at the indicated time points were measured by ELISA. Each dot in graphs represents data from an individual well of cell culture. One-way ANOVA in (A-F); ns, not significant.

Supplemental Figure 5. TLR2/4 signaling pathway regulates IGFBP6 expression in sepsis.

(A-B) IGFBP6 concentrations in the PLF (A) and lung (B) from $Tlr2^{-/-}$, $Tlr4^{-/-}$, $Tlr2/4^{-/-}$ and WT mice 24h post-CLP (*n* = 6/group). **(C-D)** IGFBP6 concentrations in primary lung epithelial cells (C) and intestinal epithelial cells (D) from $Tlr2^{-/-}$ and WT mice 24h post-heat-inactivated *P. aeruginosa* (MOI = 100) challenging (*n* = 8/group). **(E)** Supernatant IGFBP6 concentrations from $Tlr2^{-/-}$, $Tlr4^{-/-}$, $Tlr2/4^{-/-}$ and WT macrophages challenged with heat-inactivated *P. aeruginosa* (MOI = 100) for 24h (*n* = 5-6/group). All data are representative of three independent experiments; Each dot in graphs represents data from an individual animal tissue

and an individual well of cell culture. Student's *t* test in (A-E); ns, not significant.

Supplemental Figure 6. IGFBP6 hinders bacterial clearance in antibiotic models.

CFU counting in blood, PLF, lungs, livers and kidneys of meropenem-treated CLP models (n = 6-7/group). Each dot in graphs represents data from an individual tissue. Student's *t* test in all data.

Supplemental Figure 7. IGFBP6 impairs the chemotaxis of macrophages.

(A) Leukocyte morphological analysis in PLF from PBS- or rIGFBP6-treated mice. Wright's staining and Neubauer-improved Counting Chamber were performed for morphological observation and cell counting (n = 7/group). Scale bar = 1mm. **(B)** Flow cytometry gating strategy for macrophages ($CD11b^{+}F4/80^{+}$) and neutrophils ($CD11b^{+}Ly6G^{+}$). **(C)** Leukocyte morphology and cell count in PLF from WT or *Igfbp6*$^{-/-}$ mice post-CLP (n = 4/group). Scale bar = 1mm. **(D)** Representative immunofluorescence images (left) and quantitative analysis (right) of Cy3-labeled $F4/80^{+}$ macrophages in lung and intestinal tissues from PBS- or rIGFBP6-treated septic mice (n = 5/group). Scale bar = 100μm. **(E)** Volcano plots and bar graphs show the percentage of $CD11b^{+}F4/80^{+}$ and $CD11b^{+}Ly6G^{+}$ cells in lung tissues of septic mice (n = 4-5/group). Data in (A, D, E) are representative of three independent experiments; Each dot in graphs represents data from an individual animal tissue and an individual well of cell culture. Student's *t* test in (A); One-way ANOVA in (D); ns, not significant.

Supplemental Figure 8. IGFBP6 inhibits the secretion of epithelial cell-derived CCL2.

(A) Representative images of migrated cells in transwell migration assays. Scale bar = 400μm. **(B)** Quantitative results for (A). **(C-E)** CCL2 secretion profiles measured by ELISA in supernatants from macrophages (C), neutrophils (D), and lymphocytes (E) pre-treated with rIGFBP6 (100ng/mL) or PBS, followed by stimulation with heat-inactivated *P. aeruginosa* (MOI = 100) at indicated time points (n = 4/group). **(F)** Representative images of migrated macrophages in transwell assays stained with crystal violet. Scale bar = 1mm. Data represent three independent biological replicates. Each dot in graphs represents data from an individual well of cell culture. Student's t test in (B); One-way ANOVA in (C-E); ns, not significant.

Supplemental Figure 9. Effect of IGFBP6 on cytokine production in experimental sepsis. Protein levels of IL-1β, TNF-α, IL-10, IL-6, IFN-γ, IL-17A, and IL-4 in PLF and serum from septic mice (n = 4-9/group) treated with or without rIGFBP6 (12.5μg/kg) at 6 or 24h post-CLP. Data are represented as mean values and are representative of 3 independent experiments; Each dot represents an individual; ns, not significant.

Supplemental Figure 10. Effect of IGFBP6 on chemokine production in experimental sepsis. Protein levels of CCL3, CXCL1, CXCL5, CXCL10, CXCL12 and CXCL16 in PLF and serum from septic mice (n = 6-9/group) treated with or without rIGFBP6 (12.5μg/kg) at 6 or 24h post-CLP. Data are represented as mean values and are representative of 3 independent experiments; Each dot represents an individual; ns, not significant.

Supplemental Figure 11. IGFBP6 suppresses STAT1-mediated CCL2 transcription

through JAK/STAT signaling modulation in epithelial cells.

(A) Western blot analysis of STAT1 phosphorylation in intestines of septic mice treated with PBS or rIGFBP6 (12.5μg/kg) post-CLP. **(B)** MLE-12 were treated with or without rIGFBP6 (200ng/mL), followed by stimulation with heat-inactivated *P.aeruginosa* (MOI = 100). WB analysis were performed for detection of p-STAT3, p-p65, p-IKBα, p-JNK, p-ERK1/2, p-P38 with indicated antibodies (The GAPDH blot in this panel is derived from the same experiment as the GAPDH blot shown in Figure 7E). **(C)** The phosphorylation of STAT1, STAT3, p65, IKBα, JNK, ERK1/2, P38 in MODE-K treated with/without rIGFBP6 at indicated time points after stimulation with heat-inactivated *P.aeruginosa* (MOI = 100). **(D)** CCL2 levels in MODE-K treated with STAT1 inhibitor 2-NP (10μM) or JAK agonist RO8191 (10μM) for 12h, quantified by ELISA (n = 6/group). **(E)** The binding site and mutant binding site of the CCL2 promoter. **(F)** EMSA assays were performed to detect the direct binding of STAT1 to the CCL2 promoter in MODE-K *in vitro*. **(G-H)** The PBS- or rIGFBP6-treated septic mice were intraperitoneally injected with/without 2-NP, the phosphorylation of STAT1 in intestinal (G) and lung tissues (H) were detected by WB. Data represent three independent biological replicates. Each dot in graphs represents data from an individual animal tissue and an individual well of cell culture. One-way ANOVA in (D); ns, not significant.

Supplemental Figure 12. IGFBP6 attenuates STAT1 signaling via PHB2 interaction.

(A) The expression of CCL2 in MLE-12 cells co-treated with rIGFBP6 (200ng/mL), and either rIGF2 or anti-IGF2, quantified by ELISA (*n* = 4/group). **(B)** Survival analysis of CLP-induced septic mice pre-treated with anti-IGF2 antibody (i.p.) followed by rIGFBP6 (12.5μg/kg, i.p.) or PBS administration (*n* = 11/group). Mortality was monitored for 14 days. **(C-D)** Western blot analysis of PHB2 expression in MLE-12 (C) and MODE-K (D) transfected with siNC or siPHB2. All data except (B) are representative of three independent experiments. Each dot in graphs represents data from an individual mouse, an individual well of cell culture. One-way ANOVA in (A); Log-rank test in (B); ns, not significant.

Supplemental Figure 13. IGFBP6 has no direct antibacterial activity.

(A-B) Growth kinetics of *P.aeruginosa* (A) and *S.aureus* (B) in Luria-Bertani (LB) broth containing rIGFBP6 (50/100/200 ng/mL) or PBS for the indicated times at 37°C. Optical density at 600 nm (OD600) were measured every 30 minutes.

Supplemental Figure 14. IGFBP6 has no effects on neutrophils antimicrobial functions during sepsis.

(A-B) Neutrophils (*n* = 4/group) were treated with or without rIGFBP6 (50/200 ng/mL) for 3h, and then, *in vitro* bacterial phagocytosis (A) and killing (B) of *P.aeruginosa* were analyzed. All data are representative of three independent experiments. Each dot in graphs represents data from an individual well of cell culture. One-way ANOVA in (A, B); ns, not significant.

Supplemental Figure 15. Effect of IGFBP6 on cytokine production by macrophages.

(A-B) PMs (5×10^5 cells, n = 4/group) were pretreated with rIGFBP6 (200ng/mL) for 4h, followed by *P.aeruginosa* challenge for 6h. IL-6 (A) and TNF-α (B) levels in supernatants were quantified by ELISA. **(C-D)** BMDMs (5×10^5 cells, n = 3/group) were subjected to identical stimulation conditions, with subsequent measurement of IL-6 (C) and TNF-α (D) secretion. **(E-F)** HMDM (5×10^5 cells, n = 3/group) subjected to identical stimulation conditions, with subsequent measurement of IL-6 (E) and TNF-α (F). Data represent three independent biological replicates with individual data points indicating separate culture wells. One-way ANOVA in (A-F); ns, not significant.

Supplemental Figure 16. IGFBP6 impairs antibacterial functions of macrophages by inhibiting Akt pathway. (A) Effect of Akt agonist SC79 on the viability of peritoneal macrophages. Peritoneal macrophages were incubated with serial concentrations of SC79 for 24h, and the viability was assessed by MTT test. **(B)** Peritoneal macrophages were treated with SC79 (25μM) for the indicated times, and then WB analysis of Akt signaling pathway were performed.
