# Supplementary material for: IGFBP6 orchestrates antiinfective immune collapse in murine sepsis via prohibitin-2–mediated immunosuppression
Source: J Clin Invest. 2025 Sep 2;135(21):e184721. doi: 10.1172/JCI184721 (PMC12578393; doi:10.1172/JCI184721)

Figure 7C
Lung

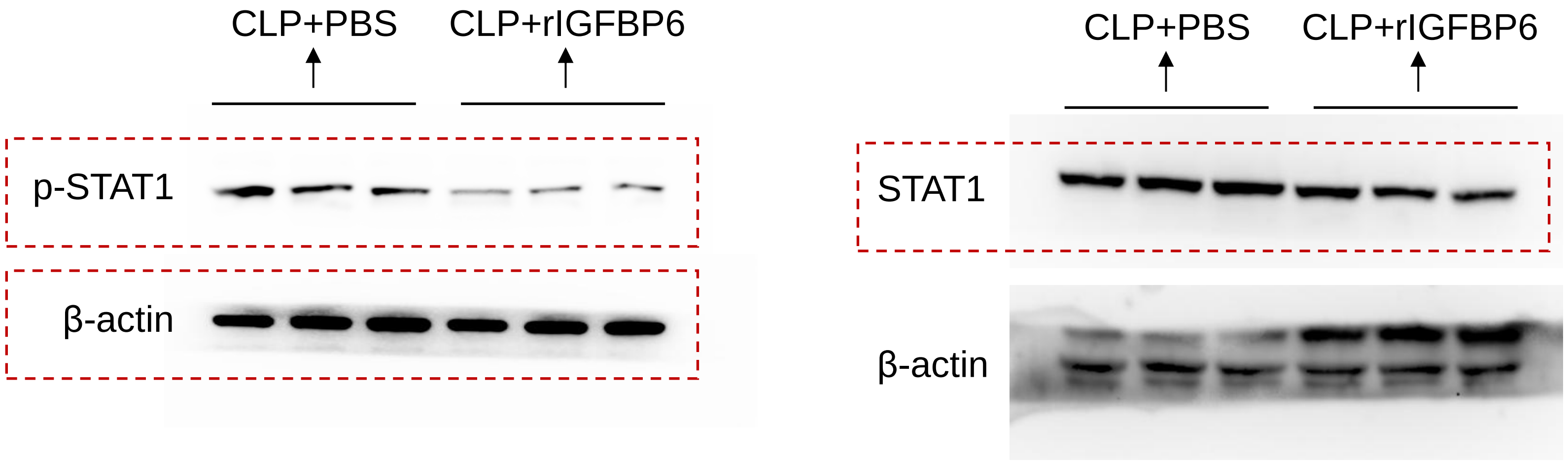

Figure 7E
MLE-12

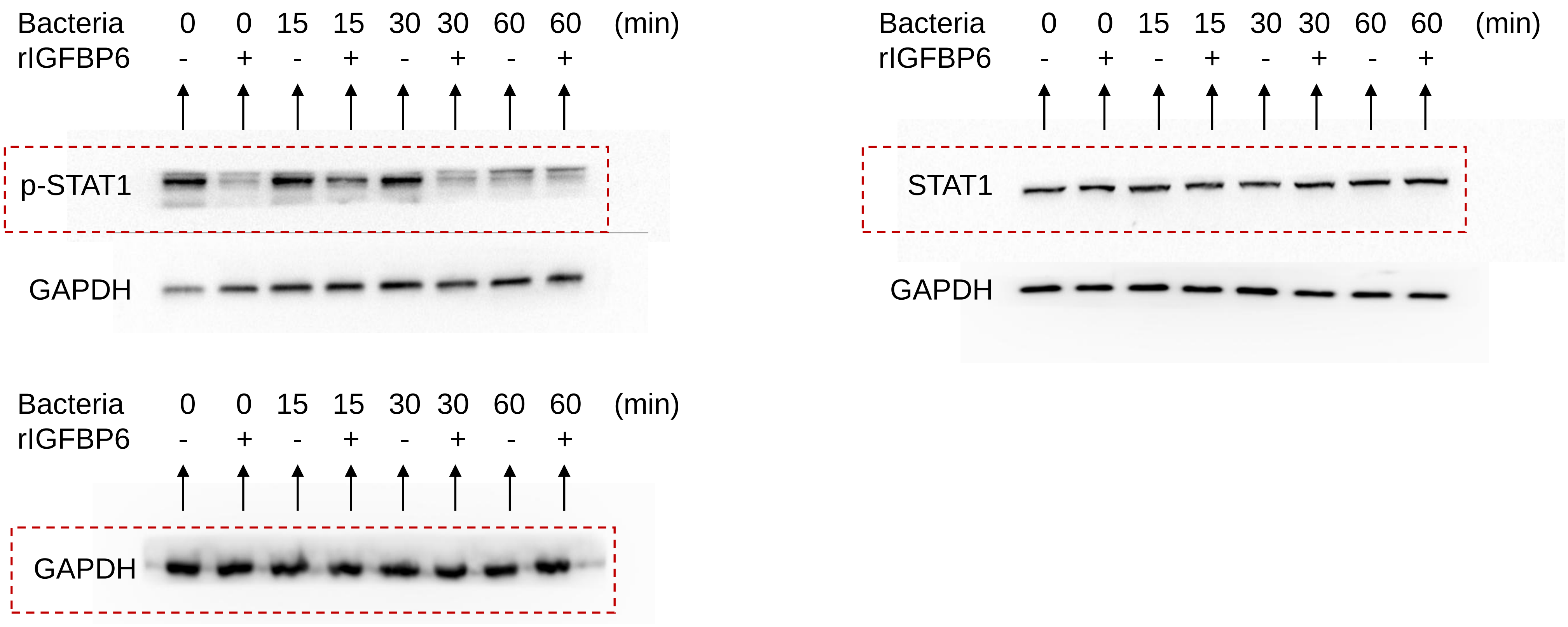

The GAPDH blot in this panel is derived from the same experiment as the GAPDH blot shown in Figure 11B.

Figure 7J
EMSA
MLE-12

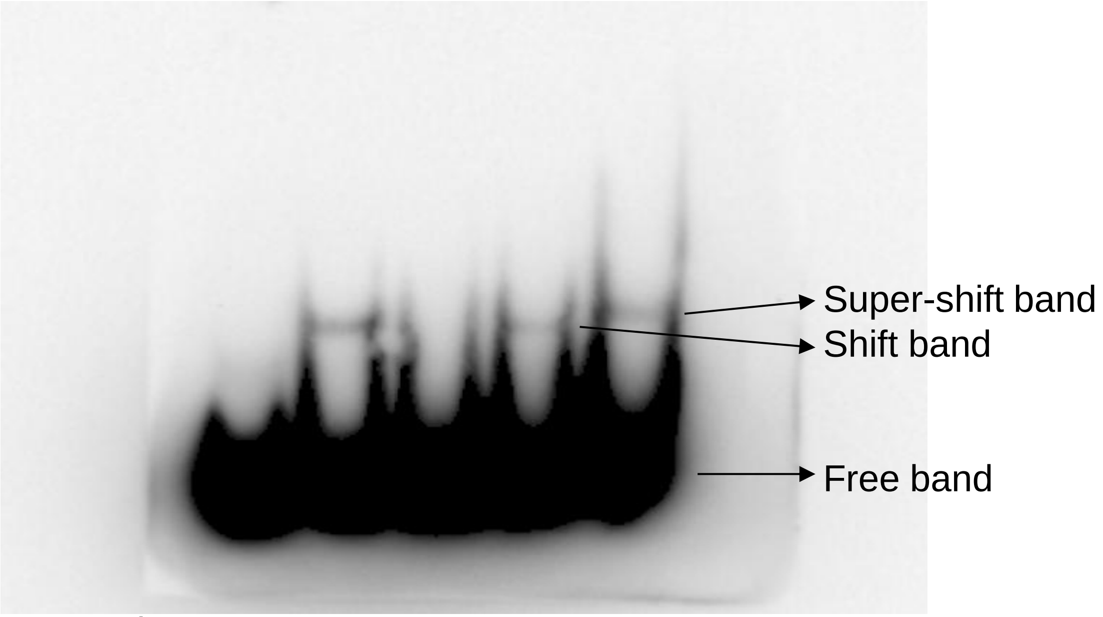

Probe	+	+	+	+	+
Nucleoprotein	-	+	+	+	+
Competitor	-	-	+	-	-
Mut competitor	-	-	-	+	-
STAT1 antibody	-	-	-	-	+

Figure 8C
MLE-12

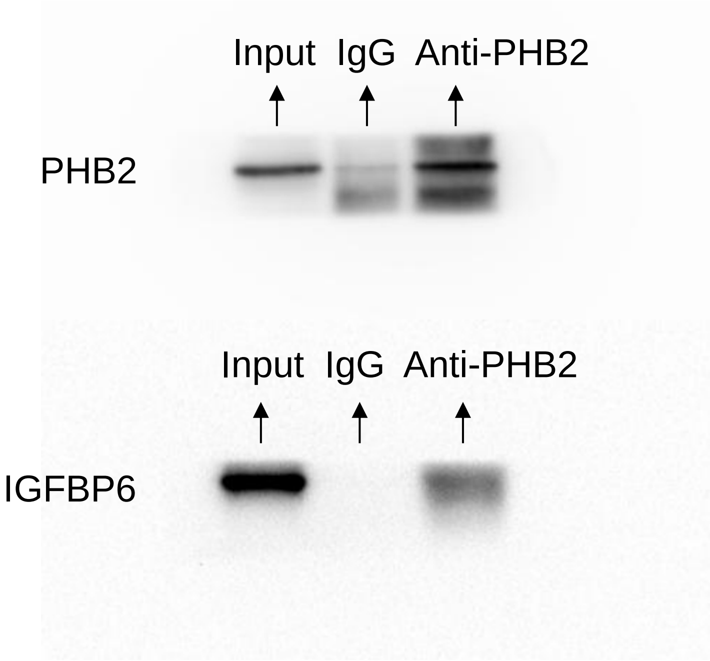

Figure 8D
MODE-K

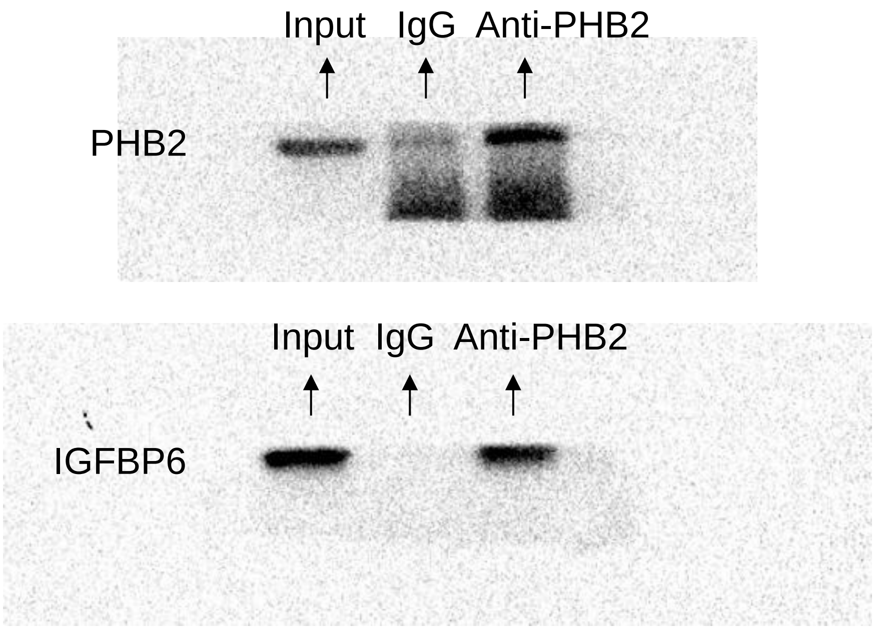

Figure 8E

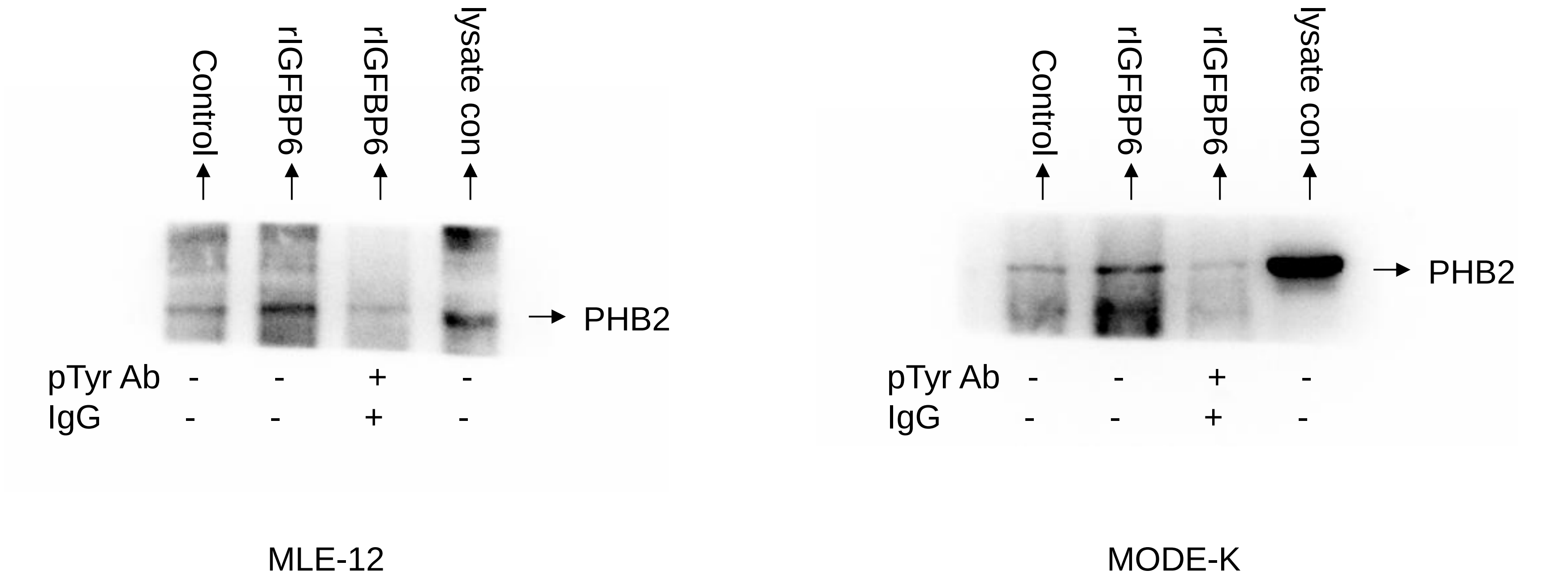
Control
rIGFBP6
rIGFBP6
lysate con
PHB2
pTyr Ab - - + -
IgG - - + -
MLE-12
Control
rIGFBP6
rIGFBP6
lysate con
PHB2
pTyr Ab - - + -
IgG - - + -
MODE-K

Figure 8G
MLE-12

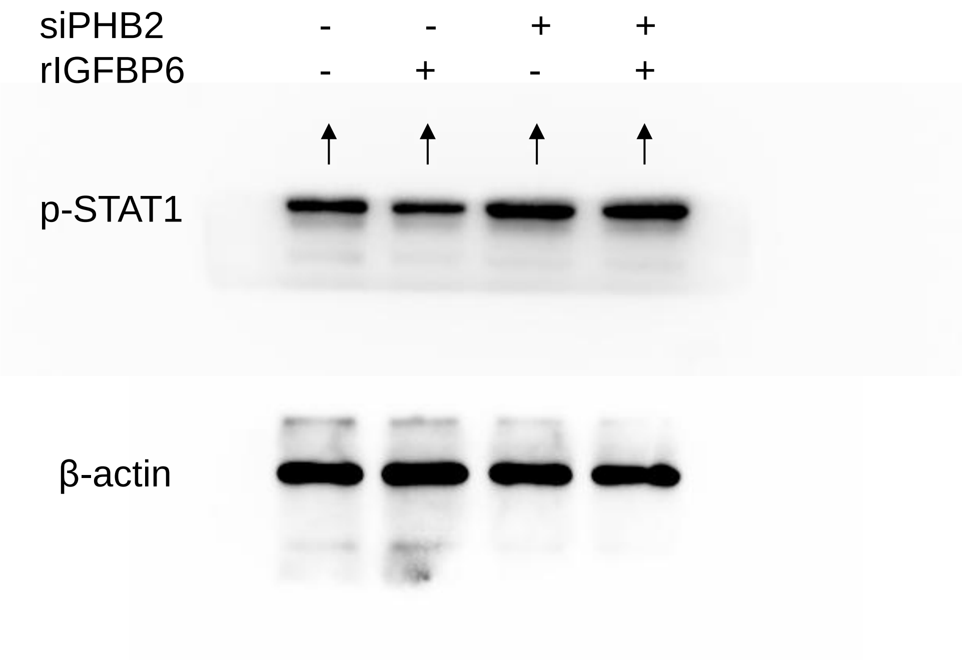

Figure 8H
MODE-K

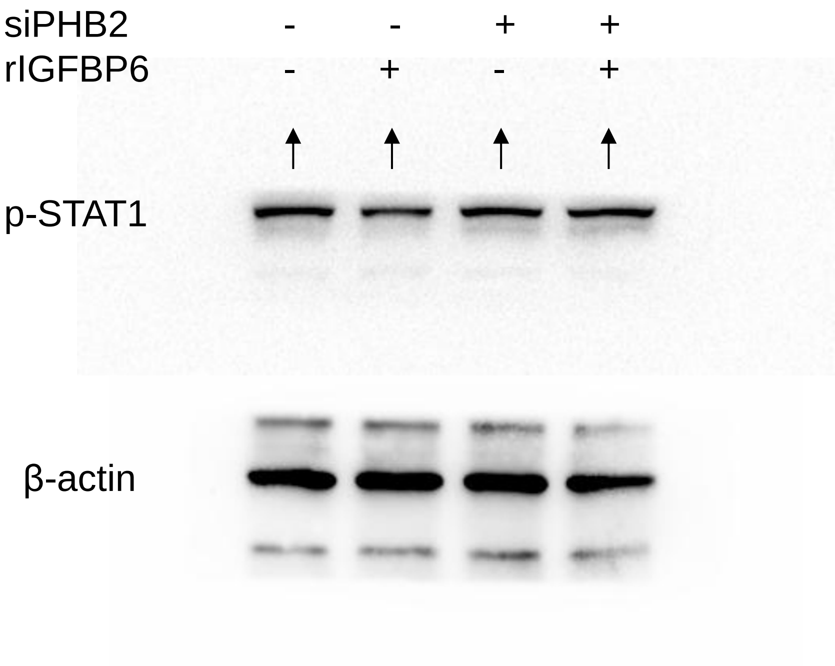

Figure 8J
MLE-12

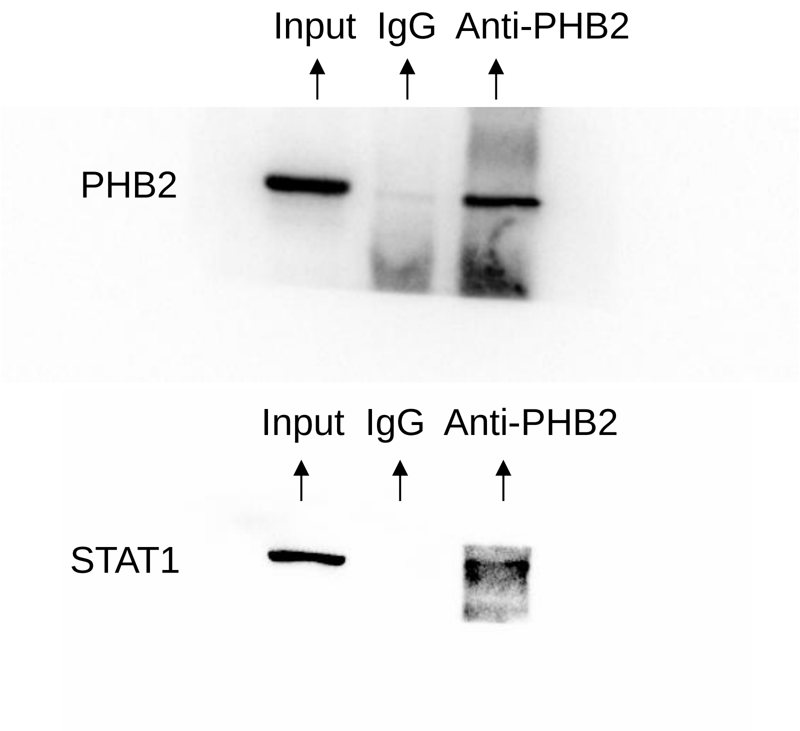

Figure 8K
MODE-K

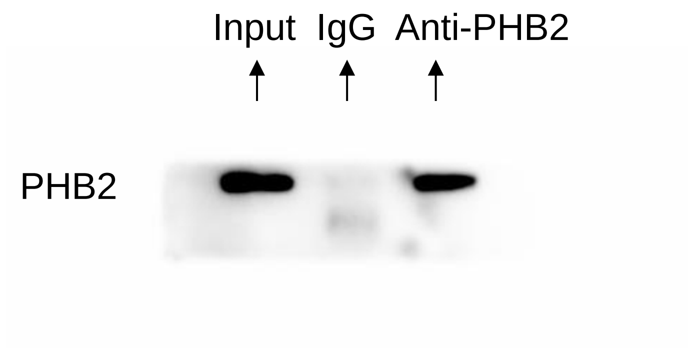

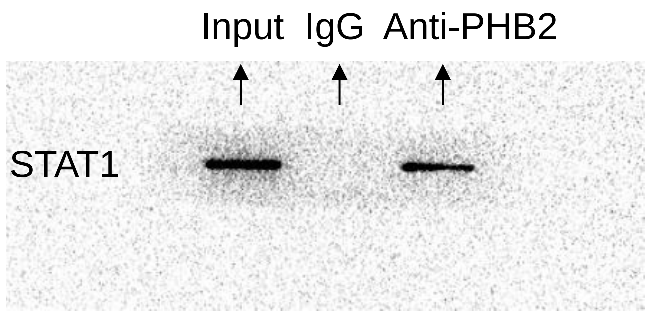

Figure 8N
Lung

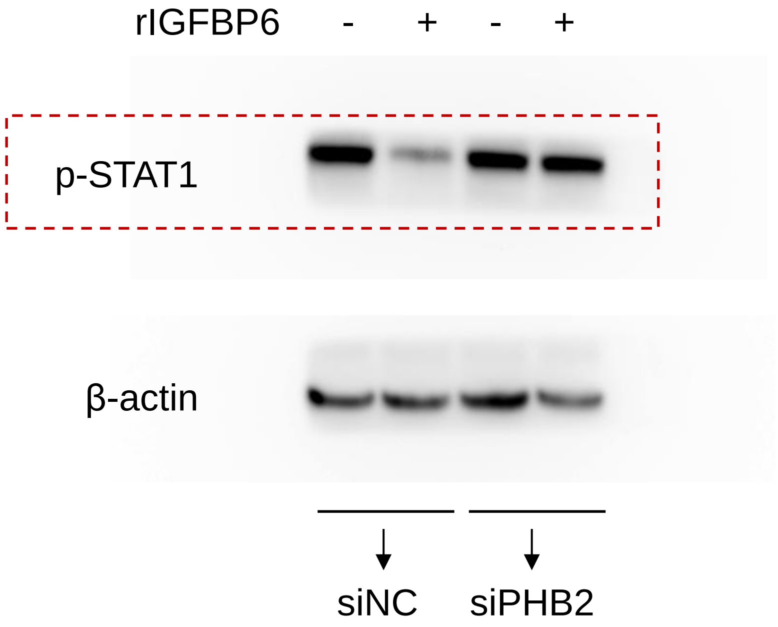

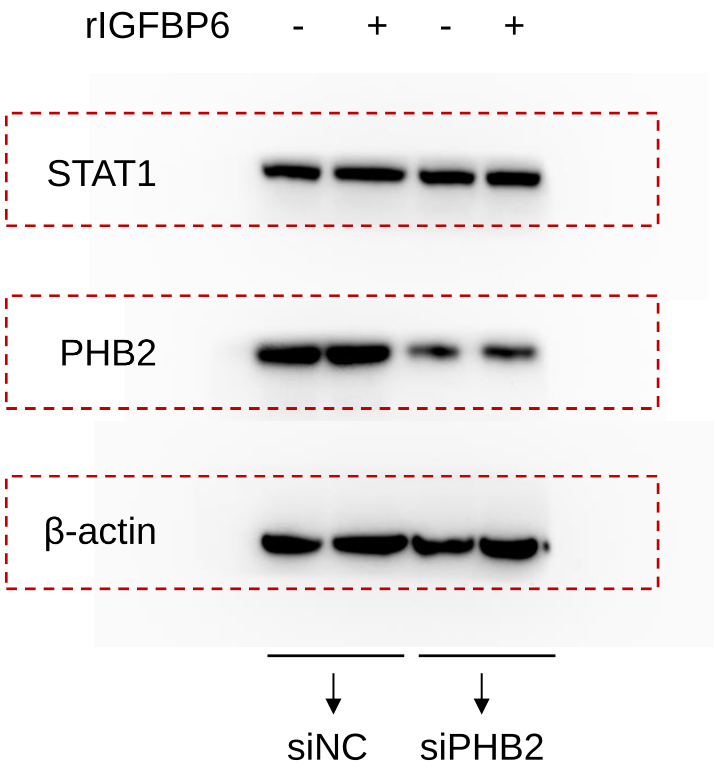

Figure 8O
Intestines

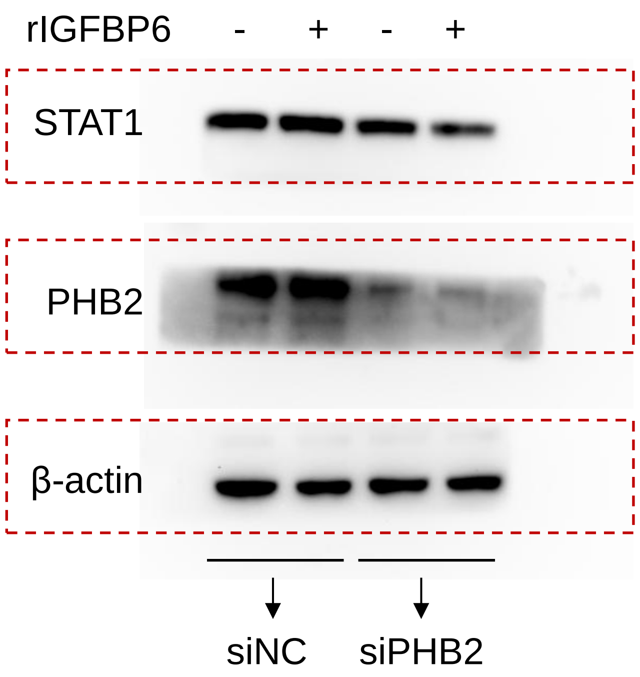

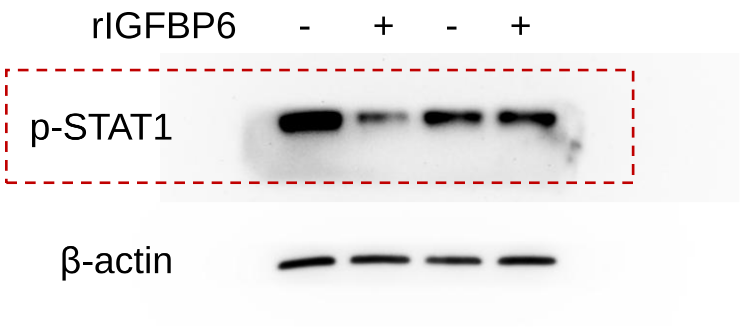

Figure 10C
macrophage

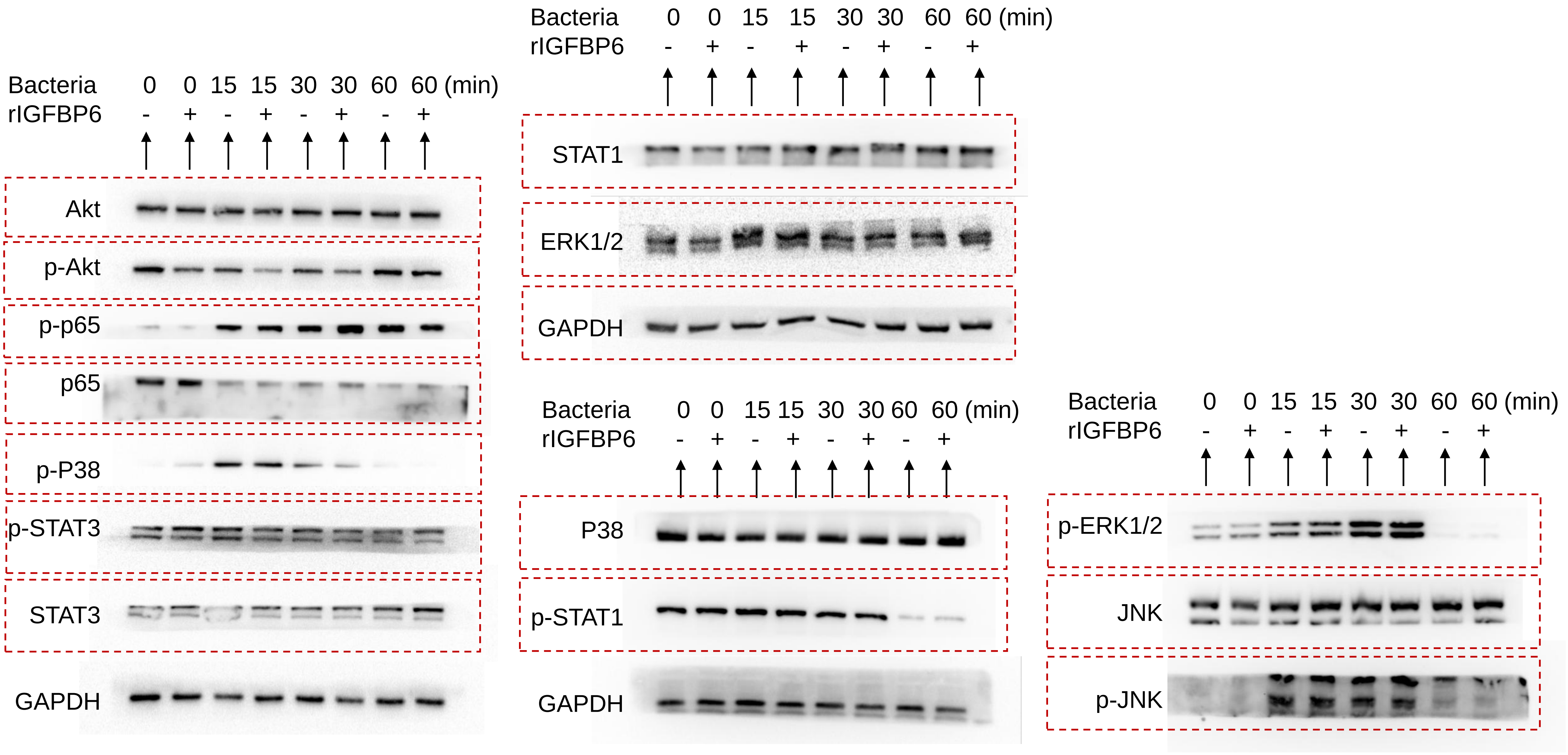

Supplemental Figure 11A
Intestines

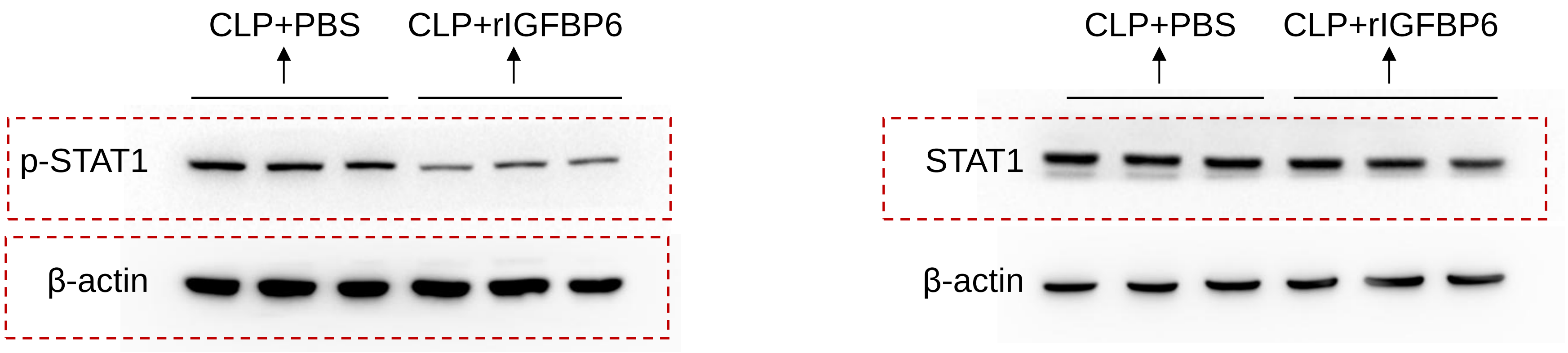

Supplemental Figure 11B
MLE-12

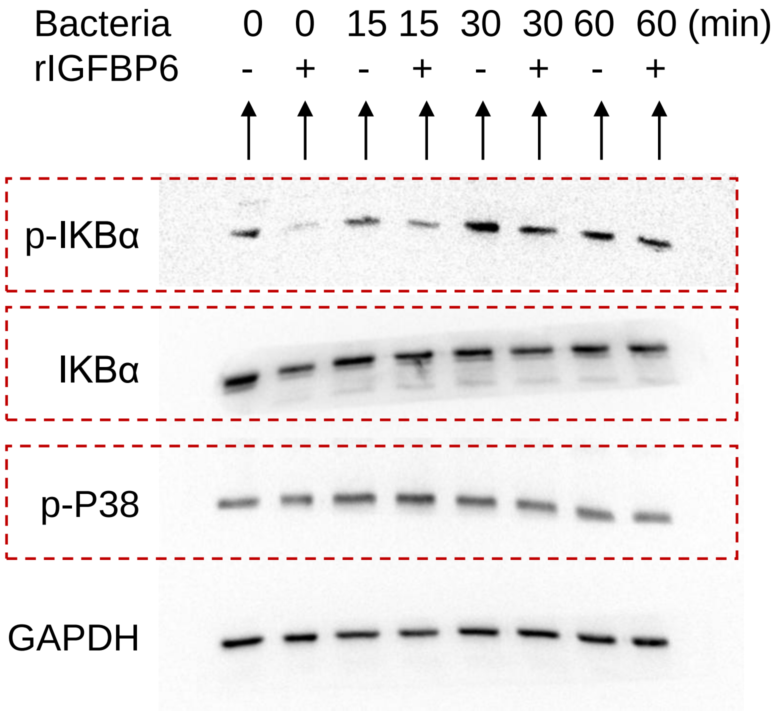

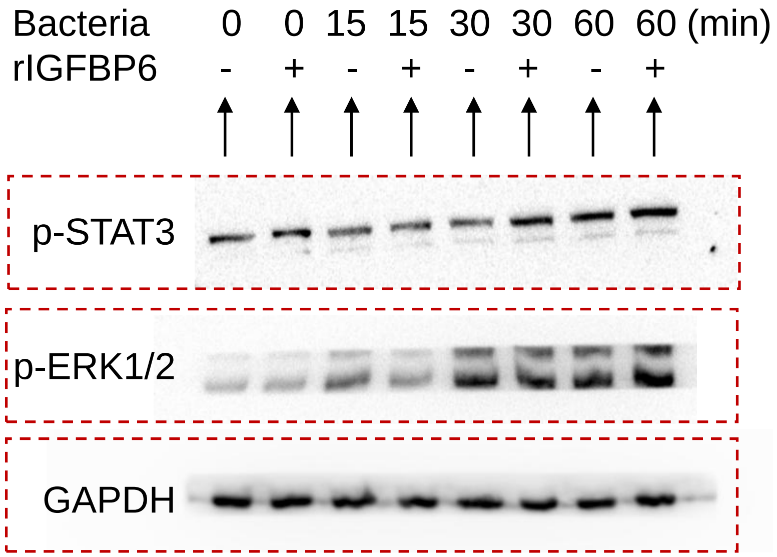

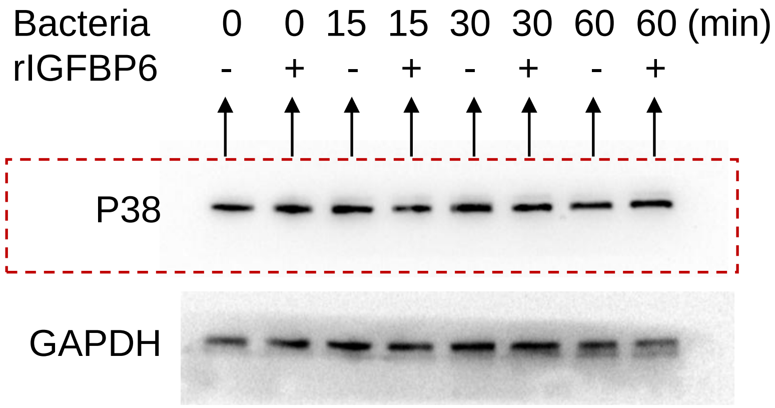

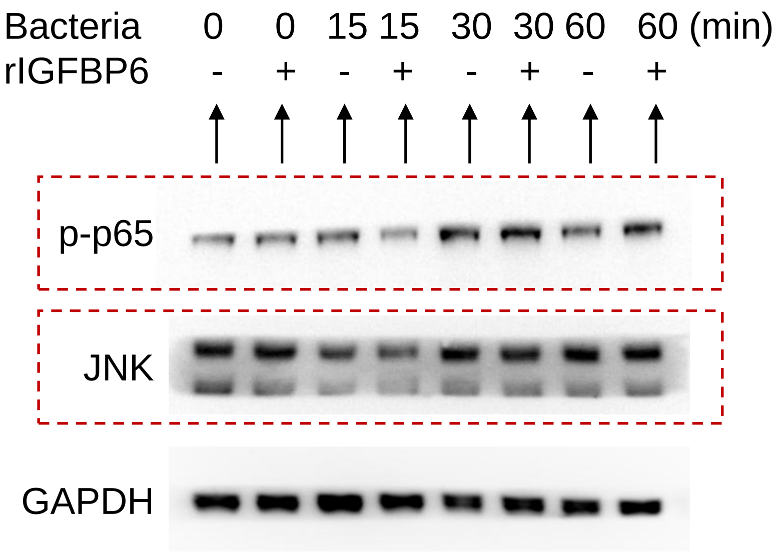

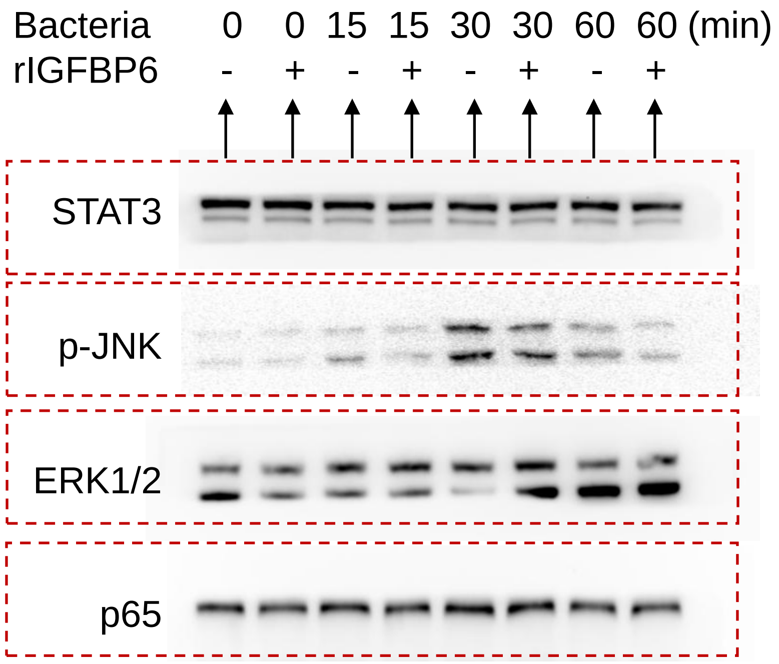

The GAPDH blot in this panel is derived from the same experiment as the GAPDH blot shown in Figure 7E.

Supplemental Figure 11C
MODE-K

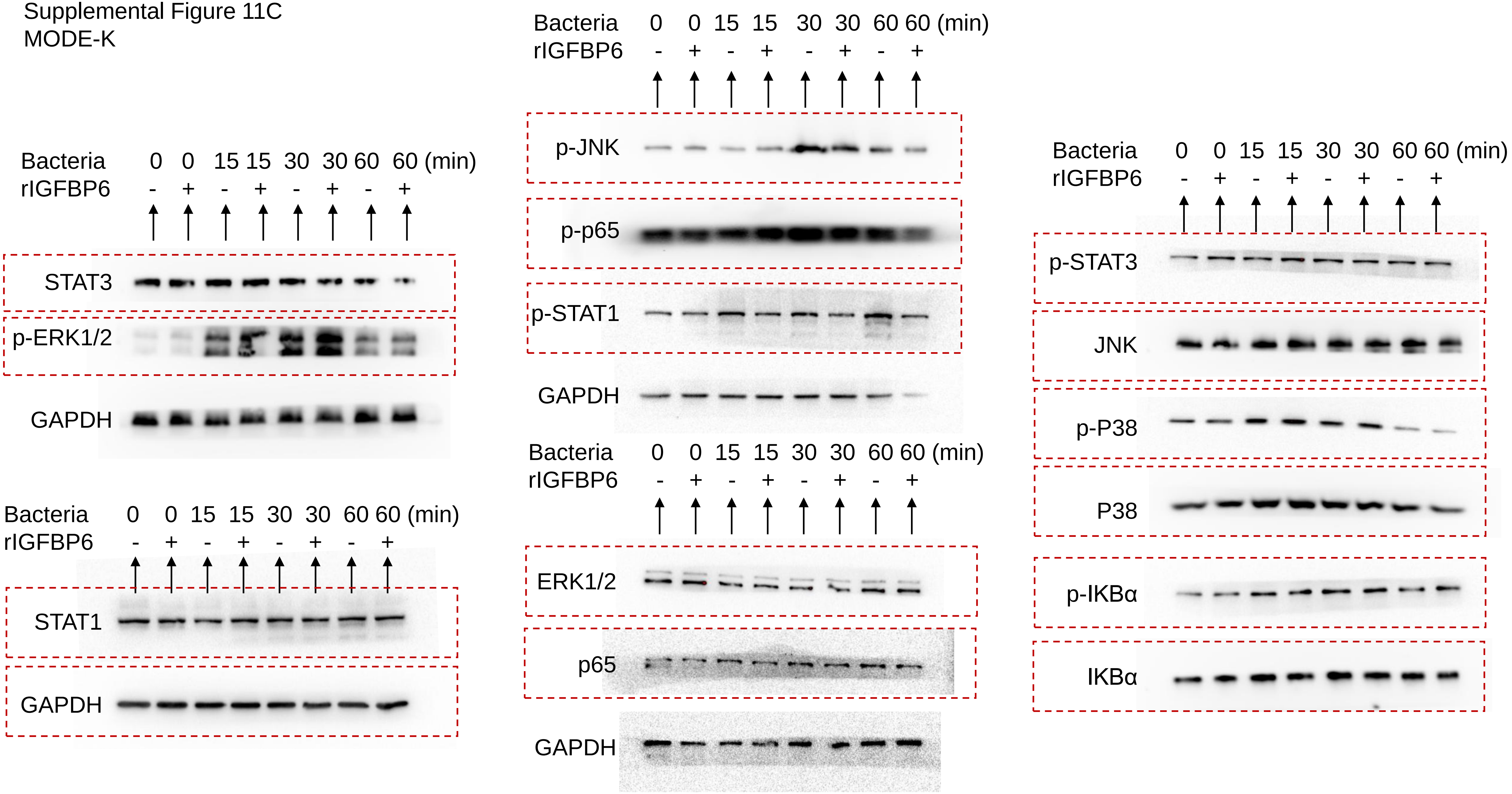

Supplemental Figure 11G
EMSA
MODE-K

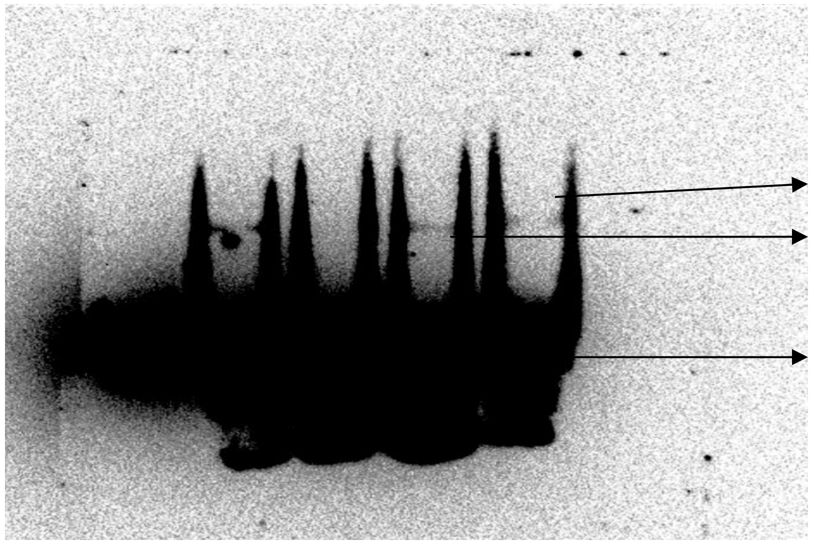

Probe	+	+	+	+	+
Nucleoprotein	-	+	+	+	+
Competitor	-	-	+	-	-
Mut competitor	-	-	-	+	-
STAT1 antibody	-	-	-	-	+

Supplemental Figure 11H
Lung

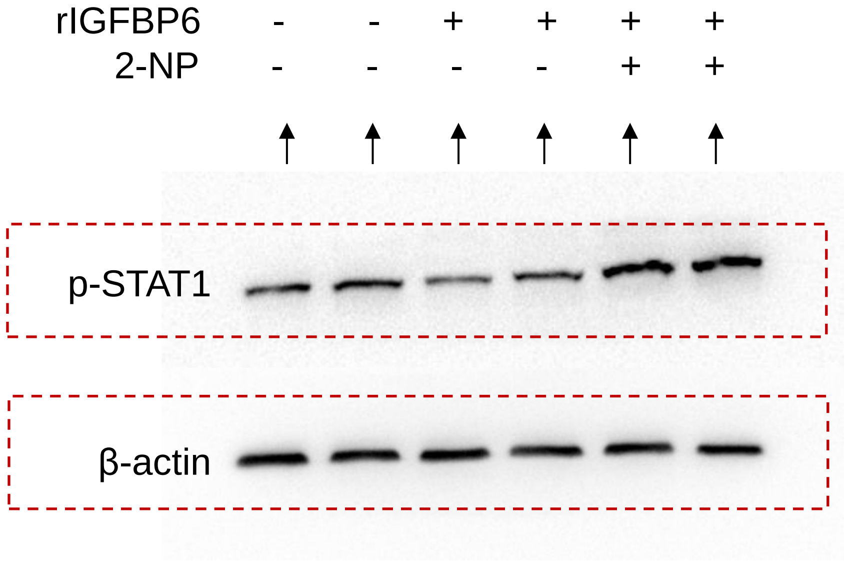

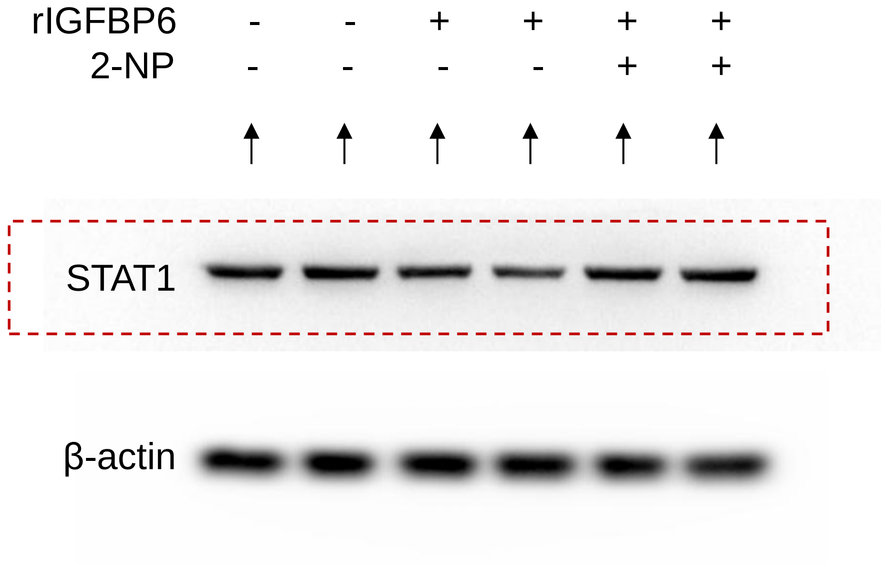

Supplemental Figure 11I
Intestines

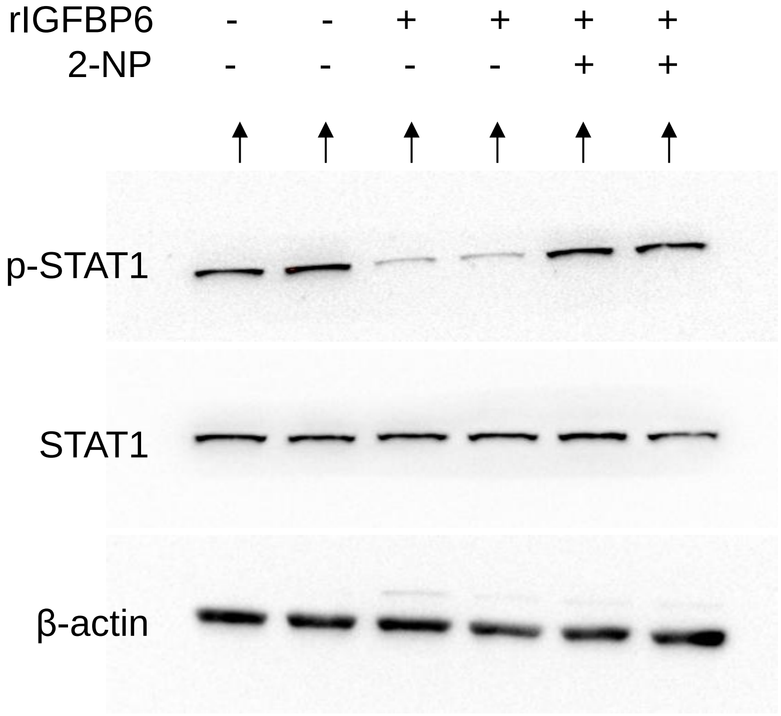

Supplemental Figure 12C
MLE-12

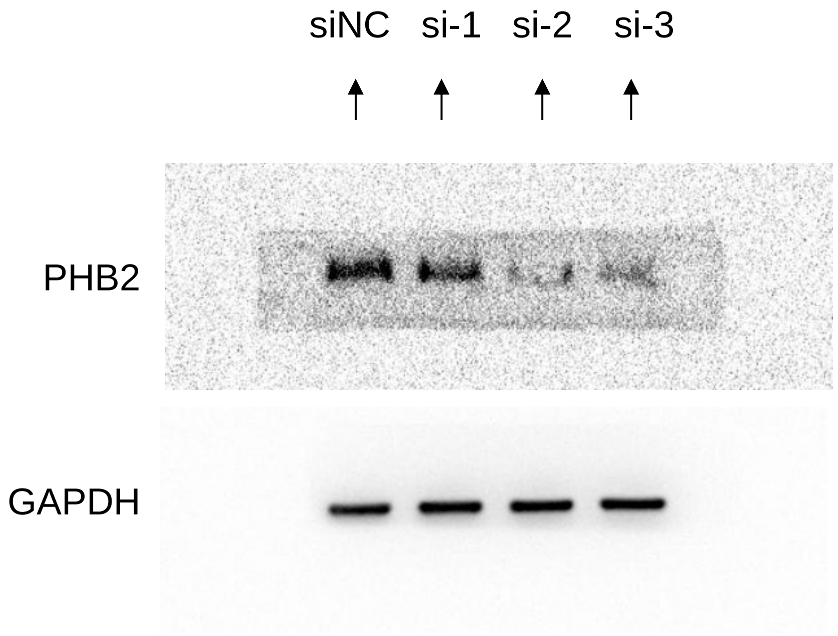

Supplemental Figure 12D
MODE-K

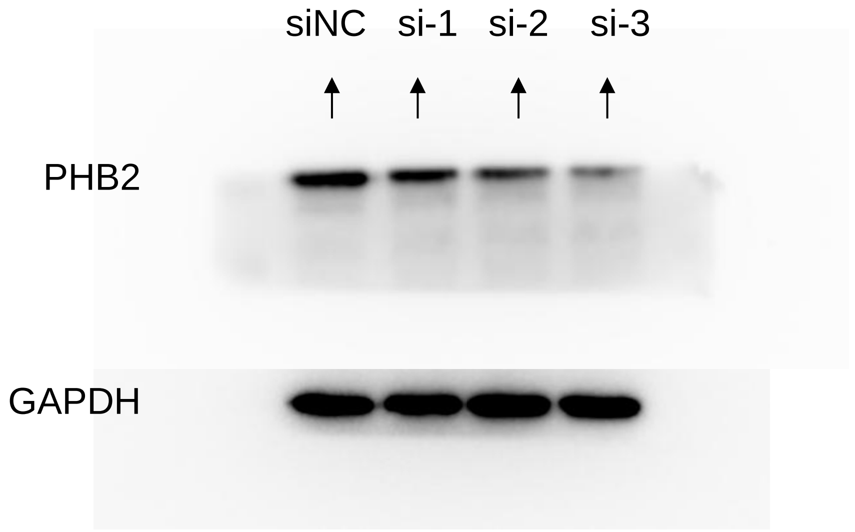

Supplemental Figure 16B
Macrophage

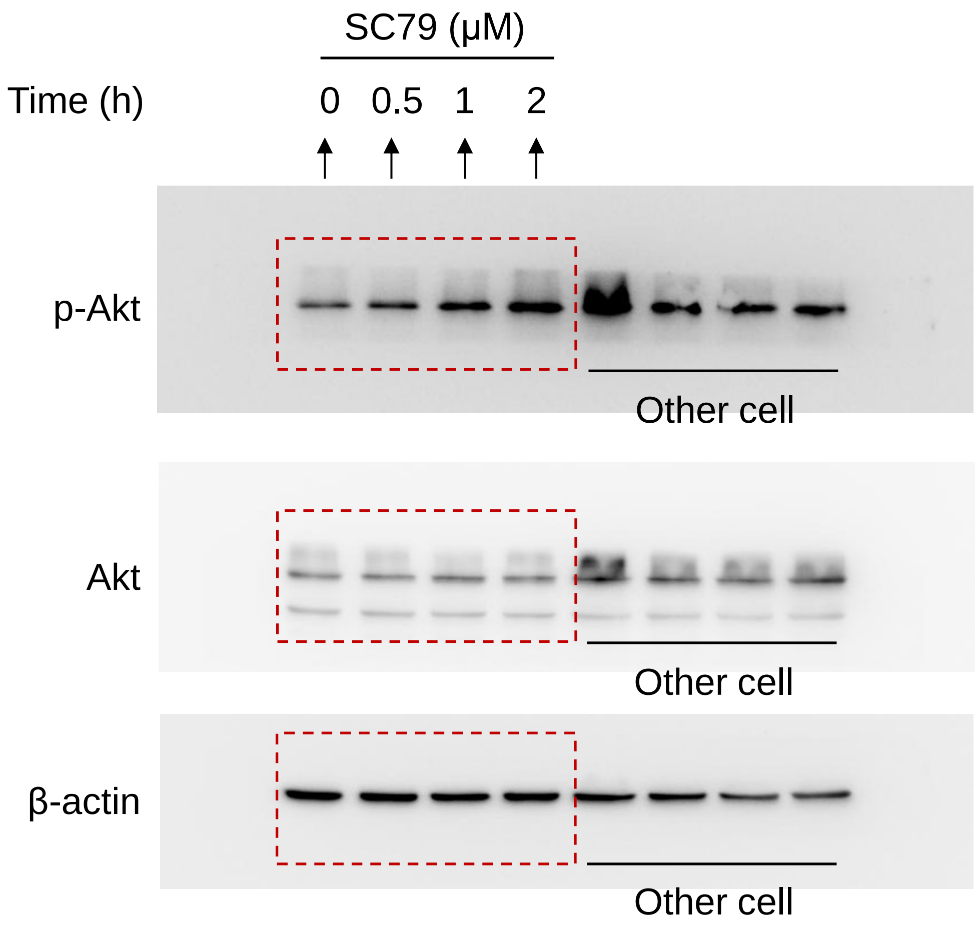

Supplement: Unedited blot and gel images [file jci-135-184721-s343.pdf]
